# The Discovery of Highly Efficient and Promising ABA Receptor Antagonists for Agricultural Applications Based on APAn Modification

**DOI:** 10.3390/molecules29133129

**Published:** 2024-07-01

**Authors:** Xiaobin Li, Xianjun Tang, Mian Wang, Xueqin Zhang, Yanjun Xu, Yiyi Li, Jiaqi Li, Zhaohai Qin

**Affiliations:** 1 College of Science, China Agricultural University, Beijing 100193, China; 2 College of Food and Bioengineering, Xihua University, Chengdu 610039, China; 3 College of Biological Sciences, China Agricultural University, Beijing 100193, China

**Keywords:** abscisic acid, ABA receptor antagonist, seed germination, abiotic stress

## Abstract

Abscisic acid (ABA) is one of the many naturally occurring phytohormones widely found in plants. This study focused on refining APAn, a series of previously developed agonism/antagonism switching probes. Twelve novel APAn analogues were synthesized by introducing varied branched or oxygen-containing chains at the C-6′ position, and these were screened. Through germination assays conducted on *A. thaliana*, colza, and rice seeds, as well as investigations into stomatal movement, several highly active ABA receptor antagonists were identified. Microscale thermophoresis (MST) assays, molecular docking, and molecular dynamics simulation showed that they had stronger receptor affinity than ABA, while PP2C phosphatase assays indicated that the C-6′-tail chain extending from the 3′ channel effectively prevented the ligand–receptor binary complex from binding to PP2C phosphatase, demonstrating strong antagonistic activity. These antagonists showed effective potential in promoting seed germination and stomatal opening of plants exposed to abiotic stress, particularly cold and salt stress, offering advantages for cultivating crops under adverse conditions. Moreover, their combined application with fluridone and gibberellic acid could provide more practical agricultural solutions, presenting new insights and tools for overcoming agricultural challenges.

## 1. Introduction

Abscisic acid (ABA, 1) is an extensively distributed sesquiterpenoid endogenous plant hormone that is classified as one of the many phytohormones naturally present in plants [1]. ABA plays a significant role in numerous physiological processes related to plant growth and development, including the inhibition of seed germination and seedling growth, induction of stomatal closure, and promotion of fruit ripening and coloring [2,3,4]. Furthermore, ABA also plays a crucial role in enhancing plant resistance against a multitude of biotic and abiotic stresses, such as phytopathogens, drought, flooding, salt, and cold. Due to these properties, ABA has great potential for agricultural applications [5,6,7,8,9,10,11].

The physiological effects of ABA are regulated through the signal transduction of ABA receptors PYR/PYL/RCARs (PYLs), which belong to the START superfamily of ligand-binding proteins [12,13]. PYLs possess a conserved hydrophobic ABA binding pocket containing two conserved domains, namely the gate loop and the latch loop, located on the outside of the pocket entrance [14]. Upon binding of ABA to the PYLs, a conformational change occurs in the gate and latch loops, transitioning them from an “open” to a “closed” state, thereby activating the PYLs [15]. The activated PYLs then form a new binding interface that interacts with the catalytic interface of the downstream PP2Cs (group A protein phosphatases 2C) [16,17]. This interaction results in the inhibition of the dephosphorylation via PP2Cs of the downstream component SnRK2 kinases (subfamily-2 members of SNF1-related kinases), thereby triggering a series of ABA responses in plants [18]. Concurrently, when the PYL-ABA complex binds to PP2Cs, a conserved tryptophan (Trp300 in ABI1) located on the recognition loop of the PP2C is inserted between the gate loop and the latch loop. The NH group of its indole ring forms a water-mediated hydrogen bond with the carbonyl group of ABA, effectively “locking” this interaction. This action contributes to the establishment of the well-known “gate–latch–lock” mechanism, which is responsible for ABA sensing and initiating signal transduction [19].

**Figure 1:**
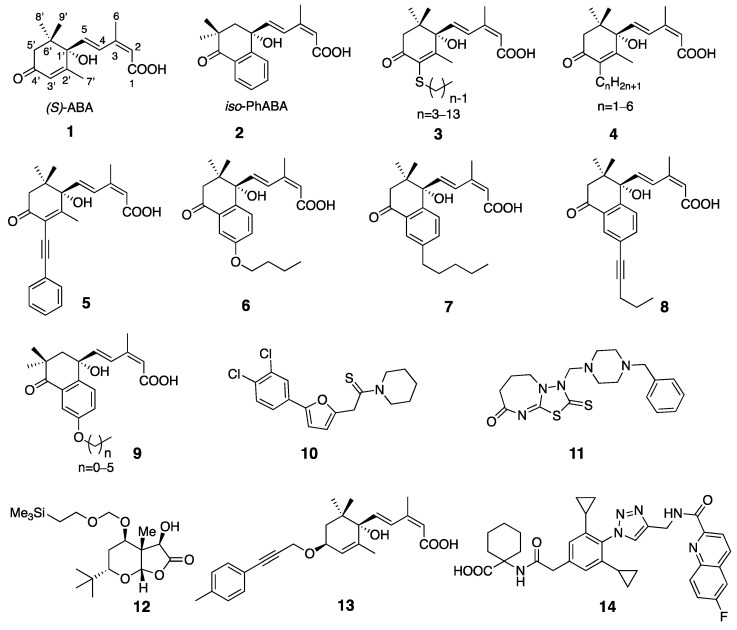
ABA and ABA receptor antagonists identified to date.

An intriguing discovery by Takeuchi et al. revealed that when ABA binds to the ligand binding pocket of the PYLs, its 3′-C-H bond and 4′-C=O group align with hydrophobic channels delimited by two specific amino acid residues in the PYLs [20]. These channels, known as the 3′-tunnel and 4′-tunnel respectively, lead directly to the interface between the PYLs and PP2Cs. This discovery prompted the development of a series of antagonists (Figure 1) targeting the 3′-tunnel, including ASn (**3**) [21], 3′-alkyl ABA (**4**) [22], 3′-phenyl alkynyl-ABA (**5**) [23], PAOn (**6**), PAC4 (**7**), PAT3 (**8**) [24], and APAn (**9**) [25], as well as antagonists based on the 4′-tunnel, such as PANMe (**13**) [26] and ANT (**14**) [27]. Consequently, there was a surge in the exploration of ABA receptor antagonists. Moreover, in addition to the designed 3′-tunnel and 4′-tunnel antagonists, other types of antagonists were also identified, such as DFPM (**10**) [28], AA1 (**11**) [29], and RK460 (**12**) [30].

ABA receptor antagonists, as regulators of ABA receptor activity, demonstrate significant potential for agricultural applications. These applications include enhancing transpiration and gas exchange in greenhouse agriculture under high concentrations of carbon dioxide [31], investigating ABA-dependent physiological processes in crops through inhibiting ABA signal transduction [19], improving fluoride tolerance in crops [32], and regulating germination rates, emergence, and seedling growth under stressful ecological conditions [33].

Previously, we introduced APAn as a class of ABA receptor agonist/antagonist switching probe that can modulate agonistic or antagonistic activity based on the length of a 6′-alkoxyl chain [25]. As the alkoxyl chain extends from APA1 to APA6, the binding potential to the receptor gradually increases while the inhibition activity against the HAB1 decreases. Although APA6 exhibits potent antagonistic activity against PYLs, it remains unclear whether longer alkyl chains are the most suitable for the 3′-tunnel, and how the channel environment affects their activity. To investigate the influence of the 3′-tunnel environment on these antagonists and to develop more efficient and promising agricultural ABA receptor antagonists, we conducted careful modifications of the alkyl chain in APAn, including the introduction of branches or heteroatoms. This allowed us to obtain additional information about the tunnel environment. The obtained results offer valuable insights for the development of 3′-tunnel ABA receptor antagonists.

## 2. Results and Discussion

### 2.1. Chemistry

**Figure 2:**
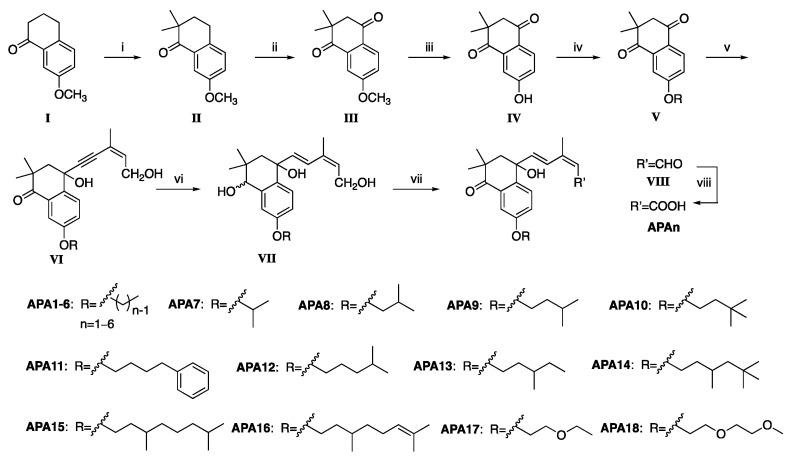
Synthetic pathway of target compounds. Reagents and conditions: i. CH_3_I, NaH/THF, 0 °C, 12 h; ii. Co(acac)_2_, t-BuOOH/acetone, r.t., 48 h; iii. AlCl_3_/toluene, reflux, 3 h; iv. R-Br or R-I or R-OTf, K_2_CO_3_/MeCN, reflux, 12 h; v. (*Z*)-methylpent-2-en-4-yn-1-ol, n-BuLi/THF, −78 °C, 0.5 h to r.t., 3 h; vi. Red-Al/THF, 0 °C to r.t., 2 h; vii. DMP/DCM, r.t. 0.5 h; viii. NaClO_2_, NaH_2_PO_4_, 2-methyl-2-butene, t-BuOH/H_2_O, r.t. 0.5 h.

The synthesis process is presented in Figure 2. Initially, 7-methoxy-3,4-dihydronaphthalen-1(2 H)-one served as the starting material and was subjected to treatment with sodium hydride and iodomethane, resulting in the formation of intermediate II. This intermedate was then oxidized using t-butyl hydroperoxide and Co(acac)_2_ as a catalyst to give intermediate III. Subsequently, it was treated with anhydrous aluminum trichloride and refluxed to remove the methyl group and give intermediate IV. Intermediate IV was reacted with 1-iodoalkyl, 1-bromoalkyl, or *p*-toluenesulfonate, along with potassium carbonate in acetonitrile, under reflux conditions to give intermediate V. Through a nucleophilic addition reaction between intermediate V and alkynyl lithium (synthesized in situ using (*Z*)-3-methylpent-2-en-4-yn-1-ol and n-butyl lithium), and primary alcohol VI was obtained. This intermediate was then selectively reduced using Red-Al to give intermediate VII, where the triple bond and carbonyl groups were reduced. Further oxidation of alcohol VII to aldehyde VIII was achieved through Dess−Martin oxidation. Finally, aldehyde VIII was converted to the target compound APAn through Pinnick oxidation.

### 2.2. Seed Germination Promotion Activity

#### 2.2.1. The Promotion Efficiency of APAn on *A. thaliana* Seed Germination

The agonistic/antagonistic activity of APAn was evaluated through the impact of compound treatment on seed germination. The treatment outcomes for the model plant *A. thaliana* are presented in Table 1, Table S1, and Figure S43. When applied individually, ABA and *iso*-PhABA exhibited strong inhibitory effects on seed germination; the germination rates within 48 h were 13.33% and 8.67%, respectively, significantly lower than the control (CK) rate of 91.33%. Based on the results of the three treatment groups, the following observations can be made: (1) among the linear alkyl tail chain compounds, APA1-APA3 showed a gradual decrease in agonistic activity, whereas APA4 and subsequent compounds demonstrated antagonistic activity. This indicated that the presence of a tail chain with four carbon atoms was the critical determinant of the agonistic/antagonistic activity of the APAn compounds, consistent with our previous research findings; (2) the introduction of a branched chain into the linear alkyl tail chain led to antagonistic activities in APA7 and APA8 compared with APA2 and APA3, while APA9 and APA10 exhibited weak antagonistic activities comparable to that of APA4. Conversely, APA12 and APA13, which possessed a backbone of five carbon atoms, exhibited significantly lower antagonistic activity than APA5. This suggested that the introduction of side chains at different positions along the tail chain significantly influenced the overall conformation outside the 3′-tunnel, thereby directly impacting the agonistic/antagonistic activities of the compounds; (3) the compounds with tail chains comprising six or more carbon atoms displayed similar antagonistic activities. This suggested that the carbon chains of six atoms appeared to be the most suitable length of tail chain for this type of antagonist; (4) interestingly, APA17, with a tail chain composed of five atoms, demonstrated activity comparable to compounds with a six-carbon chain but higher than that of APA5. This suggested that the introduction of oxygen atoms may have influenced the conformation of the binding surface with PP2C phosphatase through interaction with polar residues in the 3′-tunnel, ultimately affecting their binding affinity with PP2C.

#### 2.2.2. The Promotion Efficiency of APAn on Colza Seed Germination

The results of the seed germination of dicotyledonous colza are presented in Table 2 and Table S2, Figure S44. The following significant information was also derived from the data: (1) the agonistic activities of *iso*-PhABA, APA1 to APA3 obviously decreased; (2) compared with APA2/APA3, APA7/APA8 showed little difference in activity. Although the former displayed certain antagonistic activity against ABA, it was still unable to antagonize the influence of salt stress on colza seed germination; (3) although APA4 combined with ABA had a certain antagonistic effect, this effect was unable to antagonize the inhibition of salt stress on seed germination. Interestingly, compared with APA4, the antagonistic activity of APA9/APA10 against ABA was only slightly increased, but it greatly enhanced the germination rate of seeds under salt stress. This phenomenon did not appear in the A. thaliana assay, which deserves further investigation; (4) compared with APA5, the antagonistic activity of APA12/APA13 to ABA decreased slightly, but APA13 enhanced the germination rate of seeds under salt stress; (5) compared with APA5, APA17 significantly improved the antagonistic ability to ABA and the germination rate of colza seeds under salt stress, which once again showed that the introduction of oxygen atoms had a significant effect on improving the antagonistic activity of the compounds; (6) the compounds APA11/APA14-16 with a tail chain of six or more atoms all showed obvious ABA antagonistic activities and salt stress resistance, and their activities were equal to or slightly lower than that of APA6. The ABA antagonistic activity of APA18 was similar to that of APA6, but the germination rate of colza seeds under salt stress was slightly improved. This result reaffirms that a chain of six atoms is an appropriate length for the tail chain of APAn antagonists. Increasing the tail chain length beyond this point did not significantly impact the activity. In summary, the effects of APAn compounds on colza seed germination were generally comparable to their effects on *A. thaliana*, but there were some variations that warranted further investigation.

#### 2.2.3. The Promotion Efficiency of APAn on Rice Seed Germination

In the experiment involving monocotyledonous rice seed germination, two parameters were considered: germination potential, representing the percentage of seeds that germinated within 3 days, and germination rate, representing the percentage of seeds that germinated within 7 days. In the case of crop seeds, a high germination rate and strong germination potential indicate fast and uniform emergence, resulting in robust seedlings. Conversely, a high germination rate with a weak germination potential indicates uneven emergence and the development of weak seedlings, which is not ideal for agricultural cultivation [34].

To accurately assess the speed and uniformity of seed germination, the number of germinated seeds was counted daily to calculate the germination index. Additionally, the length of the cotyledons and roots of the seedlings were measured to calculate the vitality index. Both the germination index and vitality index provided valuable insights into the speed and uniformity of seed germination. A larger value for these two indexes indicated faster seed germination and greater uniformity in seedling emergence.

As shown in Table 3 and Table S3, (1) the germination potential, germination index, cotyledon and root length, and vitality index of rice seeds were far less than CK when ABA and *iso*-PhABA were applied alone, which indicated that they could strongly inhibit seed germination and rice seedling growth. From the results of the three treatments, it was found that all the treatments led to high germination rates, which also indicated that the quality of the rice seeds was high. In pure linear tail chain compounds, APA1–3 showed agonistic activity to rice seeds, while APA4–6 showed antagonistic activity. (2) When the straight chain tail chain was branched, APA7 and APA8 were associated with lower germination potential and germination index compared with ABA treatment, which had agonistic effects on rice seed germination and slowed down seed germination. However, from the vitality index, they were found to have antagonistic activities on seedling growth under ABA and salt stress. (3) APA9/APA10 antagonized the rice seed germination inhibition and seedling growth inhibition activities under salt stress, but this effect did not antagonize the germination inhibition caused by ABA. These results were different from those showing APA4’s ability to antagonize the germination inhibition and seedling growth inhibition activities caused by ABA and salt stress, indicating that APA4 was the turning point of agonist/antagonist activity. (4) Compared with APA5, APA12/APA13 and ABA showed considerable antagonistic activities affecting seed germination and seedling growth, while APA13 showed weaker antagonistic activity than APA5. APA12 and APA13 with five carbon atoms showed similar or slightly lower effects of antagonistic activities on seed germination potential than APA5, but unexpectedly, APA12 and APA13 showed significantly higher seedling growth inhibition than APA5 under antagonistic salt stress. Similarly, APA17 showed higher antagonistic activity than APA5, once again demonstrating that the introduction of oxygen atoms had a significant impact on the antagonistic activity. (5) The compounds APA6, APA11, and APA14–18 with a tail chain of six or more atoms had high germination potential, germination index, and vitality index, which showed that they were able to successfully antagonize the inhibitory effect of exogenous ABA on seed germination and seedling growth, accelerating seed germination and improving the uniformity of seedling emergence. In a word, APA6, APA11, and APA14–18 strongly antagonized the ABA-mediated inhibition of both seed germination and seedling growth under exogenous ABA and salt stress, accelerating seed germination and improving the uniformity of seedling emergence under adversity.

#### 2.2.4. The Promotion Efficiency of APAn on Wheat Seed Germination

The exposure of plants to low-temperature stress results in an increase of ABA content [35], which subsequently reduces the germination rate of seeds and delays the time of germination. As a consequence, seedling development is also delayed, which is unfavorable for agricultural production. ABA and gibberellin are the principal endogenous factors involved in the regulation of seed dormancy and germination. ABA promotes dormancy and inhibits seed germination, whereas gibberellin stimulates germination. Consequently, regulating the balance between ABA and gibberellin is crucial for controlling seed germination [36].

Therefore, we next investigated the effects of using fluridone (FD, an inhibitor of ABA biosynthesis), diniconazole (DCN, an inhibitor of ABA metabolism), paclobutrazol (PBZ, an inhibitor of gibberellin biosynthesis), gibberellin 3 (GA_3_, an antagonist of ABA functions), and APA18, alone or in combination, on wheat seed germination, cotyledon length, and root length under normal conditions and cold stress, and the results are summarized in Table 4. At first, at normal temperatures, the germination rate of seeds was slightly improved by applying FD, GA_3_, and APA18 alone, and the lengths of cotyledons and roots were equivalent to CK. The findings of this study clearly demonstrated that suppressing ABA synthesis (FD) and ABA signaling (APA18), as well as antagonizing ABA function, were advantageous in overcoming ABA-mediated seed dormancy. Furthermore, the combined utilization of these compounds could also produce the expected synergistic effect. Conversely, inhibiting ABA metabolism (DCN) and gibberellin biosynthesis (PBZ) exhibited an opposite effect, exacerbating ABA-mediated seed dormancy.

These effects were more obvious under low-temperature stress (Table 4). At 4 °C, the germination rate of CK was 78.50%, whereas those of the FD, GA_3_, and APA18 treatment groups were 90.27%, 87.40% and 91.09%, respectively. At the same time, the cotyledon length and root length also increased greatly. These results highlight the potential for agricultural application in order to promote seed germination and seedling growth.

### 2.3. Influence on Stomatal Movement

**Figure 3:**
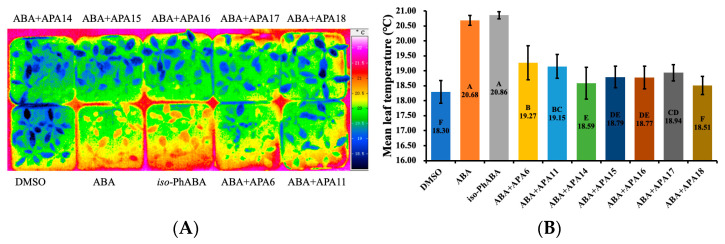
(**A**) Impacts of different treatments (in the presence of 5 μM ABA, *iso*-PhABA, or 5 μM ABA + 25 μM of APAn) on stomatal movement in *A. thaliana* (infrared thermography photos); (**B**) average leaf temperature of *A. thaliana* under different treatments. Values represent means ± SD, and those marked with the same letter are statistically significant (*p* < 0.05).

Stomata serve as the primary sites of transpiration, playing a crucial role in the expelling of water vapour and facilitating the exchange of CO_2_/O_2_. The release of water vapor can effectively reduce leaf temperature and protect leaves against sunburn caused by sunlight. Under adverse conditions such as drought or cold stress, the biosynthesis of ABA is promoted, resulting in stomatal closure. While stomatal closure helps to protect plants, it also hinders biosynthesis due to a decrease in photosynthesis, which has a negative impact on yield improvement [37,38]. Therefore, it becomes necessary to inhibit ABA-mediated stomatal closure in certain situations, particularly in greenhouse cultivation [39]. In order to ascertain the impact of APAn on stomatal movement, infrared thermal imaging technology was employed (Figure 3A). Our results showed that both ABA and *iso*-PhABA significantly induced stomatal closure in *A. thaliana* compared with the control, resulting in a reduction in transpiration and a marked increase in leaf temperature (Figure 3B). In the groups where ABA and APAn (APA6, APA11, or APA14–18) were applied in combination, the leaf temperature was significantly lower than with ABA alone. Notably, APA14 and APA18 even approached the level of the control, indicating their strong, albeit not complete, antagonistic effects on ABA-mediated stomatal closure. These compounds demonstrated the potential to enhance crop biomass and yield under greenhouse cultivation conditions.

### 2.4. Receptor-Binding Affinity and HAB1 Phosphatase Activity

Whether as an agonist or antagonist, the first step involves binding to the ABA receptors. In order to investigate the relationship between the receptor affinity of APAn, the tail chain structure, and antagonistic activity, we utilized the microscale thermophoresis (MST) method to measure the affinity of several antagonists with high activities towards ABA receptors. The receptor proteins used for this study were PYR1, PYL2, PYL3 (subgroup III), PYL6 (subgroup II), and PYL10 (subgroup I) (Table 5). The results indicated that both ABA and *iso*-PhABA exhibited strong affinity to these receptors. Furthermore, the strong inhibitory activities of the ABA/*iso*-PhABA-PYLs binary complex towards HAB1 phosphatase demonstrated the reliability of the prepared receptor proteins. All other APAn compounds exhibited stronger receptor affinity than *iso*-PhABA, suggesting that the 6′-tail chain of APAn aided in stabilizing the ligand–receptor binding. In most cases, compounds with branched chains exhibited stronger affinity than APA6, indicating the beneficial impact of the introduction of methyl side chains, allowing them to effectively compete with ABA for receptor binding (Table 6). However, it is important to note that strong binding to the receptor does not necessarily imply high activity towards PP2C phosphatase, which may be critical for the ABA receptor agonists. For instance, despite APA18 having a lower affinity for the receptor compared with APA11, its binary complexes with the selected PYLs had greater influence on the activity of HAB1. This suggested a more significant influence on the conformation of the protein–protein binding interface between the binary complex and HAB1, thereby rendering it a more effective ABA receptor antagonist.

### 2.5. Molecular Docking

**Figure 4:**
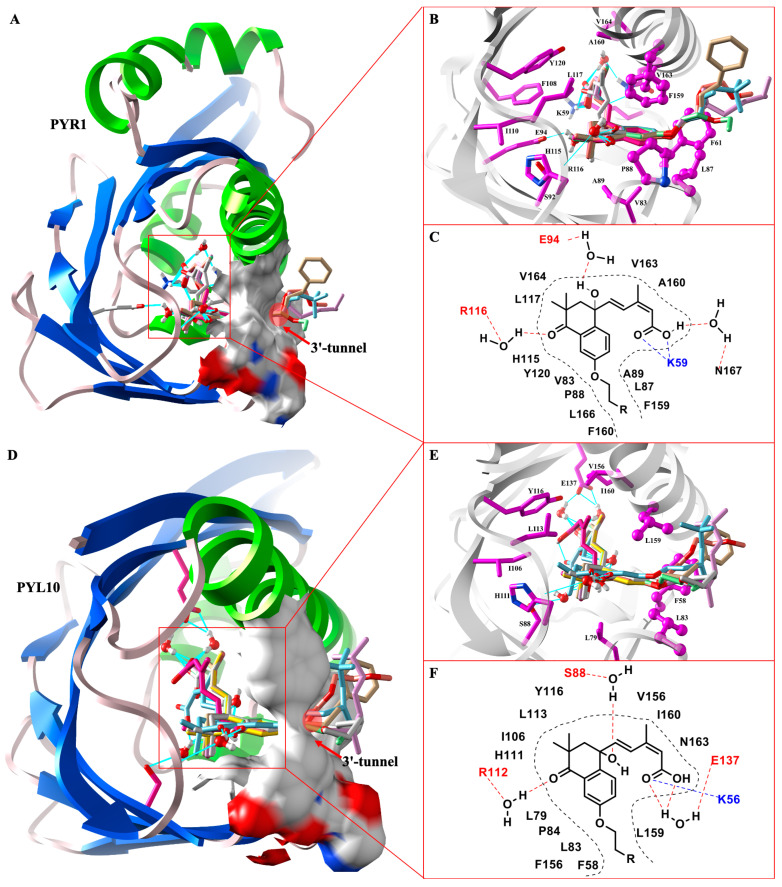
Analysis of docking-binding modes of compounds ABA, *iso*-PhABA, APAn with PYR1 (**A**–**C**) and PYL10 (**D**–**F**). Hydrophobic surfaces rendered in (**A**,**D**) formed by 3′-tunnel amino acid residues. Polar interactions are connected with cyan straight lines. The hydrophobic amino acid residues comprising the 3′-tunnel in (**B**,**E**) are highlighted with a ball-and-stick model.

In order to investigate the highly antagonistic activities of APAn from a molecular point of view, we used the molecular docking technique to perform docking experiments for APAn with dimer PYR1 as well as monomer PYL10, respectively. In relation to Surflex-Dock docking results, it is generally accepted that the ligand has a strong binding affinity for the receptor as long as the value of the total score is greater than 5 [40]. As shown in Table 7, ABA, *iso*-PhABA, APA6, APA11, APA14–18 had scoring values greater than 10 with PYR1 as well as PYL10, which further suggested that all of these compounds had strong binding affinity for the ABA receptors. Specifically, *iso*-PhABA as well as APA6, APA11, and APA14–18 had higher scoring values for PYR1 and PYL10 than ABA, which was also consistent with the results of the MST assay, i.e., *iso*-PhABA as well as APA6, APA11, and APA14–18 had lower binding constants *K*_d_ than ABA for both PYR1 and PYL10. In addition, the antagonists APA6 (PYR1: 16.31, PYL10: 12.6) and APA18 (PYR1: 16.46, PYL10: 14.55) had greater scoring values than *iso*-PhABA (PYR1: 15.74, PYL10: 11.48) for both PYR1 and PYL10, which may have been due to the fact that their 6′ position substituents were all straight alkane chains or alkoxyalkane chains, making it easier for them to pass through the narrow hydrophobic 3′-tunnel. Polar value describes the ability to form a hydrogen bond between the ligands and the receptors; APA6, APA11, APA14–18 all had significantly higher polar values than ABA, almost identical to *iso*-PhABA, suggesting that these antagonists were similar to *iso*-PhABA in forming polar interactions with the receptors. In order to further investigate the details of these antagonists’ binding to the receptors, we carefully analyzed the interactions of these antagonists in the binding pockets of PYR1 and PYL10. As shown in Figure 4A,D, the *iso*-PhABA skeleton structure of these antagonists was consistent with the ABA in the crystal structure, and the similarity between the docked conformation with the original ligand (ABA) of the recepectors was higher than 0.75. Whether binding to PYR1 or PYL10, when the 6′ alkane chain length of APAn was greater than three atoms, it could successfully pass through the hydrophobic 3′-tunnel to reach the interface where PYLs interacted with phosphatase, thereby blocking the transduction of the ABA signaling pathway in the plants. When the tail chain was less than three atoms, the conformational freedom of the tail chain was greatly reduced due to the constraints on conformation imposed by the hydrophobic amino acids in the 3′-tunnel. The docking program was able to predict the conformation of the tail chain in the 3′-tunnel very well, and the conformations between them had very good superposition. When the tail chain was larger than three atoms, the remaining part of the tail chain was exposed on the surface of the receptor and it could not be constrained by the amino acid residues of the acceptors, making the predicted conformation appear disordered. Analysis of the interaction of these antagonists with PYR1 showed that the carboxyl moiety of the antagonists formed a salt-bridge interaction with K59 and simultaneously formed a water molecule-mediated hydrogen bonding interaction with N167 (Figure 4B,C). The carbonyl oxygen atom and hydroxyl group of the head group formed water molecule-mediated hydrogen bond polar contacts with R116 and E94, respectively. The tail chain located in the 3′-tunnel formed strong hydrophobic interactions with the hydrophobic amino acid residues P88, L87, F61, F159, and V163; these hydrophobic interactions may be responsible for the antagonist’s higher binding affinity than *iso*-PhABA. The interaction between the antagonists and PYL10 (Figure 4E,F) was almost similar to that of PYR1. The carboxyl group formed a salt-bridge interaction with K56 and at the same time, it formed a water molecule-mediated hydrogen bond interaction with E137. The carbonyl oxygen atom and hydroxyl group of the head group formed water molecule-mediated hydrogen bond polar contacts with R112 and S88, respectively. Interestingly, the pore size of the 3′-tunnel of PYL10 seemed to be larger than that of PYR1, and the tail chain located in the 3′-tunnel of PYL10 formed hydrophobic interactions with the hydrophobic amino acid residues L83, F58, and L159.

### 2.6. Molecular Dynamics Simulation

**Figure 5:**
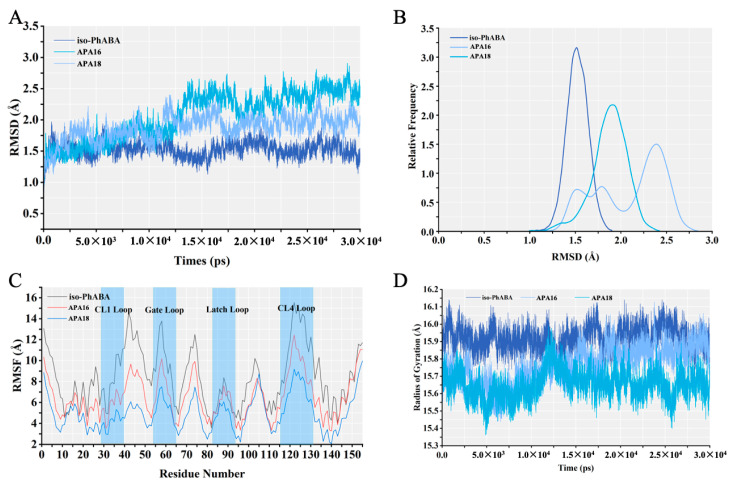
(**A**) RMSD of the backbone atoms of PYL10 during MD simulations; (**B**) frequency of distribution corresponding to RMSD; (**C**) variation of RMSF in the simulated system; (**D**) changes in radius of gyration in the simulated system.

To further demonstrate the reliability of the molecular docking results and the stability of the complexes, molecular dynamics simulations of the ligand–PYL10 system were performed. During molecular dynamics simulations, all systems were continuously optimized until equilibrium was reached, and analysis of the entire simulation process helped to provide a comprehensive understanding of the ligand–receptor interaction patterns. The equilibrium of the molecular dynamics simulations was judged according to the root mean square deviation (RMSD) values of the main chain carbon atoms. Figure 5A,B show the variations of RMSD with time for different systems. From Figure 5A, it can be found that the RMSD values of the APA16-PYL10 and APA18-PYL10 systems gradually increased throughout the simulation and tended to converge and equilibrate until 20 ns, indicating that all three systems had reached a steady state. The mean RMSD values of stabilized *iso*-PhABA-PYL10, APA16-PYL10, and APA18-PYL10 were 1.52 Å, 2.45 Å, and 1.97Å, respectively. Among them, the relative frequencies of distribution of RMSDs for APA16 and APA18 were greater than that for *iso*-PhABA, which may be due to the fact that they had long tail chains. Figure 5C shows the changes in RMSF for each system during the simulation. Overall, the volatility of amino acid residues was less than that of *iso*-PhABA in both the APA16-PYL10 and APA18-PYL10 systems, suggesting that these two compounds bind more stably to the receptor. Of the four free loops where the ligand enters the cavity, the CL1 and latch loops fluctuated less, suggested that they may have been involved in the interaction with the ligand. Further Rg analysis revealed (Figure 5D) that the Rg value of *iso*-PhABA was stable overall, whereas the Rgs of both APA16 and APA18 were initially lower than that of *iso*-PhABA and gradually approached that of *iso*-PhABA as the simulation time continued after 20 ns.

Based on the MMPBSA method in Amber14, we further collected 5000 conformations from the equilibrium trajectories to calculate the binding free energy for each system. This method allowed for the decomposition of the total energy into individual components, including the electrostatic energy (*E*_ele_), the van der Waals energy (*E*_VDW_), the energy of non-polar solvation (*E*_npolar_), and the energy of polar solvation (*E*_PB_), which helped us to further determine the interaction modes and the driving forces of ligand binding. The data in the Table 8 show that the binding free energies of all three systems were negative, indicating that these three ligands can form stable complexes with PYL10. Calculations of the decomposition of free energy revealed the binding affinities of APA16 and APA18 to PYL10, in which *E*_ele_ and *E*_VDW_ contributed much more to the free energy of both than the other subcomponents, suggesting that the formation of polar interactions and hydrophobic interactions between the ligands and PYL10 were the main reasons for their high receptor affinity. These results were similar to the molecular docking results. We also performed residue decomposition free energy calculations to determine the binding free energy. As shown in Figure S42, the hydrogen bonding interactions formed by the key polar residue K32 with the carboxyl group of the ligand became stronger with the introduction of the tail chain. Amino acid residues within the 3′-tunnel were also able to form stronger hydrophobic interactions with APA16 and APA18 than *iso*-PhABA. For example, the tail chain of APA16 formed strong hydrophobic contacts with F34 (−1.86 kcal/mol), L59 (−1.27 kcal/mol), P60 (−0.31 kcal/mol), F131 (−1.70 kcal/mol), and L135 (−3.71 kcal/mol). Notably, the value of the energy contribution provided by the polar interactions formed between residues K32 and R51 and APA18 was much higher than that of *iso*-PhABA and APA16. This result further explained the high antagonistic activity in seed germination and the high receptor-binding affinity of APA18. It was also demonstrated that a change in the structure of the substituent group at the 6′ position affected the ligands’ binding to the residues in the 3′-tunnel, thus affecting the binding affinities of the ligands to the receptors.

## 3. Materials and Methods

Equipment and Materials. All chemicals and reagents utilized in this study were purchased from reputable sources. Melting points were recorded using a Cole–Parmer (Vernon Hills, IL, USA) microscope melting-point apparatus and were left uncorrected. The ^1^H NMR (500 MHz) and ^13^C NMR (125 MHz) spectra were acquired on a Bruker (Billerica, MA, USA) Avance III 500 spectrometer, with tetramethylsilane serving as the internal standard. High-resolution mass spectrometry data were obtained using an Agilent (Santa Clara, CA, USA)-ESI thermography. Thermal images were analyzed using VarioCAM (Dresden, Germany) HD thermography. The receptor proteins and HAB1 phosphatase were expressed and purified following our previously described standard protocols. The MST experiment was conducted using a NanoTemper (Munich, Germany) Monolith NT.115.

Synthetic Procedures. General synthetic procedure of intermediate IV. In a 500 mL three-necked flask, 200 mL of toluene was combined with 91.6 mmol of compound III and 274.8 mmol of anhydrous aluminum trichloride. The resulting mixture was heated to reflux for 3 h under an argon atmosphere, and then cooled to room temperature and carefully quenched with ice water. The mixture was then extracted with ethyl acetate (100 mL × 3), and the combined organic layer was washed with brine, dried over anhydrous sodium sulfate, and filtered. The filtrate was concentrated in vacuo to yield the crude product, which was further pulped with 200 mL of a petroleum ether/ethyl acetate solution (*V*/*V* = 10:1) overnight at room temperature. The resulting mixture was filtered, washed with a small amount of solvent, and dried to afford compound IV.

General synthetic procedure of intermediate V. In a 50 mL round-bottom flask, 24.48 mmol of compound IV, 39.17 mmol of potassium carbonate, and 36.72 mmol of bromo- or iodo-substituted alkanes or *p*-toluenesulfonate were dissolved in 20 mL of acetonitrile. The reaction mixture was refluxed for 12 h under stirring and then cooled to room temperature. It was filtered, and the filtrate was concentrated in vacuo. The residue was purified using silica gel column chromatography (PE:EtOAc = 30:1, *V*/*V*) to afford compound V.

General synthetic procedure of intermediate VI. A 200 mL solution of 33.29 mmol of (*Z*)-3-methylpent-2-en-4-yn-1-ol in dried tetrahydrofuran (THF) was cooled to −78 °C under an argon atmosphere. Then, 67.25 mmol of *n*-butyl lithium (2.4 M in hexane) was added slowly using a syringe. The resulting mixture was stirred at −78 °C for 30 min, and a solution containing 22.19 mmol of compound V dissolved in 50 mL of dried THF was then added. The mixture was stirred for 30 min at −78 °C and for an additional 3 h at room temperature. After quenching the reaction with water, the mixture was washed with brine and extracted with ethyl acetate (100 mL × 3). The combined organic phase was dried over anhydrous sodium sulfate and filtered. The evaporation of solvent yielded a yellow oil, which was purified through silica gel column chromatography (PE:EtOAc = 2:1, *V*/*V*) to afford compound VI.

General synthetic procedure of intermediate VII. A solution of intermediate VI (0.30 mmol) in 50 mL of dried THF was stirred and cooled to 0 °C. The addition of Red-Al (0.9 mmol) was then carried out dropwise using a syringe. The resulting mixture was stirred at room temperature for 2 h. Subsequently, the reaction was quenched by slowly adding 40 mL of ice water. The mixture was extracted with ethyl acetate (30 mL × 3). The combined organic phase was then washed with 30 mL of brine, dried over anhydrous sodium sulfate, and concentrated in vacuo. The residue was further purified through silica gel column chromatography (PE:EtOAc = 1:1, *V*/*V*) to afford compound VII.

General synthetic procedure of intermediate VIII. A solution of intermediate VII (4.63 mmol) and Dess–Martin periodinane (DMP) (9.72 mmol) was prepared via dissolving them in 20 mL of dichloromethane (DCM). The resulting mixture was stirred at room temperature for 30 min. Afterward, 20 mL of aqueous Na_2_S_2_O_3_ solution and 20 mL of aqueous sodium bicarbonate solution were added to the mixture. The resulting mixture was stirred for an additional 30 min and then extracted with DCM (30 mL × 3). The combined organic phase was washed with an aqueous brine solution, dried using anhydrous sodium sulfate, and concentrated under vacuum. This process yielded a yellow oily compound VIII, which was used directly in the subsequent reaction without further purification.

General synthetic procedure of APAn compound. A solution of 4.63 mmol of intermediate VIII in 20 mL of solvent (*t*-BuOH/H_2_O = 3:1, *V*/*V*) was prepared. Into this solution, 46.3 mmol of NaClO_2_, 92.60 mmol of 2-methyl-2-butene, and 18.52 mmol of NaH_2_PO_4_·2H_2_O were added and stirred at room temperature for 30 min. The resulting mixture was then extracted with EtOAc (20 mL × 3). The combined organic layer was washed with brine, dried over anhydrous sodium sulfate, and concentrated under vacuum. The residue was purified using silica gel column chromatography (PE:EtOAc = 1:1, *V*/*V*) to afford the compound APAn. The characterization data for all intermediates and target compounds are provided in the Supporting Information.

Bioassays. Seed germination promotion assay—*A. thaliana* (Columbia, WT) [29]. *A. thaliana* seeds were stratified for 3 days before being sown on MS solid media supplemented with 1% sucrose. The samples were divided into three different treatments: 5 μM APAn alone, 5 μM APAn with 1 μM ABA, and 5 μM APAn with 10 mM NaCl. Each plate consisted of 50 seeds, and three plates were prepared for each treatment. The plates were placed in a controlled phytotron environment at a temperature of 22 °C, with a light–dark cycle of 16 h and 8 h. After 48 h, the germination rates of the seeds were determined through calculating the average germination rates based on the three replicates.

Colza (Zhongzheyou 28) [41,42]: Colza seeds that were uniform and plump in size were subjected to sterilization by soaking them in 70% ethanol for 10 min, followed by rinsing with distilled water for 6–7 times. After removing excess water, 50 colza seeds were placed on a plate lined with two sheets of filter paper. Each plate was then treated with 5 mL of the test agent. The plates were transferred to a controlled phytotron environment with a temperature of 24 °C, with a light–dark cycle of 16 h and 8 h. After 48 h, the germination rate was determined. Additionally, the lengths of the cotyledons and roots of the seedlings were measured after 4 days. Each experiment was repeated three times.

Rice (Nipponbare) [43,44]: The rice seeds utilized in this study were selected to ensure uniformity and plumpness. Prior to experimentation, they were subjected to a sterilization process involving a 10 min immersion in 70% ethanol, followed by rinsing with distilled water 6–7 times. Excess water was then removed, and 50 rice seeds were evenly distributed on a plate lined with two sheets of filter paper. Each plate was subsequently treated with 5 mL of the test agent. To provide an optimal growth environment, the plates were transferred to a phytotron under controlled conditions, including temperature set to 24 °C and a light–dark cycle of 16 h and 8 h. Observations were recorded daily to determine the number of germinated seeds, and after a period of 7 days, the length of the seedlings’ cotyledons and roots were measured. The germination potential, germination rate, germination index, and vitality index were calculated using Equations (1) to (4), respectively:

Germination potential (%) = (number of germinated seeds by day 3/total number of test seeds) × 100(1)

Germination rate (%) = (number of germinated seeds by day 7/total number of test seeds) × 100%(2)

Germination index = ∑ (*Gi*/*Ti*) (3)

where *Gi* is the number of germinated seeds by the *i*th day, and *Ti* is the day of the germination test.

Vitality index = germination index × cotyledon length(4)

The experiments were repeated three times in each group.

Wheat (Luyuan 502) [45]: In this study, uniform plump wheat seeds were subjected to a sterilization process involving a 10 min immersion in 70% ethanol followed by rinsing with distilled water 6–7 times. Excess water was then removed, and 50 wheat seeds were placed on a plate with two sheets of filter paper. Subsequently, 5 mL of the designated test agent was added to each plate. The plates were then transferred to a phytotron under controlled conditions including a temperature of 24 °C and a light–dark cycle of 16 h and 8 h. After 48 h of germination under normal temperature, the germination rate was calculated, and the lengths of the seedlings’ cotyledons and roots were determined. Furthermore, to evaluate the impact of low-temperature stress, the germination rate was calculated after 5 days, and the lengths of the seedlings’ cotyledons and roots were determined after 14 days of germination at 4 °C.

Stomatal movement assay [46]. *A. thaliana* seeds (Columbia, WT) were subjected to a sterilization process involving a 10 min immersion in 70% ethanol. Subsequently, they were rinsed with distilled water 6–7 times and excess water was carefully removed. These sterilized seeds were then placed in a Petri dish containing 1/2 MS solid culture medium. To promote vernalization, the dish was sealed with a split film and stored in a refrigerator at 4 °C for a period of 3 days. After the vernalization period, the Petri dish was transferred to an incubator and maintained under controlled conditions at a temperature of 25 °C, under a light–dark cycle of 16 h and 8 h, for cultivation for 5 days. Following this, the 5-day-old seedlings were transplanted into sterilized nutrient soil and placed in a greenhouse for 2 weeks to further their growth. On the 15th day, each pot containing *A. thaliana* seedlings was subjected to a spray application of a 3 mL aqueous solution. The solution contained either 5 μM ABA, *iso*-PhABA, or a combination of 5 μM ABA with 25 μM APAn. After a 24 h incubation period, the surface temperature of the leaves was recorded using an infrared thermal imager. The experiment was repeated three times for each group.

Microscale thermophoresis (MST) assay [25]. The ABA receptors PYR1, PYL2, PYL3, PYL6, and PYL10 were labeled with the red fluorescent dye NT-647-NHS. A Monolith NT Protein Labeling Kit was used, following the provided instructions. The tested compounds were dissolved in DMSO, and a dilution series of the compounds (16 concentrations ranging from 0.015 to 1000 μM) was prepared using PBS buffer, mixed with the labeled PYLs, and then transferred into Monolith NT.115 capillaries. Measurements were conducted on a NanoTemper Monolith NT.115 instrument using 40% LED power and 60% MST power. Three independent measurements were performed for each compound. The obtained data were analyzed and the dissociation constant *K*_d_ was calculated based on the results of three individual experiments, using MO. Affinity analysis software version 2.3.

HAB1 phosphatase activity assay [25]. The phosphatase activity assay of HAB1 was conducted using a Serine/Threonine Phosphatase Assay System kit from Promega. A solution of 3 μM PYLs and 1 μM HAB1 was prepared in Tris buffer and incubated at room temperature. Following this, 50 μL of molybdate pigment/additive was added to stop the reaction. After a 30 min incubation period, the absorbance of the system at 630 nm was measured using a microplate reader. The phosphatase activity of HAB1 was calculated, and this process was repeated three times for each treatment.

Molecular Docking [47]. The Surflex-Dock program, integrated with Sybyl, was utilized to dock the synthesized APAn with the ABA receptors PYR1 (PDB ID: 3K3K10 [48]) and PYL10 (PDB ID: 3R6P11 [49]) individually. Surflex-Dock employs an automated docking procedure to investigate the recognition process between ligands and receptors and predict their affinity. Prior to docking, the protein models underwent pretreatment, which involved retaining key water molecules and adding polar hydrogen atoms and charges. For both PYR1 and PYL10, a ligand-based docking mode was employed to generate the protomol. As the ligand structures were flexible, the Surflex-Dock program generated one hundred docking conformations for each ligand, from which the most likely bioactivity conformation was selected as the final result. The images were visualized using UCSF Chimera 1.17.3 software.

Molecular Dynamics Simulation [27]. Molecular dynamics simulation (MDS) can indicate the dynamic behaviors of ligands and the changes of protein structures. Based on this, the docked complexes of ABA receptor (PYR10) with *iso*-PhABA, APA16, and APA18 were selected, and compared with the respective bioassays. First, to ensure that the docked complexes had enough space for conformational adjustment, we pre-treated the complexes by dissolving them into TIP3P water with a boundary distance of 10 Å. Secondly, Na^+^ was added to keep the simulated system electrically neutral. Force-field parameters for the proteins were created with the Amber ff03 force field and the compounds were processed using the antechamber program by selecting the general amber force field (GAFF). Thirdly, each system was optimized according to the steepest descent in 2000 steps to gradually minimize the energy, and conjugate gradient optimization via 2000 steps was performed to eliminate poor contact with the composite structure. Finally, each system was gradually heated from 10 to 298 K in 7 steps. The whole system was subjected to molecular dynamics calculation at 30 ns at thermodynamic temperature (T = 298 K, 101 KPa) and the coordinate trajectories were saved every 1 ps. All simulations were performed using the Sander program in Amber 14.

In addition, molecular mechanics Poisson−Boltzmann surface area (MMPBSA) was used in the analysis of the post-processing trajectory. The last 2 ns of equilibrated MD trajectory were extracted to calculate the binding free energy, using the MMPBSA.py module in Amber 14.

## 4. Conclusions

In summary, we synthesized 12 new refined compounds with branched or oxygen-substituted tail chains on the 6′ position of APAn, based on previous reports. The aim was to develop ABA receptor antagonists with increased activity and gain insights into the 3′-tunnel. The results demonstrated that introducing a branched chain on the shorter tail chain could convert agonistic activity to antagonistic. This indicated that changes inside the 3′-tunnel could impact the conformation of the protein–protein binding surface with PP2C phosphatase. However, as the tail chain increased, the introduction of branched chains caused irregular changes in activity. This finding suggested that the position of the branched chain systematically influenced the conformation, which differed from straight chains. Additionally, the introduction of oxygen atoms had a noticeable impact on activity, possibly due to their interaction with polar amino acid residues in the tunnel. This interaction aligns with the previous literature suggesting that tail chains might not be optimal for channel groups. Molecular docking and molecular dynamics simulations provided insights into the role of these tail chains in the 3′-tunnel. Fortunately, this study yielded several excellent ABA receptor antagonists, such as APA18, which not only antagonized the negative effects mediated by ABA but also exhibited synergistic effects when combined with other drugs that antagonized ABA function, such as fluridone and gibberellin. This finding is crucial for improving crop yields under adverse conditions like cold and salinity stress. Moreover, the use of APA18 offers opportunities for further research into ABA metabolism in plants.

## Figures and Tables

**Table 1:** The effect of agent treatment on *A. thaliana* seed germination (48 h). Values represent means ± SD, and those marked with the same letter are statistically significant (*p* < 0.05).

Treatment 1 *^a^*	Germination Rate (%)	Treatment 2 *^b^*	Germination Rate (%)	Treatment 3 *^c^*	Germination Rate (%)
DMSO	91.33 ± 2.31 ^A^	DMSO	92.67 ± 2.31 ^A^	DMSO	93.33 ± 3.06 ^A^
ABA	13.33 ± 1.15 ^G^	ABA	31.33 ± 3.06 ^F^	ABA	10.67 ± 3.06 ^G^
*iso*-PhABA	8.67 ± 3.06 ^G^	*iso*-PhABA	22.67 ± 1.15 ^GH^	*iso*-PhABA	7.33 ± 2.31 ^G^
/	/	/	/	NaCl	48.67 ± 6.11 ^E^
APA6	85.33 ± 3.06 ^A^	ABA + APA6	78.67 ± 3.06 ^B^	APA6 + NaCl	82.67 ± 3.06 ^B^
APA11	84.67 ± 4.16 ^A^	ABA + APA11	75.33 ± 6.11 ^B^	APA11 + NaCl	81.33 ± 4.16 ^B^
APA14	85.33 ± 4.16 ^A^	ABA + APA14	78.67 ± 4.16 ^B^	APA14 + NaCl	86.00 ± 2.00 ^B^
APA15	85.33 ± 3.06 ^A^	ABA + APA15	74.67 ± 1.15 ^B^	APA15 + NaCl	85.33 ± 1.15 ^B^
APA16	84.67 ± 3.06 ^A^	ABA + APA16	76.67 ± 1.15 ^B^	APA16 + NaCl	84.00 ± 4.00 ^B^
APA17	83.33 ± 3.06 ^A^	ABA + APA17	75.33 ± 3.06 ^B^	APA17 + NaCl	82.67 ± 3.06 ^B^
APA18	83.33 ± 2.31 ^A^	ABA + APA18	76.00 ± 2.00 ^B^	APA18 + NaCl	84.00 ± 3.46 ^B^

*^a^ A. thaliana* seeds were treated with 5 μM corresponding compound APAn alone, and 5 μM ABA or 5 μM *iso*-PhABA treatments were used in the control groups. *^b^ A. thaliana* seeds were treated with a mixture of 1 μM ABA and 5 μM APAn, and 1 μM ABA or 1 μM *iso*-PhABA treatments were used in the control groups. *^c^ A. thaliana* seeds were treated with the combination of 10 mM NaCl and 5 μM APAn, and 5 μM ABA or 5 μM *iso*-PhABA treatments were used in the control groups.

**Table 2:** The effect of agent treatment on colza seed germination (48 h). Values represent means ± SD, and those marked with the same letter are statistically significant (*p* < 0.05).

Treatment 1 *^a^*	Germination Rate (%)	Treatment 2 *^b^*	Germination Rate (%)	Treatment 3 *^c^*	Germination Rate (%)
DMSO	91.77 ± 2.75 ^A^	DMSO	89.52 ± 3.30 ^A^	DMSO	91.43 ± 5.71 ^A^
ABA	18.70 ± 3.92 ^K^	ABA	49.52 ± 3.30 ^K^	ABA	2.86 ± 2.86 ^K^
*iso*-PhABA	13.60 ± 3.29 ^K^	*iso*-PhABA	21.90 ± 4.36 ^K^	*iso*-PhABA	0 ^K^
/	/	/	/	NaCl	42.86 ± 1.00 ^H^
APA6	85.33 ± 3.65 ^ABC^	ABA + APA6	82.86 ± 2.86 ^ABC^	APA6 + NaCl	77.14 ± 2.86 ^BCD^
APA11	87.13 ± 1.15 ^AB^	ABA + APA11	80.95 ± 3.30 ^AB^	APA11 + NaCl	77.14 ± 4.95 ^BCD^
APA14	86.20 ± 4.56 ^AB^	ABA + APA14	75.24 ± 3.30 ^AB^	APA14 + NaCl	75.24 ± 6.00 ^CD^
APA15	83.93 ± 1.79 ^BCD^	ABA + APA15	75.24 ± 4.36 ^BCD^	APA15 + NaCl	73.33 ± 3.30 ^CDE^
APA16	84.77 ± 4.38 ^ABCD^	ABA + APA16	76.19 ± 4.36 ^ABCD^	APA16 + NaCl	80.95 ± 4.36 ^BC^
APA17	83.33 ± 2.97 ^BCD^	ABA + APA17	83.81 ± 4.36 ^BCD^	APA17 + NaCl	85.71 ± 7.56 ^AB^
APA18	83.83 ± 4.38 ^BCD^	ABA + APA18	81.90 ± 5.95 ^BCD^	APA18 + NaCl	82.86 ± 4.95 ^ABC^

*^a^* Colza seeds treated with 10 μM corresponding APAn compound alone. *^b^* Colza seeds treated with both 1 μM ABA and 10 μM corresponding APAn compound; 1 μM ABA and 1 μM *iso*-PhABA treatment for control groups. *^c^* Colza seeds treated both with 25 mM NaCl and 25 μM corresponding APAn compound; 25 μM ABA and 25 μM *iso*-PhABA treatment for control groups.

**Table 3:** The germination parameters of rice seeds under different treatments. Values represent means ± SD, and those marked with the same letter are statistically significant (*p* < 0.05).

Treatment		Germination Potential (%)	Germination Rate (%)	Germination Index	Cotyledon Length (cm)	Root Length (cm)	Vitality Index
Compd.	Additive						
DMSO	none	77.33 ± 3.06 ^A^	97.33 ± 3.06 ^AB^	15.34 ± 0.44 ^ABC^	6.19 ± 0.67 ^A^	8.08 ± 0.57 ^A^	94.93 ± 10.13 ^A^
10 μM ABA	none	36.00 ± 4.00 ^G^	97.33 ± 2.31 ^AB^	13.50 ± 0.36 ^E^	4.42 ± 0.40 ^F^	4.93 ± 0.67 ^G^	63.07 ± 5.71 ^DE^
10 μM *iso*-PhABA	none	18.00 ± 2.00 ^H^	98.00 ± 1.46 ^AB^	12.92 ± 0.30 ^F^	3.76 ± 0.51 ^G^	4.51 ± 0.42 ^G^	48.57 ± 6.38 ^E^
10 μM APA6	none	73.33 ± 2.31 ^ABCD^	97.33 ± 1.15 ^AB^	15.17 ± 0.26 ^ABC^	5.81 ± 0.46 ^ABC^	6.99 ± 0.52 ^BC^	88.16 ± 6.89 ^ABC^
10 μM APA11	none	73.33 ± 3.06 ^ABCD^	96.67 ± 2.31 ^AB^	15.12 ± 0.19 ^ABCD^	5.63 ± 0.35 ^BCD^	7.00 ± 0.81 ^BC^	85.14 ± 5.18 ^ABC^
10 μM APA14	none	77.33 ± 2.31 ^A^	94.00 ± 2.00 ^B^	14.94 ± 0.24 ^ABCD^	5.75 ± 0.49 ^ABC^	6.98 ± 0.71 ^BC^	85.91 ± 7.13 ^ABC^
10 μM APA15	none	74.67 ± 4.62 ^ABCD^	98.67 ± 1.15 ^A^	15.41 ± 0.37 ^A^	5.73 ± 0.51 ^BC^	7.17 ± 0.40 ^B^	88.30 ± 7.84 ^ABC^
10 μM APA16	none	76.67 ± 6.11 ^AB^	95.33 ± 3.06 ^AB^	15.36 ± 0.64 ^AB^	5.62 ± 0.39 ^BCD^	7.07 ± 0.56 ^BC^	86.34 ± 6.55 ^ABC^
10 μM APA17	none	75.33 ± 4.16 ^ABC^	96.67 ± 1.15 ^A^	15.42 ± 0.36 ^A^	5.78 ± 0.60 ^ABC^	7.12 ± 0.46 ^BC^	89.13 ± 9.09 ^AB^
10 μM APA18	none	76.67 ± 3.06 ^AB^	96.67 ± 1.15 ^AB^	15.26 ± 0.24 ^ABC^	5.87 ± 0.36 ^AB^	7.10 ± 0.67 ^BC^	89.58 ± 5.46 ^AB^
DMSO	none	77.33 ± 3.06 ^A^	97.33 ± 3.06 ^AB^	15.34 ± 0.44 ^A^	6.19 ± 0.67 ^A^	8.08 ± 0.57 ^A^	94.93 ± 10.13 ^A^
1 μM ABA	none	51.33 ± 2.31 ^DE^	96.67 ± 3.06 ^AB^	14.27 ± 0.37 ^BCDEF^	5.03 ± 0.23 ^EF^	6.27 ± 0.42 ^EF^	67.91 ± 3.30 ^D^
1 μM *iso*-PhABA	none	26.67 ± 8.33 ^G^	97.33 ± 2.31 ^AB^	13.33 ± 0.71 ^H^	4.86 ± 0.47 ^F^	5.43 ± 0.35 ^H^	64.77 ± 6.75 ^E^
10 μM APA6	1 μM ABA	62.00 ± 2.00 ^BC^	96.67 ± 2.31 ^A^	14.88 ± 0.27 ^AB^	5.93 ± 0.32 ^ABC^	7.01 ± 0.50 ^BC^	88.26 ± 4.79 ^AB^
10 μM APA11	1 μM ABA	60.67 ± 3.06 ^BC^	95.33 ± 3.06 ^AB^	14.69 ± 0.50 ^ABCD^	5.91 ± 0.32 ^ABC^	6.97 ± 0.51 ^BC^	86.86 ± 5.18 ^ABC^
10 μM APA14	1 μM ABA	62.00 ± 6.00 ^BC^	97.33 ± 1.15 ^AB^	14.73 ± 0.36 ^ABCD^	6.02 ± 0.63 ^AB^	7.27 ± 0.67 ^B^	88.67 ± 9.09 ^AB^
10 μM APA15	1 μM ABA	60.00 ± 5.29 ^BC^	98.00 ± 0.00 ^AB^	14.72 ± 0.25 ^ABCD^	6.01 ± 0.81 ^AB^	7.25 ± 0.46 ^B^	88.45 ± 11.55 ^AB^
10 μM APA16	1 μM ABA	59.33 ± 1.15 ^BC^	98.00 ± 0.00 ^AB^	14.69 ± 0.05 ^ABCD^	6.11 ± 0.69 ^AB^	7.06 ± 0.51 ^BC^	89.76 ± 9.74 ^AB^
10 μM APA17	1 μM ABA	64.00 ± 4.00 ^B^	97.33 ± 1.15 ^AB^	14.83 ± 0.22 ^AB^	6.06 ± 0.55 ^AB^	7.16 ± 0.68 ^B^	89.89 ± 8.01 ^AB^
10 μM APA18	1 μM ABA	62.67 ± 4.62 ^BC^	97.33 ± 1.15 ^AB^	14.78 ± 0.29 ^ABC^	6.12 ± 0.46 ^AB^	7.08 ± 0.45 ^BC^	90.43 ± 6.78 ^AB^
DMSO	none	77.33 ± 3.06 ^A^	97.33 ± 3.06 ^A^	15.34 ± 0.44 ^A^	6.19 ± 0.67 ^A^	8.08 ± 0.57 ^A^	94.93 ± 10.13 ^A^
50 μM ABA	none	0 ^I^	97.33 ± 2.31 ^A^	11.28 ± 0.33 ^G^	4.10 ± 0.77 ^I^	4.48 ± 0.77 ^J^	46.26 ± 8.49 ^H^
50 μM *iso*-PhABA	none	0 ^I^	49.33 ± 3.06 ^B^	5.42 ± 0.33 ^H^	3.41 ± 0.84 ^J^	2.58 ± 0.39 ^K^	18.47 ± 4.47 ^I^
none	100 mM NaCl	46.67 ± 1.15 ^B^	96.00 ± 0.00 ^A^	13.94 ± 0.05 ^AB^	4.93 ± 0.51 ^GH^	5.71 ± 0.85 ^FGHI^	68.76 ± 6.81 ^FG^
50 μM APA6	100 mM NaCl	64.00 ± 4.00 ^BC^	95.33 ± 1.15 ^A^	14.55 ± 0.31 ^BCD^	6.11 ± 0.70 ^ABC^	7.37 ± 0.61 ^AB^	88.90 ± 9.89 ^ABCD^
50 μM APA11	100 mM NaCl	61.33 ± 1.15 ^CD^	96.00 ± 2.00 ^A^	14.51 ± 0.29 ^BCDE^	6.12 ± 0.39 ^ABC^	7.13 ± 0.92 ^BCD^	88.76 ± 5.67 ^ABCD^
50 μM APA14	100 mM NaCl	64.00 ± 4.00 ^DE^	97.33 ± 1.15 ^A^	14.82 ± 0.06 ^BCDE^	6.12 ± 0.32 ^ABC^	7.34 ± 0.58 ^AB^	90.68 ± 4.57 ^ABC^
50 μM APA15	100 mM NaCl	60.00 ± 4.00 ^B^	96.67 ± 1.15 ^A^	14.55 ± 0.06 ^ABC^	6.16 ± 0.28 ^AB^	7.42 ± 0.65 ^AB^	89.63 ± 3.94 ^ABCD^
50 μM APA16	100 mM NaCl	60.67 ± 3.06 ^BC^	96.00 ± 4.00 ^A^	14.51 ± 0.44 ^BCDE^	6.06 ± 0.34 ^ABCD^	7.46 ± 1.03 ^AB^	87.95 ± 5.29 ^ABCDE^
50 μM APA17	100 mM NaCl	61.33 ± 1.15 ^B^	98.00 ± 2.00 ^A^	14.72 ± 0.30 ^BCDE^	6.15 ± 0.44 ^AB^	7.19 ± 0.84 ^BCD^	90.53 ± 6.41 ^ABC^
50 μM APA18	100 mM NaCl	66.00 ± 3.46 ^B^	96.67 ± 1.15 ^A^	14.83 ± 0.58 ^ABC^	6.13 ± 0.17 ^AB^	7.18 ± 0.97 ^BCD^	90.95 ± 3.82 ^AB^

**Table 4:** The effect of agent treatment on wheat seed germination. Values represent means ± SD, and those marked with the same letter are statistically significant (*p* < 0.05).

Culture Temperature	Germination Rate (%)		Cotyledon Length (cm)		Root Length (cm)	
	24 °C *^a^*	4 °C *^b^*	24 °C *^a^*	4 °C *^b^*	24 °C *^a^*	4 °C *^b^*
DMSO	93.67 ± 2.35 ^ABC^	78.50 ± 0.75 ^C^	1.38 ± 0.11 ^B^	2.38 ± 0.11 ^DE^	3.58 ± 0.35 ^A^	4.08 ± 0.46 ^CD^
FD	95.26 ± 2.41 ^AB^	90.27 ± 3.94 ^AB^	1.32 ± 0.19 ^B^	2.52 ± 0.16 ^D^	3.40 ± 0.25 ^A^	4.52 ± 0.53 ^BC^
DCN	84.73 ± 2.42 ^E^	63.21 ± 5.02 ^D^	0.80 ± 0.07 ^C^	1.70 ± 0.10 ^G^	1.84 ± 0.15 ^C^	3.62 ± 0.31 ^D^
PBZ	87.52 ± 1.70 ^DE^	72.40 ± 1.15 ^C^	0.62 ± 0.16 ^D^	1.88 ± 0.19 ^G^	1.54 ± 0.21 ^C^	3.82 ± 0.36 ^D^
GA_3_	94.69 ± 0.34 ^AB^	87.40 ± 2.82 ^B^	1.42 ± 0.13 ^B^	2.72 ± 0.08 ^C^	3.62 ± 0.43 ^A^	4.62 ± 0.26 ^AB^
APA18	96.22 ± 1.70 ^A^	91.09 ± 6.92 ^AB^	1.46 ± 0.09 ^AB^	2.76 ± 0.13 ^BC^	3.56 ± 0.42 ^A^	4.72 ± 0.41 ^AB^
FD + APA18	96.47 ± 3.23 ^CD^	93.95 ± 1.93 ^A^	1.48 ± 0.13 ^AB^	2.94 ± 0.21 ^AB^	3.76 ± 0.24 ^A^	4.96 ± 0.44 ^AB^
DCN + APA18	91.79 ± 1.64 ^BC^	72.87 ± 0.65 ^C^	0.88 ± 0.08 ^C^	2.10 ± 0.16 ^F^	1.90 ± 0.37 ^C^	3.78 ± 0.34 ^D^
PBZ + APA18	91.44 ± 2.68 ^BC^	76.39 ± 3.26 ^C^	0.94 ± 0.11 ^C^	2.24 ± 0.11 ^EF^	2.54 ± 0.18 ^B^	3.98 ± 0.26 ^D^
GA_3_ + APA18	97.49 ± 1.10 ^A^	94.45 ± 0.90 ^A^	1.62 ± 0.13 ^A^	3.00 ± 0.16 ^A^	3.64 ± 0.09 ^A^	5.08 ± 0.46 ^A^

*^a^* Wheat seed germination rate in the presence of 20 μM fluridone (FD), diniconazole (DCN), paclobutrazol (PBZ), gibberellic acid 3 (GA_3_) and 20 μM APA18 at 24 °C; cotyledons and roots of wheat seedlings were measured after 2 days of germination. *^b^* Wheat seed germination rate in the presence of 20 μM fluridone (FD), diniconazole (DCN), paclobutrazol (PBZ), gibberellic acid 3 (GA_3_) and 20 μM APA18 at 4 °C; cotyledons and roots of wheat seedlings were measured after 14 days of germination under 4 °C low-temperature stress.

**Table 5:** Binding affinity of selected APAn to PYLs (*K*_d_, μM). Values represent means ± SD, and those marked with the same letter are statistically significant (*p* < 0.05).

Ligand	PYR1	PYL2	PYL3	PYL6	PYL10
ABA	187.98 ± 28.99 ^A^	16.20 ± 4.84 ^A^	37.89 ± 2.41 ^A^	19.51 ± 3.92 ^A^	152.16 ± 31.03 ^A^
*iso*-PhABA	186.58 ± 22.30 ^A^	7.54 ± 2.36 ^B^	16.31 ± 2.78 ^B^	6.77 ± 0.32 ^C^	92.04 ± 5.13 ^B^
APA6	120.88 ± 13.65 ^B^	2.91 ± 0.17 ^CD^	4.09 ± 0.65 ^E^	1.62 ± 0.17 ^DE^	44.91 ± 8.09 ^D^
APA11	59.44 ± 3.41 ^CD^	0.46 ± 0.09 ^D^	3.11 ± 0.40 ^E^	0.90 ± 0.22 ^E^	17.60 ± 2.11 ^E^
APA14	60.84 ± 9.02 ^CD^	0.11 ± 0.01 ^D^	5.18 ± 0.84 ^DE^	2.55 ± 0.46 ^DE^	45.46 ± 2.47 ^D^
APA15	37.91 ± 3.09 ^D^	2.04 ± 0.29 ^D^	7.95 ± 1.66 ^CD^	1.99 ± 0.32 ^DE^	36.33 ± 2.18 ^DE^
APA16	53.43 ± 4.71 ^CD^	5.84 ± 0.24 ^BC^	7.84 ± 0.95 ^CD^	2.10 ± 0.31 ^DE^	65.01 ± 2.76 ^C^
APA17	42.20 ± 6.71 ^D^	7.07 ± 0.21 ^B^	18.99 ± 2.10 ^B^	13.12 ± 1.46 ^B^	23.92 ± 2.68 ^E^
APA18	73.94 ± 5.28 ^C^	0.14 ± 0.02 ^D^	9.33 ± 1.39 ^C^	4.17 ± 0.93 ^D^	37.48 ± 2.83 ^DE^

**Table 6:** Relative activity of HAB1 under action of ligand–receptor binary complex (%) *^a^*^,*b*^. Values represent means ± SD, and those marked with the same letter are statistically significant (*p* < 0.05).

Ligand	PYR1	PYL2	PYL3	PYL6	PYL10
ABA	0.64 ± 0.04 ^C^	2.15 ± 0.20 ^F^	2.32 ± 1.18 ^G^	3.08 ± 0.27 ^E^	0.99 ± 0.08 ^E^
*iso*-PhABA	0.51 ± 0.08 ^C^	3.28 ± 0.59 ^F^	2.44 ± 0.76 ^G^	1.39 ± 0.71 ^E^	1.80 ± 0.73 ^E^
APA6	85.62 ± 0.76 ^B^	95.48 ± 0.59 ^B^	82.93 ± 0.42 ^F^	86.9 ± 1.33 ^C^	88.50 ± 1.11 ^BC^
APA11	85.11 ± 0.44 ^B^	97.97 ± 0.78 ^A^	90.98 ± 0.56 ^C^	92.60 ± 0.46 ^B^	83.06 ± 0.36 ^D^
APA14	88.24 ± 0.44 ^B^	84.41 ± 0.78 ^E^	88.54 ± 0.73 ^D^	83.79 ± 1.07 ^CD^	90.46 ± 0.23 ^B^
APA15	91.35 ± 3.64 ^A^	87.63 ± 0.20 ^D^	94.27 ± 1.88 ^B^	81.03 ± 6.16 ^D^	92.53 ± 1.02 ^B^
APA16	87.23 ± 3.12 ^B^	91.02 ± 0.52 ^C^	87.68 ± 1.28 ^DE^	84.11 ± 4.55 ^CD^	85.27 ± 7.07 ^CD^
APA17	86.46 ± 0.38 ^B^	84.69 ± 1.28 ^E^	85.98 ± 0.21 ^E^	87.98 ± 0.92 ^BC^	83.79 ± 1.89 ^D^
APA18	93.89 ± 1.10 ^A^	98.19 ± 1.12 ^A^	97.56 ± 1.18 ^A^	97.69 ± 1.57 ^A^	99.64 ± 0.09 ^A^

*^a^* Percentage relative to pure HAB1 activity; *^b^* 10 μM ligand + 3 μM PYLs + 1 μM HAB1.

**Table 7:** Obtained total score, crash, and polar value of docking results.

Compd.	Total Score		Crash		Polar		Cscore		Similarity	
	PYR1	PYL10	PYR1	PYL10	PYR1	PYL10	PYR1	PYL10	PYR1	PYL10
ABA	10.96	10.53	−0.92	−1.53	6.67	5.6	81.72	42.5	0.72	0.81
*iso*-PhABA	15.74	11.48	−1	−2.61	9.4	7.44	75.09	45.19	0.96	0.9
APA6	16.31	12.6	−3.16	−3.21	9.17	7.09	127.31	75.3	0.85	0.79
APA11	14.61	16.64	−4.66	−2.24	8.32	9.27	128.27	73.38	0.79	0.85
APA14	15.7	10.64	−4.19	−4.73	8.24	6.22	148.6	76.65	0.79	0.77
APA15	10.44	13.88	−4.86	−2.79	4.87	7.24	159.56	87.43	0.84	0.77
APA16	14.25	11.62	−3.07	−2.87	7.67	7.19	147.5	85.38	0.85	0.79
APA17	14.09	15.19	−3.59	−2.48	7.54	9.08	104.33	96.78	0.79	0.85
APA18	16.46	14.55	−2.65	−2.16	9.38	7.86	116.97	89.21	0.83	0.87

**Table 8:** Calculated binding free energies of *iso*-PhABA, APA16, and APA18 (kcal/mol) for PYL10.

	*iso*-PhABA	APA16	APA18
*E*_vdw_	−34.46 ± 2.19	−50.62 ± 1.72	−47.89 ± 1.71
*E*_ele_	−19.3 ± 3.80	−68.24 ± 7.32	−91.06 ± 8.08
*E*_pb_	28.09 ± 5.70	75.96 ± 4.90	83.41 ± 8.28
*E*_npolar_	−3.75 ± 0.07	−5.22 ± 0.13	−4.82 ± 0.05
∆_bind·cal_	−29.42 ± 3.50	−48.12 ± 4.68	−60.36 ± 1.14
∆_bind·exp_^a^	−5.48	−5.69	−6.01

^a^ ∆_bind·exp_ = RTln(*K*_d_), R and T are equal to 0.00198 kcal^−1^·K^−1^ and 298 K, respectively.

## Data Availability

Data are contained within the article and Supplementary Materials.

## Supplementary Materials

The following supporting information can be downloaded at: https://www.mdpi.com/article/10.3390/molecules29133129/s1, ^1^H NMR, ^13^C NMR, and HRMS spectrum data of target compounds (PDF).
