# Supplementary material for: The Discovery of Highly Efficient and Promising ABA Receptor Antagonists for Agricultural Applications Based on APAn Modification
Source: Molecules. 2024 Jul 1;29(13):3129. doi: 10.3390/molecules29133129 (PMC11243256; doi:10.3390/molecules29133129)
Supplement: Supplementary file 1 [file molecules-29-03129-s001.zip › molecules-3080847-SI.pdf]

The Discovery of Highly Efficient and Promising ABA Receptor Antagonists for Agricultural Applications Based on APAn Modification

Xiaobin Li[1], Xianjun Tang[1], Mian Wang[2], Xueqin Zhang[3], Yanjun Xu[1], Yiyi Li[1], Jiaqi Li[1], and Zhaohai Qin[1,*]

[1] *College of Science, China Agricultural University, Beijing 100193, China*

[2] *College of Food and Bioengineering, Xihua University, Chengdu 610039, Sichuan Province, China*

[3] *College of Biological Science, China Agricultural University, Beijing 100193, China*

***** Corresponding author: *College of Science, China Agricultural University, Beijing 100193, China;* orcid.org/0000-0001-7516-2432; Phone: 86-10-62732958; Email: qinzhaohai@263.net

Supporting Information

Table Contents

1. Physico-chemical data of the intermediates and APAn

2. ^{1}H NMR, ^{13}C NMR and HRMS spectra of APA7~APA18

3. MST analysis of the binding of APAn and control agents to ABA receptors

4. Calculated values of the decomposition free energy

5. Seed germination promotion activity

1. Physico-chemical data of the intermediates and APAn

7-methoxy-2,2-dimethyl-3,4-dihydronaphthalen-1(2H)-one (**II**). ^{1}H NMR (500 MHz, $CDCl_3$) δ 7.51 (d, *J* = 2.9 Hz, 1H), 7.11 (d, *J* = 8.5 Hz, 1H), 7.02 (dd, *J* = 8.4, 2.9 Hz, 1H), 3.81 (s, 3H), 2.90 (t, *J* = 6.3 Hz, 2H), 1.95 (t, *J* = 6.3 Hz, 2H), 1.20 (s, 6H). Reddish brown oil, yield 99 %.

7-methoxy-2,2-dimethyl-2,3-dihydronaphthalene-1,4-dione (**III**). ^{1}H NMR (500 MHz, $CDCl_3$) δ 7.95 (d, *J* = 8.7 Hz, 1H), 7.43 (d, *J* = 2.7 Hz, 1H), 7.17 (dd, *J* = 8.6, 2.7 Hz, 1H), 3.91 (s, 3H), 2.85 (s, 2H), 1.28 (s, 6H). White solid, yield: 82 % m.p. 125.0 - 126.0 ℃.

7-hydroxy-2,2-dimethyl-2,3-dihydronaphthalene-1,4-dione (**IV**). ^{1}H NMR (500 MHz, DMSO-d_6) δ 10.88 (s, 1H), 7.83 (d, *J* = 8.5 Hz, 1H), 7.27 (d, *J* = 2.6 Hz, 1H), 7.17 (dd, *J* = 8.4, 2.6 Hz, 1H), 2.91 (s, 2H), 1.19 (s, 6H). White solid, yield 88 % m.p. 135.0 - 136.0 ℃.

7-ethoxy-2,2-dimethyl-2,3-dihydronaphthalene-1,4-dione (**V-2**). ^{1}H NMR (500 MHz, $CDCl_3$) δ 7.98 (d, *J* = 8.7 Hz, 1H), 7.45 (d, *J* = 2.7 Hz, 1H), 7.19 (dd, *J* = 8.6, 2.6 Hz, 1H), 4.18 (q, *J* = 7.0 Hz, 2H), 2.88 (s, 2H), 1.47 (t, *J* = 7.0 Hz, 3H), 1.31 (s, 6H). Reddish brown solid, yield: 97 % m.p. 119.0 - 120.0 ℃.

2,2-dimethyl-7-propoxy-2,3-dihydronaphthalene-1,4-dione (**V-3**). ^{1}H NMR (500 MHz, $CDCl_3$) δ 7.96 (d, *J* = 8.6 Hz, 1H), 7.44 (d, *J* = 2.6 Hz, 1H), 7.18 (dd, *J* = 8.6, 2.7 Hz, 1H), 4.05 (t, *J* = 6.5 Hz, 2H), 2.86 (s, 2H), 1.84 (m, *J* = 13.9, 7.4, 6.5 Hz, 2H), 1.29 (s, 6H), 1.05 (t, *J* = 7.4 Hz, 3H). Reddish brown oil, yield 89 %.

7-butoxy-2,2-dimethyl-2,3-dihydronaphthalene-1,4-dione (**V-4**). ^{1}H NMR (500 MHz, $CDCl_3$) δ 7.96 (d, *J* = 8.6 Hz, 1H), 7.44 (d, *J* = 2.6 Hz, 1H), 7.18 (dd, *J* = 8.7, 2.6 Hz, 1H), 4.09 (t, *J* = 6.5 Hz, 2H), 2.87 (s, 2H), 1.89 – 1.71 (m, 2H), 1.59 – 1.42 (m, 2H), 1.29 (s, 6H), 0.98 (t, *J* = 7.4 Hz, 3H). Reddish brown oil, yield: 91 %.

2,2-dimethyl-7-(pentyloxy)-2,3-dihydronaphthalene-1,4-dione (**V-5**). ^{1}H NMR (500 MHz, $CDCl_3$) δ 7.96 (d, *J* = 8.6 Hz, 1H), 7.44 (d, *J* = 2.6 Hz, 1H), 7.18 (dd, *J* = 8.6, 2.6 Hz, 1H), 4.08 (t, *J* = 6.5 Hz, 2H), 2.87 (s, 2H), 2.08 – 1.69 (m, 2H), 1.50 – 1.35 (m, 4H), 1.32 – 1.24 (m, 6H), 0.93 (t, *J* = 7.1 Hz, 3H). Reddish brown oil, yield 91 %.

7-(hexyloxy)-2,2-dimethyl-2,3-dihydronaphthalene-1,4-dione (**V-6**). ^{1}H NMR (500 MHz, $CDCl_3$) δ 7.96 (d, *J* = 8.6 Hz, 1H), 7.44 (d, *J* = 2.6 Hz, 1H), 7.18 (dd, *J* = 8.6, 2.6 Hz, 1H), 4.08 (t, *J* = 6.5 Hz, 2H), 2.87 (s, 2H), 1.90 – 1.74 (m, 2H), 1.60 – 1.43 (m, 2H), 1.34 (m *J* = 3.1 Hz, 4H), 1.29 (s, 6H), 0.97 – 0.83 (m, 3H). Reddish brown oil, yield 88 %.

7-isopropoxy-2,2-dimethyl-2,3-dihydronaphthalene-1,4-dione (**V-7**). ^{1}H NMR (500 MHz, $CDCl_3$) δ 7.97 (d, *J* = 8.6 Hz, 1H), 7.44 (d, *J* = 2.6 Hz, 1H), 7.16 (dd, *J* = 8.7, 2.6 Hz, 1H), 4.74 (p, *J* = 6.1 Hz, 1H), 2.88 (s, 2H), 1.39 (d, *J* = 6.0 Hz, 6H), 1.31 (s, 6H). Reddish brown oil, yield 94%.

7-isobutoxy-2,2-dimethyl-2,3-dihydronaphthalene-1,4-dione (**V-8**). ^{1}H NMR (500 MHz, $CDCl_3$) δ 7.98 (d, *J* = 8.7 Hz, 1H), 7.45 (d, *J* = 2.6 Hz, 1H), 7.20 (dd, *J* = 8.7, 2.6 Hz, 1H), 3.86 (d, *J* = 6.5 Hz, 2H), 2.88 (s, 2H), 2.13 (m, *J* = 13.3, 6.7 Hz, 1H), 1.31 (s, 6H), 1.05 (d, *J* = 6.7 Hz, 6H). Reddish brown oil, yield 93 %.

7-(isopentyloxy)-2,2-dimethyl-2,3-dihydronaphthalene-1,4-dione (**V-9**). ^{1}H NMR (500 MHz, $CDCl_3$) δ 7.95 (d, *J* = 8.6 Hz, 1H), 7.43 (d, *J* = 2.7 Hz, 1H), 7.17 (dd, *J* = 8.6, 2.6 Hz, 1H), 4.10 (t, *J* = 6.6 Hz, 2H), 2.86 (s, 2H), 1.83 (dt, *J* = 13.4, 6.7 Hz, 1H), 1.70 (q, *J* = 6.7 Hz, 2H), 1.28 (s, 6H), 0.96 (d, *J* = 6.7 Hz, 6H). Reddish brown oil, yield 95 %.

7-(3,3-dimethylbutoxy)-2,2-dimethyl-2,3-dihydronaphthalene-1,4-dione (**V-10**). ^{1}H NMR (500 MHz, $CDCl_3$) δ 7.97 (d, *J* = 8.6 Hz, 1H), 7.45 (d, *J* = 2.6 Hz, 1H), 7.17 (dd, *J* = 8.6, 2.6 Hz, 1H), 4.14 (t, *J* = 7.2 Hz, 2H), 2.87 (s, 2H), 1.76 (t, *J* = 7.2 Hz, 2H), 1.30 (s, 6H), 1.00 (s, 9H). Reddish brown oil, yield 89 %.

2,2-dimethyl-7-(4-phenylbutoxy)-2,3-dihydronaphthalene-1,4-dione (**V-11**). ^{1}H NMR (500 MHz, $CDCl_3$) δ 7.89 (d, *J* = 8.6 Hz, 1H), 7.36 (d, *J* = 2.6 Hz, 1H), 7.25 – 7.17 (m, 2H), 7.16 – 7.06 (m, 4H), 4.02 (t, *J* = 6.0 Hz, 2H), 2.79 (s, 2H), 2.62 (t, *J* = 7.2 Hz, 2H), 1.77 (m, *J* = 14.0, 7.1, 2.7 Hz, 4H), 1.22 (s, 6H). Reddish brown oil, yield: 92 %.

2,2-dimethyl-7-((4-methylpentyl)oxy)-2,3-dihydronaphthalene-1,4-dione (**V-12**). ^{1}H NMR (500 MHz, $CDCl_3$) δ 7.96 (d, *J* = 8.6 Hz, 1H), 7.43 (d, *J* = 2.6 Hz, 1H), 7.17 (dd, *J* = 8.6, 2.6 Hz, 1H), 4.06 (t, *J* = 6.6 Hz, 2H), 2.86 (s, 2H), 1.86 – 1.71 (m, 2H), 1.60 (dt, *J* = 13.3, 6.7 Hz, 1H), 1.38 – 1.31 (m, 2H), 1.29 (s, 6H), 0.91 (d, *J* = 6.7 Hz, 6H). Reddish brown oil, yield 87 %.

2,2-dimethyl-7-((3-methylpentyl)oxy)-2,3-dihydronaphthalene-1,4-dione (**V-13**). ^{1}H NMR (500 MHz, $CDCl_3$) δ 7.96 (d, *J* = 8.6 Hz, 1H), 7.44 (d, *J* = 2.6 Hz, 1H), 7.17 (dd, *J* = 8.6, 2.7 Hz, 1H), 4.21 – 3.97 (m, 2H), 2.86 (s, 2H), 1.85 (d, *J* = 8.0 Hz, 1H), 1.67 – 1.56 (m, 2H), 1.41 (m, *J* = 12.5, 7.5, 5.1 Hz, 1H), 1.29 (s, 6H), 1.26 – 1.20 (m, 1H), 0.94 (d, *J* = 6.2 Hz, 3H), 0.90 (s, 3H). Reddish brown oil, yield 93 %.

2,2-dimethyl-7-((3,5,5-trimethylhexyl)oxy)-2,3-dihydronaphthalene-1,4-dione (**V-14**). ^{1}H NMR (500 MHz, $CDCl_3$) δ 7.95 (d, *J* = 8.6 Hz, 1H), 7.43 (d, *J* = 2.7 Hz, 1H), 7.17 (dd, *J* = 8.6, 2.6 Hz, 1H), 4.09 (t, *J* = 6.5 Hz, 2H), 2.86 (s, 2H), 1.86 – 1.71 (m, 2H), 1.64 (dt, *J* = 13.3, 6.7 Hz, 1H), 1.28 (s, 6H), 1.24 (d,

J = 3.6 Hz, 1H), 1.11 (dd, J = 14.0, 6.0 Hz, 1H), 0.98 (d, J = 6.6 Hz, 3H), 0.88 (s, 9H). Reddish brown oil, yield 91%.

7-((3,7-dimethyloctyl)oxy)-2,2-dimethyl-2,3-dihydronaphthalene-1,4-dione (**V-15**). ^{1}H NMR (500 MHz, $CDCl_3$) δ 7.95 (d, J = 8.6 Hz, 1H), 7.43 (d, J = 2.7 Hz, 1H), 7.17 (dd, J = 8.6, 2.6 Hz, 1H), 4.21 – 3.95 (m, 2H), 2.86 (s, 2H), 1.84 (m, J = 14.1, 6.9, 5.1 Hz, 1H), 1.71 – 1.55 (m, 2H), 1.51 (dt, J = 13.3, 6.7 Hz, 1H), 1.36 – 1.29 (m, 2H), 1.28 (s, 6H), 1.26 – 1.23 (m, 1H), 1.18 – 1.11 (m, 3H), 0.93 (d, J = 6.5 Hz, 3H), 0.85 (d, J = 6.7 Hz, 6H). Reddish brown oil, yield 89 %.

7-((3,7-dimethyloct-6-en-1-yl)oxy)-2,2-dimethyl-2,3-dihydronaphthalene-1,4-dione (**V-16**) ^{1}H NMR (500 MHz, $CDCl_3$) δ 7.96 (d, J = 8.6 Hz, 1H), 7.44 (d, J = 2.7 Hz, 1H), 7.17 (dd, J = 8.6, 2.6 Hz, 1H), 5.16 – 4.97 (m, 1H), 4.30 – 3.95 (m, 2H), 2.86 (s, 2H), 2.08 – 1.94 (m, 2H), 1.85 (m, J = 13.8, 6.8, 5.1 Hz, 1H), 1.71 – 1.68 (m, 1H), 1.67 (d, J = 1.4 Hz, 3H), 1.66 – 1.61 (m, 1H), 1.59 (d, J = 1.4 Hz, 3H), 1.39 (m, J = 13.4, 9.5, 6.4, 5.4 Hz, 1H), 1.29 (s, 6H), 1.25 – 1.20 (m, 1H), 0.95 (d, J = 6.5 Hz, 3H). Reddish brown oil, yield 94 %.

7-(2-ethoxyethoxy)-2,2-dimethyl-2,3-dihydronaphthalene-1,4-dione (**V-17**). ^{1}H NMR (500 MHz, $CDCl_3$) δ 7.98 (d, J = 8.6 Hz, 1H), 7.49 (d, J = 2.6 Hz, 1H), 7.25 (dd, J = 8.7, 2.7 Hz, 1H), 4.31 – 4.19 (m, 2H), 3.89 – 3.77 (m, 2H), 3.61 (q, J = 7.0 Hz, 2H), 2.88 (s, 2H), 1.30 (s, 6H), 1.25 (t, J = 7.0 Hz, 3H). Reddish brown oil, yield 90 %.

7-(2-(2-methoxyethoxy)ethoxy)-2,2-dimethyl-2,3-dihydronaphthalene-1,4-dione (**V-18**) ^{1}H NMR (500 MHz, $CDCl_3$) δ 7.93 (d, J = 8.6 Hz, 1H), 7.43 (d, J = 2.6 Hz, 1H), 7.19 (dd, J = 8.6, 2.7 Hz, 1H), 4.29 – 4.17 (m, 2H), 3.96 – 3.82 (m, 2H), 3.75 – 3.61 (m, 2H), 3.61 – 3.49 (m, 2H), 3.35 (s, 3H), 2.84 (s, 2H), 1.26 (s, 6H). Reddish brown oil, yield 94 %.

(Z)-4-hydroxy-4-(5-hydroxy-3-methylpent-3-en-1-yn-1-yl)-7-methoxy-2,2-dimethyl-3,4-dihydronaph-thalen-1(2H)-one (**VI-1**). ^{1}H NMR (500 MHz, $CDCl_3$) δ 7.95 (d, J = 8.6 Hz, 1H), 7.37 (d, J = 2.6 Hz, 1H), 6.88 (dd, J = 8.6, 2.5 Hz, 1H), 5.88 (t, J = 7.0 Hz, 1H), 4.25 (s, 2H), 3.86 (s, 3H), 3.75 – 3.67 (m, 1H), 2.64 (m, J = 164.1, 24.2 Hz, 3H), 2.02 – 1.77 (m, 3H), 1.14 (s, 6H). Yellow oil, yield 49 %.

(Z)-7-ethoxy-4-hydroxy-4-(5-hydroxy-3-methylpent-3-en-1-yn-1-yl)-2,2-dimethyl-3,4-dihydronaph-thalen-1(2H)-one (**VI-2**). ^{1}H NMR (500 MHz, $CDCl_3$) δ 7.99 (d, J = 8.6 Hz, 1H), 7.37 (d, J = 2.5 Hz, 1H), 6.91 (dd, J = 8.7, 2.6 Hz, 1H), 5.92 (q, J = 7.0, 5.2 Hz, 1H), 4.29 (d, J = 6.0 Hz, 2H), 4.14 (q, J = 7.0 Hz, 2H), 2.71 (m, J = 98.1 Hz, 3H), 2.01 – 1.67 (m, 3H), 1.68 (s, 1H), 1.45 (t, J = 7.0 Hz, 3H), 1.18 (s, 6H). Yellow oil, yield 48 %.

(Z)-4-hydroxy-4-(5-hydroxy-3-methylpent-3-en-1-yn-1-yl)-2,2-dimethyl-7-propoxy-3,4-dihydronaphthalen-1(2H)-one (**VI-3**) ^{1}H NMR (500 MHz, $CDCl_3$) δ 7.96 (d, *J* = 8.6 Hz, 1H), 7.36 (d, *J* = 2.6 Hz, 1H), 6.89 (dd, *J* = 8.7, 2.6 Hz, 1H), 6.16 – 5.71 (m, 1H), 4.26 (d, *J* = 6.1 Hz, 2H), 4.01 (td, *J* = 6.5, 1.1 Hz, 2H), 3.36 – 3.11 (m, 1H), 2.80 (s, 1H), 2.60 (s, 1H), 2.05 (s, 1H), 1.89 (d, *J* = 1.4 Hz, 3H), 1.83 (q, *J* = 7.0 Hz, 2H), 1.16 (s, 6H), 1.04 (t, *J* = 7.4 Hz, 3H). Yellow oil, yield 43 %.

(Z)-7-butoxy-4-hydroxy-4-(5-hydroxy-3-methylpent-3-en-1-yn-1-yl)-2,2-dimethyl-3,4-dihydronaphthalen-1(2H)-one (**VI-4**). ^{1}H NMR (500 MHz, $CDCl_3$) δ 7.94 (d, *J* = 8.7 Hz, 1H), 7.35 (d, *J* = 2.5 Hz, 1H), 6.87 (dd, *J* = 8.7, 2.5 Hz, 1H), 6.00 – 5.75 (m, 1H), 4.25 (d, *J* = 6.8 Hz, 2H), 4.04 (t, *J* = 6.5 Hz, 2H), 3.53 (s, 1H), 2.78 (s, 1H), 2.59 (s, 1H), 2.36 (s, 1H), 1.91 – 1.83 (m, 3H), 1.77 (m, *J* = 8.9, 6.6 Hz, 2H), 1.48 (h, *J* = 7.4 Hz, 2H), 1.15 (s, 6H), 0.96 (t, *J* = 7.4 Hz, 3H). Yellow oil, yield 45 %.

(Z)-4-hydroxy-4-(5-hydroxy-3-methylpent-3-en-1-yn-1-yl)-2,2-dimethyl-7-(pentyloxy)-3,4-dihydronaphthalen-1(2H)-one (**VI-5**). ^{1}H NMR (500 MHz, $CDCl_3$) δ 7.97 (d, *J* = 8.7 Hz, 1H), 7.36 (d, *J* = 2.6 Hz, 1H), 6.90 (dd, *J* = 8.7, 2.5 Hz, 1H), 5.92 (dt, *J* = 13.1, 4.3 Hz, 1H), 4.30 (dd, *J* = 28.1, 6.3 Hz, 2H), 4.05 (td, *J* = 6.6, 1.1 Hz, 2H), 3.10 (s, 1H), 2.80 (s, 1H), 2.61 (s, 1H), 1.94 – 1.86 (m, 3H), 1.84 – 1.77 (m, 3H), 1.48 – 1.34 (m, 4H), 1.17 (s, 6H), 0.93 (t, *J* = 7.1 Hz, 3H). Yellow oil, yield 44 %.

(Z)-7-(hexyloxy)-4-hydroxy-4-(5-hydroxy-3-methylpent-3-en-1-yn-1-yl)-2,2-dimethyl-3,4-dihydronaphthalen-1(2H)-one (**VI-6**). ^{1}H NMR (500 MHz, $CDCl_3$) δ 7.98 (d, *J* = 8.7 Hz, 1H), 7.37 (d, *J* = 2.5 Hz, 1H), 6.91 (dd, *J* = 8.7, 2.6 Hz, 1H), 6.11 – 5.86 (m, 1H), 4.45 – 4.27 (m, 2H), 4.06 (t, *J* = 6.6 Hz, 2H), 2.81 (s, 1H), 2.62 (s, 1H), 1.94 – 1.89 (m, 4H), 1.81 (dd, *J* = 8.6, 6.4 Hz, 2H), 1.75 (s, 1H), 1.51 – 1.43 (m, 2H), 1.35 (dt, *J* = 7.5, 3.8 Hz, 4H), 1.18 (s, 6H), 0.97 – 0.88 (m, 3H). Yellow oil, yield 42 %.

(Z)-4-hydroxy-4-(5-hydroxy-3-methylpent-3-en-1-yn-1-yl)-7-isopropoxy-2,2-dimethyl-3,4-dihydronaphthalen-1(2H)-one (**VI-7**). ^{1}H NMR (500 MHz, $CDCl_3$) δ 7.94 (d, *J* = 8.7 Hz, 1H), 7.34 (d, *J* = 2.5 Hz, 1H), 6.85 (dd, *J* = 8.7, 2.5 Hz, 1H), 5.89 (t, *J* = 6.9 Hz, 1H), 4.67 (p, *J* = 6.1 Hz, 1H), 4.26 (d, *J* = 6.7 Hz, 2H), 3.43 (s, 1H), 2.79 (s, 1H), 2.57 (s, 1H), 2.28 (s, 1H), 1.87 (s, 3H), 1.35 (t, *J* = 6.5 Hz, 6H), 1.15 (d, *J* = 10.0 Hz, 6H). Yellow oil, yield 45 %.

(Z)-4-hydroxy-4-(5-hydroxy-3-methylpent-3-en-1-yn-1-yl)-7-isobutoxy-2,2-dimethyl-3,4-dihydronaphthalen-1(2H)-one (**VI-8**). ^{1}H NMR (500 MHz, $CDCl_3$) δ 7.97 (d, *J* = 8.7 Hz, 1H), 7.38 (d, *J* = 2.6 Hz, 1H), 6.90 (dd, *J* = 8.7, 2.5 Hz, 1H), 5.91 (t, *J* = 7.0 Hz, 1H), 4.28 (d, *J* = 6.6 Hz, 2H), 3.82 (d, *J* = 6.6 Hz, 2H), 3.40 (s, 1H), 2.82 (d, *J* = 20.2 Hz, 1H), 2.61 (s, 1H), 2.11 (m, 2H), 1.93 – 1.86 (m, 2H), 1.82 (s, 1H), 1.17 (s, 6H), 1.04 (dd, *J* = 6.8, 2.5 Hz, 6H). Yellow oil, yield 47 %.

(Z)-4-hydroxy-4-(5-hydroxy-3-methylpent-3-en-1-yn-1-yl)-7-(isopentyloxy)-2,2-dimethyl-3,4-dihydro-naphthalen-1(2H)-one (**VI-9**). ^{1}H NMR (500 MHz, $CDCl_3$) δ 7.95 (d, *J* = 8.6 Hz, 1H), 7.35 (d, *J* = 2.6 Hz, 1H), 6.88 (dd, *J* = 8.6, 2.5 Hz, 1H), 5.89 (t, *J* = 7.4 Hz, 1H), 4.25 (d, *J* = 6.6 Hz, 2H), 4.10 (t, *J* = 7.3 Hz, 2H), 3.55 (s, 1H), 2.79 (s, 1H), 2.35 (s, 1H), 2.19 (s, 1H), 1.79 (s, 3H), 1.74 - 1.66 (m, 1H), 1.64 - 1.57 (m, 1H), 1.52 - 1.41 (m, 1H), 1.36 (s, 3H), 1.31 (s, 3H), 0.92 (d, J = 6.8 Hz, 3H), 0.87 (d, J = 6.8 Hz, 3H). Yellow oil, yield 44 %.

(Z)-7-(3,3-dimethylbutoxy)-4-hydroxy-4-(5-hydroxy-3-methylpent-3-en-1-yn-1-yl)-2,2-dimethyl-3,4-dihydronaphthalen-1(2H)-one (**VI-10**). ^{1}H NMR (500 MHz, $CDCl_3$) δ 7.95 (d, *J* = 8.6 Hz, 1H), 7.35 (d, *J* = 2.6 Hz, 1H), 6.88 (dd, *J* = 8.6, 2.5 Hz, 1H), 5.89 (t, *J* = 7.4 Hz, 1H), 4.25 (d, *J* = 6.6 Hz, 2H), 4.10 (t, *J* = 7.3 Hz, 2H), 3.55 (s, 1H), 2.79 (s, 1H), 2.35 (s, 1H), 2.18 – 2.04 (m, 1H), 1.94 – 1.83 (m, 3H), 1.73 (t, *J* = 7.3 Hz, 2H), 1.15 (s, 6H), 0.98 (s, 9H). Yellow oil, yield 44 %.

(Z)-4-hydroxy-4-(5-hydroxy-3-methylpent-3-en-1-yn-1-yl)-2,2-dimethyl-7-(4-phenylbutoxy)-3,4-dihydronaphthalen-1(2H)-one (**VI-11**) ^{1}H NMR (500 MHz, $CDCl_3$). δ 7.96 (d, *J* = 8.7 Hz, 1H), 7.37 (d, *J* = 2.5 Hz, 1H), 7.31 – 7.24 (m, 2H), 7.19 (d, *J* = 7.4 Hz, 3H), 6.87 (dd, *J* = 8.7, 2.5 Hz, 1H), 5.89 (t, *J* = 7.0 Hz, 1H), 4.32 – 4.22 (m, 2H), 4.05 (t, *J* = 5.9 Hz, 2H), 3.59 (s, 1H), 2.80 (s, 1H), 2.69 (t, *J* = 7.1 Hz, 2H), 2.59 (d, *J* = 15.4 Hz, 1H), 2.40 (s, 1H), 1.99 – 1.85 (m, 3H), 1.85 – 1.80 (m, 4H), 1.16 (s, 6H). Yellow oil, yield 47 %.

(Z)-4-hydroxy-4-(5-hydroxy-3-methylpent-3-en-1-yn-1-yl)-2,2-dimethyl-7-((4-methylpentyl)oxy)-3,4-dihydronaphthalen-1(2H)-one (**VI-12**). ^{1}H NMR (500 MHz, $CDCl_3$) δ 7.96 (d, *J* = 8.7 Hz, 1H), 7.37 (d, *J* = 2.6 Hz, 1H), 6.89 (dd, *J* = 8.7, 2.6 Hz, 1H), 5.91 (d, *J* = 6.9 Hz, 1H), 4.27 (d, *J* = 6.7 Hz, 2H), 4.03 (t, *J* = 6.7 Hz, 2H), 3.61 (s, 1H), 2.79 (s, 1H), 2.60 (s, 1H), 2.45 (d, *J* = 40.1 Hz, 1H), 1.92 – 1.87 (m, 3H), 1.85 – 1.75 (m, 2H), 1.61 (dp, *J* = 13.4, 6.7 Hz, 1H), 1.39 – 1.30 (m, 2H), 1.17 (s, 6H), 0.92 (d, *J* = 6.7 Hz, 6H). Yellow oil, yield 47 %.

(Z)-4-hydroxy-4-(5-hydroxy-3-methylpent-3-en-1-yn-1-yl)-2,2-dimethyl-7-((3-methylpentyl)oxy)-3,4-dihydronaphthalen-1(2H)-one (**VI-13**). ^{1}H NMR (500 MHz, $CDCl_3$) δ 7.96 (d, *J* = 8.6 Hz, 1H), 7.36 (d, *J* = 2.5 Hz, 1H), 6.89 (dd, *J* = 8.6, 2.5 Hz, 1H), 5.90 (t, *J* = 7.1 Hz, 1H), 4.26 (d, *J* = 6.8 Hz, 2H), 4.08 (dd, *J* = 6.5, 3.1 Hz, 2H), 3.39 (s, 1H), 2.78 (s, 1H), 2.63 (d, *J* = 28.7 Hz, 1H), 2.21 (s, 1H), 2.01 (s, 1H), 1.88 (s, 3H), 1.60 (td, *J* = 8.7, 4.3 Hz, 2H), 1.40 (m, *J* = 13.0, 7.5, 5.1 Hz, 1H), 1.27 – 1.11 (m, 7H), 0.93 (d, *J* = 6.0 Hz, 3H), 0.90 (t, *J* = 7.4 Hz, 3H). Yellow oil, yield 47 %.

(Z)-4-hydroxy-4-(5-hydroxy-3-methylpent-3-en-1-yn-1-yl)-2,2-dimethyl-7-((3,5,5-trimethylhexyl)oxy)-3,4-dihydronaphthalen-1(2H)-one (**VI-14**). ^{1}H NMR (500 MHz, $CDCl_3$) δ 7.95 (d, J = 8.6 Hz, 1H), 7.36 (d, J = 2.6 Hz, 1H), 6.88 (dd, J = 8.7, 2.5 Hz, 1H), 5.89 (t, J = 6.9 Hz, 1H), 4.26 (d, J = 6.4 Hz, 2H), 4.12 – 3.95 (m, 2H), 3.38 (s, 1H), 2.79 (s, 1H), 2.59 (s, 1H), 2.36 – 2.18 (m, 1H), 1.88 (s, 3H), 1.81 (dt, J = 12.9, 6.5 Hz, 1H), 1.74 (dt, J = 6.5, 3.3 Hz, 1H), 1.62 (dd, J = 13.5, 6.8 Hz, 1H), 1.27 – 1.23 (m, 1H), 1.20 – 1.08 (m, 6H), 0.98 (d, J = 6.6 Hz, 3H), 0.89 (d, J = 1.2 Hz, 9H), 0.86 – 0.84 (m, 1H). Yellow oil, yield 41 %.

(Z)-7-((3,7-dimethyloctyl)oxy)-4-hydroxy-4-(5-hydroxy-3-methylpent-3-en-1-yn-1-yl)-2,2-dimethyl-3,4-dihydronaphthalen-1(2H)-one (**VI-15**). ^{1}H NMR (500 MHz, $CDCl_3$) δ 7.96 (d, J = 8.6 Hz, 1H), 7.36 (d, J = 2.5 Hz, 1H), 6.89 (dd, J = 8.7, 2.5 Hz, 1H), 5.90 (t, J = 6.7 Hz, 1H), 4.26 (d, J = 6.5 Hz, 2H), 4.08 (m, J = 6.4, 2.3 Hz, 2H), 3.26 (s, 1H), 2.79 (s, 1H), 2.60 (s, 1H), 2.11 (s, 1H), 1.92 – 1.77 (m, 4H), 1.72 – 1.45 (m, 3H), 1.36 – 1.11 (m, 12H), 0.94 (d, J = 6.5 Hz, 3H), 0.86 (d, J = 6.6 Hz, 6H). Yellow oil, yield 44 %.

(Z)-7-((3,7-dimethyloct-6-en-1-yl)oxy)-4-hydroxy-4-(5-hydroxy-3-methylpent-3-en-1-yn-1-yl)-2,2-dimethyl-3,4-dihydronaphthalen-1(2H)-one (**VI-16**). ^{1}H NMR (500 MHz, $CDCl_3$) δ 7.95 (d, J = 8.7 Hz, 1H), 7.36 (d, J = 2.5 Hz, 1H), 6.88 (dd, J = 8.7, 2.6 Hz, 1H), 5.89 (t, J = 6.9 Hz, 1H), 5.16 – 5.00 (m, 1H), 4.25 (d, J = 6.7 Hz, 2H), 4.07 (m, J = 6.3, 3.1 Hz, 2H), 3.60 (d, J = 6.1 Hz, 1H), 3.59 – 3.55 (m, 1H), 2.78 (s, 1H), 2.60 (s, 1H), 2.37 (s, 1H), 2.06 – 1.94 (m, 2H), 1.87 (s, 3H), 1.68 – 1.65 (m, 3H), 1.59 (s, 3H), 1.45 – 1.39 (m, 1H), 1.24 – 1.11 (m, 7H), 0.95 (d, J = 6.5 Hz, 3H), 0.89 (t, J = 7.1 Hz, 2H). Yellow oil, yield 42 %.

(Z)-7-(2-ethoxyethoxy)-4-hydroxy-4-(5-hydroxy-3-methylpent-3-en-1-yn-1-yl)-2,2-dimethyl-3,4-dihydronaphthalen-1(2H)-one (**VI-17**). ^{1}H NMR (500 MHz, $CDCl_3$) δ 7.95 (d, J = 8.7 Hz, 1H), 7.41 (d, J = 2.6 Hz, 1H), 6.91 (dd, J = 8.7, 2.6 Hz, 1H), 5.89 (t, J = 6.8 Hz, 1H), 4.25 (d, J = 6.7 Hz, 2H), 4.19 (dd, J = 5.6, 4.0 Hz, 2H), 3.79 (dd, J = 5.7, 4.0 Hz, 2H), 3.58 (q, J = 7.0 Hz, 2H), 3.42 (s, 1H), 2.80 (s, 1H), 2.56 (d, J = 18.1 Hz, 1H), 2.42 – 2.23 (m, 1H), 1.87 (s, 3H), 1.25 – 1.11 (m, 9H). Yellow oil, yield 47 %.

(Z)-4-hydroxy-4-(5-hydroxy-3-methylpent-3-en-1-yn-1-yl)-7-(2-(2-methoxyethoxy)ethoxy)-2,2-dimethyl-3,4-dihydronaphthalen-1(2H)-one (**VI-18**). ^{1}H NMR (500 MHz, $CDCl_3$) δ 7.93 (d, J = 8.6 Hz, 1H), 7.42 (d, J = 2.5 Hz, 1H), 6.89 (dd, J = 8.6, 2.5 Hz, 1H), 5.88 (t, J = 6.9 Hz, 1H), 4.30 – 4.23 (m, 2H), 4.21 (dd, J = 5.7, 4.0 Hz, 2H), 3.84 (dd, J = 5.7, 3.9 Hz, 2H), 3.67 (dd, J = 5.5, 3.8 Hz, 2H), 3.61 (s,

1H), 3.57 – 3.52 (m, 2H), 3.34 (s, 3H), 2.81 (s, 1H), 2.56 (s, 2H), 1.87 (s, 3H), 1.12 (s, 6H). Yellow oil, yield 46 %.

1-((1E,3Z)-5-hydroxy-3-methylpenta-1,3-dien-1-yl)-6-methoxy-3,3-dimethyl-1,2,3,4-tetrahydronaphthalene-1,4-diol (**VII-1**) ^{1}H NMR (500 MHz, DMSO-d_6). δ 7.38 (d, *J* = 8.6 Hz, 1H), 6.90 (d, *J* = 2.7 Hz, 1H), 6.80 (dd, *J* = 8.6, 2.8 Hz, 1H), 6.38 (d, *J* = 15.6 Hz, 1H), 5.92 (d, *J* = 15.5 Hz, 1H), 5.37 (t, *J* = 6.6 Hz, 1H), 5.07 (d, *J* = 6.8 Hz, 1H), 5.00 (s, 1H), 4.57 (t, *J* = 5.2 Hz, 2H), 4.02 (dd, *J* = 6.9, 5.1 Hz, 2H), 3.70 (s, 3H), 1.80 (dd, *J* = 13.3, 6.8 Hz, 1H), 1.76 – 1.68 (m, 4H), 0.88 (d, *J* = 16.8 Hz, 6H). Light yellow solid, yield 59 % m.p. 138.0 - 139.0 °C.

6-ethoxy-1-((1E,3Z)-5-hydroxy-3-methylpenta-1,3-dien-1-yl)-3,3-dimethyl-1,2,3,4-tetrahydronaphthalene-1,4-diol (**VII-2**). ^{1}H NMR (500 MHz, DMSO-d_6) δ 7.36 (d, *J* = 8.4 Hz, 1H), 6.87 (d, *J* = 2.7 Hz, 1H), 6.78 (dd, *J* = 8.5, 2.7 Hz, 1H), 6.38 (d, *J* = 15.6 Hz, 1H), 5.91 (d, *J* = 15.6 Hz, 1H), 5.37 (t, *J* = 6.6 Hz, 1H), 5.05 (d, *J* = 6.7 Hz, 1H), 4.97 (s, 1H), 4.57 (q, *J* = 4.5, 3.7 Hz, 2H), 4.02 (dd, *J* = 6.9, 4.8 Hz, 2H), 3.95 (dt, *J* = 6.8, 3.4 Hz, 2H), 1.83 – 1.75 (m, 1H), 1.67 (d, *J* = 41.3 Hz, 4H), 1.29 (t, *J* = 7.0 Hz, 3H), 0.87 (d, *J* = 14.8 Hz, 6H). Light yellow solid, yield 64 % m.p. 135.0 - 136.0 °C.

1-((1E,3Z)-5-hydroxy-3-methylpenta-1,3-dien-1-yl)-3,3-dimethyl-6-propoxy-1,2,3,4-tetrahydronaphthalene-1,4-diol (**VII-3**). ^{1}H NMR (500 MHz, $CDCl_3$) δ 7.45 (d, *J* = 8.5 Hz, 1H), 6.95 (d, *J* = 2.7 Hz, 1H), 6.86 (dd, *J* = 8.5, 2.7 Hz, 1H), 6.40 (d, *J* = 15.7 Hz, 1H), 6.01 (d, *J* = 15.6 Hz, 1H), 5.55 (t, *J* = 7.1 Hz, 1H), 4.81 (d, *J* = 7.9 Hz, 1H), 4.16 (dd, *J* = 6.8, 3.0 Hz, 2H), 3.90 (td, *J* = 6.6, 3.1 Hz, 2H), 2.06 (dd, *J* = 13.6, 6.8 Hz, 2H), 1.89 (s, 1H), 1.83 (s, 3H), 1.81 – 1.74 (m, 3H), 1.48 (s, 1H), 1.05 – 1.01 (m, 6H), 0.99 (s, 3H). Light yellow solid, yield 56 % m.p. 132.0 - 133.0 °C.

6-butoxy-1-((1E,3Z)-5-hydroxy-3-methylpenta-1,3-dien-1-yl)-3,3-dimethyl-1,2,3,4-tetrahydronaphthalene-1,4-diol (**VII-4**). ^{1}H NMR (500 MHz, $CDCl_3$) δ 7.42 (d, *J* = 8.6 Hz, 1H), 6.94 (d, *J* = 2.7 Hz, 1H), 6.83 (dd, *J* = 8.6, 2.6 Hz, 1H), 6.32 (d, *J* = 15.7 Hz, 1H), 6.00 (d, *J* = 15.7 Hz, 1H), 5.51 (t, *J* = 7.2 Hz, 1H), 4.81 – 4.73 (m, 1H), 4.13 – 4.02 (m, 2H), 3.92 (td, *J* = 6.5, 2.7 Hz, 2H), 2.63 (s, 1H), 2.13 (s, 1H), 2.05 – 1.96 (m, 2H), 1.81 (d, *J* = 1.4 Hz, 3H), 1.77 – 1.68 (m, 3H), 1.51 – 1.41 (m, 2H), 1.02 (s, 3H), 0.98 – 0.92 (m, 6H). Light yellow solid, yield 58 % m.p. 134.0 - 135.0 °C.

1-((1E,3Z)-5-hydroxy-3-methylpenta-1,3-dien-1-yl)-3,3-dimethyl-6-(pentyloxy)-1,2,3,4-tetrahydronaphthalene-1,4-diol (**VII-5**). ^{1}H NMR (500 MHz, $CDCl_3$) δ 7.44 (d, *J* = 8.6 Hz, 1H), 6.94 (d, *J* = 2.7 Hz, 1H), 6.85 (dd, *J* = 8.6, 2.7 Hz, 1H), 6.37 (d, *J* = 15.7 Hz, 1H), 6.00 (d, *J* = 15.7 Hz, 1H), 5.54 (t, *J* = 7.2 Hz, 1H), 4.80 (d, *J* = 7.6 Hz, 1H), 4.17 – 4.10 (m, 2H), 3.93 (td, *J* = 6.6, 3.0 Hz, 2H), 2.23 (d, *J* = 7.3

Hz, 1H), 2.03 (dd, J = 13.5, 6.8 Hz, 1H), 1.96 (s, 1H), 1.82 (s, 3H), 1.78 – 1.73 (m, 2H), 1.71 (s, 2H), 1.44 – 1.32 (m, 4H), 1.03 (s, 3H), 0.98 (s, 3H), 0.92 (t, J = 7.1 Hz, 3H). Light yellow solid, yield: 53 % m.p. 127.0 - 128.0 °C.

6-(hexyloxy)-1-((1E,3Z)-5-hydroxy-3-methylpenta-1,3-dien-1-yl)-3,3-dimethyl-1,2,3,4-tetrahydronaphthalene-1,4-diol (**VII-6**). ^{1}H NMR (500 MHz, $CDCl_3$) δ 7.46 – 7.40 (m, 1H), 6.94 (d, J = 2.7 Hz, 1H), 6.85 (dd, J = 8.6, 2.7 Hz, 1H), 6.36 (d, J = 15.7 Hz, 1H), 6.00 (d, J = 15.7 Hz, 1H), 5.54 (t, J = 7.2 Hz, 1H), 4.79 (d, J = 7.5 Hz, 1H), 4.12 (dt, J = 7.2, 3.5 Hz, 2H), 3.92 (td, J = 6.6, 3.0 Hz, 2H), 2.28 (d, J = 7.4 Hz, 1H), 2.06 – 2.03 (m, 1H), 1.98 (s, 1H), 1.82 (d, J = 1.3 Hz, 3H), 1.78 – 1.71 (m, 4H), 1.44 (m, J = 10.8, 5.0, 2.7 Hz, 2H), 1.32 (m, J = 4.7, 3.1 Hz, 4H), 1.03 (s, 3H), 0.98 (s, 3H), 0.91 – 0.86 (m, 3H). Light yellow solid, yield 51 % m.p. 131.0 - 132.0 °C.

1-((1E,3Z)-5-hydroxy-3-methylpenta-1,3-dien-1-yl)-6-isopropoxy-3,3-dimethyl-1,2,3,4-tetrahydronaphthalene-1,4-diol (**VII-7**). ^{1}H NMR (500 MHz, $CDCl_3$) δ 7.43 (d, J = 8.7 Hz, 1H), 6.94 (d, J = 2.6 Hz, 1H), 6.84 (dd, J = 8.7, 2.6 Hz, 1H), 6.37 (d, J = 15.7 Hz, 1H), 6.01 (d, J = 15.7 Hz, 1H), 5.53 (t, J = 7.2 Hz, 1H), 4.82 – 4.74 (m, 1H), 4.54 (p, J = 6.0 Hz, 1H), 4.13 (t, J = 7.1 Hz, 2H), 2.30 (d, J = 7.1 Hz, 1H), 2.06 – 2.00 (m, 2H), 1.98 (s, 1H), 1.82 (s, 3H), 1.79 (s, 1H), 1.32 (d, J = 6.1 Hz, 3H), 1.29 (d, J = 6.0 Hz, 3H), 1.03 (s, 3H), 0.99 (s, 3H). Light yellow solid, yield 60 % m.p. 134.0 - 135.0 °C.

1-((1E,3Z)-5-hydroxy-3-methylpenta-1,3-dien-1-yl)-6-isobutoxy-3,3-dimethyl-1,2,3,4-tetrahydronaphthalene-1,4-diol (**VII-8**). ^{1}H NMR (500 MHz, DMSO-d_6) δ 7.36 (d, J = 8.4 Hz, 1H), 6.88 (d, J = 2.7 Hz, 1H), 6.79 (dd, J = 8.5, 2.7 Hz, 1H), 6.38 (d, J = 15.6 Hz, 1H), 5.91 (d, J = 15.7 Hz, 1H), 5.37 (t, J = 6.6 Hz, 1H), 5.06 (d, J = 6.8 Hz, 1H), 4.99 (s, 1H), 4.56 (dt, J = 10.0, 6.1 Hz, 2H), 4.02 (dd, J = 6.9, 4.9 Hz, 2H), 3.71 – 3.59 (m, 2H), 1.98 (q, J = 7.0 Hz, 1H), 1.80 – 1.75 (m, 1H), 1.74 – 1.67 (m, 3H), 0.99 (s, 1H) 0.97 (t, J = 5.2 Hz, 6H), 0.87 (d, J = 16.0 Hz, 6H). Light yellow solid, yield 65 % m.p. 132.0 - 133.0 °C.

1-((1E,3Z)-5-hydroxy-3-methylpenta-1,3-dien-1-yl)-6-(isopentyloxy)-3,3-dimethyl-1,2,3,4-tetrahydronaphthalene-1,4-diol (**VII-9**). ^{1}H NMR (500 MHz, $CDCl_3$) δ 7.46 (d, J = 8.6 Hz, 1H), 6.97 (d, J = 2.6 Hz, 1H), 6.88 (dd, J = 8.6, 2.6 Hz, 1H), 6.37 (d, J = 15.4 Hz, 1H), 6.05 (d, J = 15.4 Hz, 1H), 5.53 (t, J = 7.3 Hz, 1H), 4.84 (s, 1H), 4.14 (d, J = 7.2 Hz, 2H), 4.03 (t, J = 7.4 Hz, 2H), 2.12 (m, 2H), 1.99 (s, 1H), 1.88 (d, J = 1.3 Hz, 3H), 1.81 (dd, J = 13.5, 9.2 Hz, 1H), 1.73 (t, J = 7.4 Hz, 2H), 1.42 (s, 1H), 1.24 (m, 1H), 1.02 (s, 3H), 0.99 (s, 3H), 0.98 (s, 6H). Light yellow solid, yield 61 % m.p. 122.0 - 123.0 °C.

6-(3,3-dimethylbutoxy)-1-((1E,3Z)-5-hydroxy-3-methylpenta-1,3-dien-1-yl)-3,3-dimethyl-1,2,3,4-tetrahydronaphthalene-1,4-diol (**VII-10**). ^{1}H NMR (500 MHz, $CDCl_3$) δ 7.45 (d, J = 8.5 Hz, 1H), 6.95 (d, J

= 2.7 Hz, 1H), 6.86 (dd, *J* = 8.6, 2.7 Hz, 1H), 6.39 (d, *J* = 15.7 Hz, 1H), 6.01 (d, *J* = 15.7 Hz, 1H), 5.56 (t, *J* = 7.2 Hz, 1H), 4.82 (s, 1H), 4.16 (d, *J* = 7.1 Hz, 2H), 4.00 (t, *J* = 7.3 Hz, 2H), 2.06 (m, 2H), 1.90 (s, 1H), 1.83 (d, *J* = 1.3 Hz, 3H), 1.78 (dd, *J* = 13.5, 9.2 Hz, 1H), 1.70 (t, *J* = 7.4 Hz, 2H), 1.46 (s, 1H), 1.04 (s, 3H), 0.99 (s, 3H), 0.98 (s, 9H). Light yellow solid, yield 56 % m.p. 121.0 - 122.0 °C.

1-((1E,3Z)-5-hydroxy-3-methylpenta-1,3-dien-1-yl)-3,3-dimethyl-6-(4-phenylbutoxy)-1,2,3,4-tetrahydronaphthalene-1,4-diol (**VII-11**). ^{1}H NMR (500 MHz, DMSO-d_6) δ 7.35 (d, *J* = 8.5 Hz, 1H), 7.28 (t, *J* = 7.6 Hz, 2H), 7.24 – 7.14 (m, 4H), 6.87 (d, *J* = 2.6 Hz, 1H), 6.78 (dd, *J* = 8.5, 2.7 Hz, 1H), 6.38 (d, *J* = 15.6 Hz, 1H), 5.91 (d, *J* = 15.6 Hz, 1H), 5.37 (t, *J* = 6.6 Hz, 1H), 5.05 (d, *J* = 6.8 Hz, 1H), 4.97 (s, 1H), 4.56 (dt, *J* = 10.7, 6.0 Hz, 2H), 4.02 (t, *J* = 6.0 Hz, 2H), 3.95 – 3.87 (m, 2H), 2.62 (t, *J* = 4.9 Hz, 2H), 1.70 (d, *J* = 3.6 Hz, 8H), 0.87 (d, *J* = 15.3 Hz, 6H). Light yellow solid, yield 62 % m.p. 142.0 - 143.0 °C.

1-((1E,3Z)-5-hydroxy-3-methylpenta-1,3-dien-1-yl)-3,3-dimethyl-6-((4-methylpentyl)oxy)-1,2,3,4-tetrahydronaphthalene-1,4-diol (**VII-12**). ^{1}H NMR (500 MHz, $CDCl_3$) δ 7.43 (d, *J* = 8.6 Hz, 1H), 6.95 (d, *J* = 2.7 Hz, 1H), 6.84 (dd, *J* = 8.5, 2.7 Hz, 1H), 6.32 (d, *J* = 15.7 Hz, 1H), 6.00 (d, *J* = 15.7 Hz, 1H), 5.52 (t, *J* = 7.1 Hz, 1H), 4.77 (dd, *J* = 9.3, 6.8 Hz, 1H), 4.09 (td, *J* = 10.5, 7.1 Hz, 2H), 3.91 (m, *J* = 5.7, 2.5 Hz, 2H), 2.65 (d, *J* = 15.7 Hz, 1H), 2.18 (s, 1H), 1.99 (m, *J* = 13.5, 6.8, 1.3 Hz, 1H), 1.81 (d, *J* = 1.4 Hz, 3H), 1.78 – 1.72 (m, 3H), 1.61 – 1.54 (m, 1H), 1.35 – 1.28 (m, 3H), 1.02 (s, 3H), 0.97 (s, 3H), 0.90 (d, *J* = 6.6 Hz, 6H). Light yellow solid, yield 57 % m.p. 130.0 - 131.0 °C.

1-((1E,3Z)-5-hydroxy-3-methylpenta-1,3-dien-1-yl)-3,3-dimethyl-6-((3-methylpentyl)oxy)-1,2,3,4-tetrahydronaphthalene-1,4-diol (**VII-13**). ^{1}H NMR (500 MHz, $CDCl_3$) δ 7.45 (d, *J* = 8.5 Hz, 1H), 6.95 (d, *J* = 2.7 Hz, 1H), 6.86 (dd, *J* = 8.5, 2.7 Hz, 1H), 6.39 (d, *J* = 15.6 Hz, 1H), 6.01 (d, *J* = 15.7 Hz, 1H), 5.55 (t, *J* = 7.1 Hz, 1H), 4.82 (d, *J* = 9.0 Hz, 1H), 4.21 – 4.10 (m, 2H), 3.97 (m, *J* = 6.5, 4.6 Hz, 2H), 2.05 (m, *J* = 16.5, 8.3 Hz, 2H), 1.89 (s, 1H), 1.85 – 1.72 (m, 5H), 1.59 – 1.52 (m, 2H), 1.51 – 1.34 (m, 2H), 1.21 (dt, *J* = 14.0, 7.2 Hz, 1H), 1.03 (s, 3H), 0.99 (s, 3H), 0.95 – 0.86 (m, 6H). Light yellow solid, yield 60 % m.p. 127.0 - 128.0 °C.

1-((1E,3Z)-5-hydroxy-3-methylpenta-1,3-dien-1-yl)-3,3-dimethyl-6-((3,5,5-trimethylhexyl)oxy)-1,2,3,4-tetrahydronaphthalene-1,4-diol (**VII-14**). ^{1}H NMR (500 MHz, $CDCl_3$) δ 7.42 (d, *J* = 8.5 Hz, 1H), 6.94 (d, *J* = 2.7 Hz, 1H), 6.83 (dd, *J* = 8.6, 2.6 Hz, 1H), 6.29 (dd, *J* = 15.7, 2.5 Hz, 1H), 6.00 (d, *J* = 15.7 Hz, 1H), 5.51 (t, *J* = 7.3 Hz, 1H), 4.77 (dd, *J* = 9.3, 6.8 Hz, 1H), 4.06 (dd, *J* = 9.2, 7.3 Hz, 2H), 3.94 (td, *J* = 6.6, 2.1 Hz, 2H), 2.65 (s, 1H), 2.14 (s, 1H), 1.99 (dd, *J* = 13.5, 6.8 Hz, 1H), 1.86 (dd, *J* = 18.8, 1.3 Hz, 1H), 1.81 (s, 3H), 1.79 – 1.69 (m, 3H), 1.57 (dd, *J* = 12.9, 6.6 Hz, 1H), 1.30 – 1.20 (m, 2H), 1.09 (dd, *J*

= 13.9, 5.9 Hz, 1H), 1.02 (s, 3H), 0.96 (d, *J* = 6.3 Hz, 5H), 0.88 (d, *J* = 1.2 Hz, 9H). Light yellow solid, yield 52 % m.p. 119.0 - 120.0 °C.

6-((3,7-dimethyloctyl)oxy)-1-((1E,3Z)-5-hydroxy-3-methylpenta-1,3-dien-1-yl)-3,3-dimethyl-1,2,3,4-tetrahydronaphthalene-1,4-diol (**VII-15**). ^{1}H NMR (500 MHz, $CDCl_3$) δ 7.44 (d, *J* = 8.5 Hz, 1H), 6.95 (d, *J* = 2.6 Hz, 1H), 6.85 (dd, *J* = 8.6, 2.7 Hz, 1H), 6.36 (dd, *J* = 15.7, 1.7 Hz, 1H), 6.01 (d, *J* = 15.7 Hz, 1H), 5.54 (t, *J* = 7.2 Hz, 1H), 4.80 (dd, *J* = 9.2, 6.7 Hz, 1H), 4.12 (dd, *J* = 7.2, 5.2 Hz, 2H), 3.96 (m, *J* = 6.7, 4.7 Hz, 2H), 2.07 – 1.99 (m, 2H), 1.87 – 1.75 (m, 6H), 1.65 (q, *J* = 6.2 Hz, 1H), 1.54 (m, *J* = 16.7, 13.2, 6.7 Hz, 2H), 1.34 – 1.24 (m, 4H), 1.19 – 1.12 (m, 3H), 1.03 (s, 3H), 0.98 (s, 3H), 0.93 (dd, *J* = 6.6, 1.5 Hz, 3H), 0.87 (d, *J* = 6.6 Hz, 6H). Light yellow solid, yield 54 % m.p. 134.0 - 135.0 °C.

6-((3,7-dimethyloct-6-en-1-yl)oxy)-1-((1E,3Z)-5-hydroxy-3-methylpenta-1,3-dien-1-yl)-3,3-dimethyl-1,2,3,4-tetrahydronaphthalene-1,4-diol (**VII-16**). ^{1}H NMR (500 MHz, $CDCl_3$) δ 7.44 (d, *J* = 8.6 Hz, 1H), 6.95 (d, *J* = 2.6 Hz, 1H), 6.85 (dd, *J* = 8.5, 2.7 Hz, 1H), 6.37 (dd, *J* = 15.7, 1.8 Hz, 1H), 6.01 (d, *J* = 15.7 Hz, 1H), 5.54 (t, *J* = 7.2 Hz, 1H), 5.10 (td, *J* = 7.2, 6.3, 3.5 Hz, 1H), 4.80 (d, *J* = 8.2 Hz, 1H), 4.14 (dd, *J* = 7.4, 3.2 Hz, 2H), 3.96 (qt, *J* = 7.2, 3.2 Hz, 2H), 2.17 (s, 1H), 2.07 – 1.99 (m, 2H), 1.95 – 1.93 (m, 1H), 1.82 (s, 3H), 1.68 (s, 6H), 1.60 (m, 5H), 1.37 (m, *J* = 10.0, 7.4, 5.0 Hz, 1H), 1.26 – 1.17 (m, 2H), 1.03 (s, 3H), 0.98 (s, 3H), 0.94 (dd, *J* = 6.8, 1.4 Hz, 3H). Light yellow solid, yield 62 % m.p. 115.0 - 116.0 °C.

6-(2-ethoxyethoxy)-1-((1E,3Z)-5-hydroxy-3-methylpenta-1,3-dien-1-yl)-3,3-dimethyl-1,2,3,4-tetrahydronaphthalene-1,4-diol (**VII-17**). ^{1}H NMR (500 MHz, $CDCl_3$) δ 7.37 (d, *J* = 8.6 Hz, 1H), 6.92 (d, *J* = 2.8 Hz, 1H), 6.80 (dd, *J* = 8.6, 2.7 Hz, 1H), 6.27 (d, *J* = 15.5 Hz, 1H), 5.92 (d, *J* = 15.8 Hz, 1H), 5.46 (t, *J* = 7.1 Hz, 1H), 4.71 (dd, *J* = 9.3, 6.7 Hz, 1H), 4.05 – 4.00 (m, 4H), 3.69 (t, *J* = 4.8 Hz, 2H), 3.55 – 3.49 (m, 2H), 2.05 – 1.98 (m, 1H), 1.94 (dd, *J* = 13.5, 6.8 Hz, 1H), 1.74 (s, 3H), 1.70 (dd, *J* = 13.5, 9.4 Hz, 1H), 1.20 – 1.13 (m, 5H), 0.95 (s, 3H), 0.90 (s, 3H). Light yellow solid, yield 61 % m.p. 145.0 - 146.0 °C.

1-((1E,3Z)-5-hydroxy-3-methylpenta-1,3-dien-1-yl)-6-(2-(2-methoxyethoxy)ethoxy)-3,3-dimethyl-1,2,3,4-tetrahydronaphthalene-1,4-diol (**VII-18**). ^{1}H NMR (500 MHz, DMSO-d_6) δ 7.37 (d, *J* = 8.6 Hz, 1H), 6.89 (d, *J* = 2.7 Hz, 1H), 6.80 (dd, *J* = 8.5, 2.7 Hz, 1H), 6.39 (d, *J* = 15.6 Hz, 1H), 5.91 (d, *J* = 15.6 Hz, 1H), 5.37 (t, *J* = 6.6 Hz, 1H), 5.08 (d, *J* = 6.8 Hz, 1H), 5.00 (s, 1H), 4.62 – 4.52 (m, 2H), 4.04-4.00 (m, 4H), 3.70 (t, *J* = 4.7 Hz, 2H), 3.60 – 3.53 (m, 2H), 3.48 – 3.41 (m, 2H), 3.24 (s, 3H), 1.84 – 1.75 (m, 1H), 1.71 (d, *J* = 1.6 Hz, 4H), 0.87 (d, *J* = 14.4 Hz, 6H). Light yellow solid, yield 64 % m.p. 136.0 - 137.0 °C.

Data for ***APA7***. White powder, yield 35%, m.p. 167 - 168 °C. ^{1}H NMR (500 MHz, $CDCl_3$) δ 8.01 (d, *J* = 8.7 Hz, 1H), 7.79 (d, *J* = 16.0 Hz, 1H), 7.00 (s, 1H), 6.87 (dd, *J* = 8.7, 2.5 Hz, 1H), 6.40 (d, *J* = 16.0 Hz, 1H), 5.74 (s, 1H), 4.66 (p, *J* = 6.1 Hz, 1H), 2.71 (s, 1H), 2.58 (d, *J* = 17.0 Hz, 1H), 2.04 (s, 3H), 1.35 (dd, *J* = 13.1, 6.1 Hz, 6H), 1.08 (d, *J* = 13.8 Hz, 6H). ^{13}C NMR (126 MHz, $CDCl_3$) δ 195.90, 170.65, 163.17, 152.00, 148.46, 139.22, 129.47, 128.10, 124.13, 117.55, 114.75, 78.45, 70.19, 49.64, 41.11, 24.93, 24.33, 23.43, 21.98, 21.82, 20.09. HRMS (*m/z*) calcd. for $C_{21}H_{26}O_5$ $[M + H]^+$ 359.1853, found 359.1852.

Data for ***APA8***. White powder, yield 33%, m.p. 153 - 154 °C. ^{1}H NMR (500 MHz, DMSO-d_6) δ 12.08 (s, 1H), 7.82 (d, *J* = 8.7 Hz, 1H), 7.63 (d, *J* = 14.5 Hz, 1H), 7.04 (s, 1H), 6.96 (dd, *J* = 8.6, 2.7 Hz, 1H), 6.41 (d, *J* = 16.0 Hz, 1H), 5.65 (s, 1H), 5.59 (s, 1H), 3.81 (dd, *J* = 6.5, 1.9 Hz, 2H), 2.72 – 2.55 (m, 1H), 2.48 (s, 1H), 2.03 (td, *J* = 13.0, 6.3 Hz, 1H), 1.95 (s, 3H), 1.02 – 0.96 (m, 9H), 0.91 (s, 3H). ^{13}C NMR (126 MHz, DMSO-d_6) δ 195.61, 167.36, 163.71, 150.05, 149.56, 140.45, 128.61, 127.62, 124.60, 118.97, 113.98, 113.21, 77.62, 74.38, 49.81, 41.25, 40.25, 28.13, 24.50, 23.82, 21.25, 19.45. HRMS (*m/z*) calcd. for $C_{22}H_{28}O_5$ $[M + H]^+$ 373.2010, found 373.2004.

Data for ***APA9***. White powder, yield 37%, m.p. 162 - 163 °C. ^{1}H NMR (500 MHz, DMSO-d_6) δ 12.10 (s, 1H), 7.83 (d, *J* = 8.7 Hz, 1H), 6.98 (d, *J* = 8.5 Hz, 1H), 6.46 (d, *J* = 15.6 Hz, 1H), 6.36 (d, *J* = 15.8 Hz, 1H), 5.75 (s, 1H), 5.61 (s, 1H), 4.06 (t, *J* = 6.6 Hz, 2H), 2.63 (d, *J* = 14.2 Hz, 1H), 2.19 (s, 3H), 1.83 – 1.72 (m, 1H), 1.62 (q, *J* = 6.7 Hz, 2H), 0.98 (s, 3H), 0.95 – 0.87 (m, 9H). ^{13}C NMR (126 MHz, DMSO-d_6) δ 195.61, 168.18, 163.63, 150.97, 149.71, 139.32, 133.07, 128.74, 124.47, 120.61, 114.01, 113.22, 77.49, 66.71, 49.73, 41.21, 37.71, 24.99, 24.50, 23.84, 22.87, 22.84, 14.19. HRMS (*m/z*) calcd. for $C_{23}H_{30}O_5$ $[M + H]^+$ 387.2166, found 387.2160.

Data for ***APA10***. White powder, yield 45%, m.p. 134 - 135 °C. ^{1}H NMR (500 MHz, $CDCl_3$) δ 8.02 (d, *J* = 8.7 Hz, 1H), 7.76 (d, *J* = 16.0 Hz, 1H), 7.02 (s, 1H), 6.89 (dd, *J* = 8.7, 2.5 Hz, 1H), 6.39 (d, *J* = 16.0 Hz, 1H), 5.74 (s, 1H), 4.08 (t, *J* = 7.3 Hz, 2H), 2.68 (s, 1H), 2.59 (d, *J* = 17.1 Hz, 1H), 2.03 (s, 3H), 1.72 (t, *J* = 7.3 Hz, 2H), 1.10 (s, 3H), 1.06 (s, 3H), 0.98 (s, 9H). ^{13}C NMR (126 MHz, $CDCl_3$) δ 195.03, 169.61, 163.19, 150.84, 147.47, 138.17, 128.41, 127.20, 123.35, 116.67, 113.25, 111.12, 77.54, 64.73, 49.76, 48.66, 41.19, 40.13, 28.75, 28.69, 23.27, 22.40, 20.42. HRMS (*m/z*) calcd. for $C_{24}H_{32}O_5$ $[M + H]^+$ 401.2323, found 401.2322.

Data for ***APA11***. White powder, yield 42%, m.p. 133 - 134 °C. ^{1}H NMR (500 MHz, $CDCl_3$) δ 8.01 (d, *J* = 8.7 Hz, 1H), 7.76 (d, *J* = 15.9 Hz, 1H), 7.26 (d, *J* = 4.7 Hz, 2H), 7.21 – 7.14 (m, 3H), 7.01 (s, 1H), 6.87

(dd, J = 8.7, 2.5 Hz, 1H), 6.37 (d, J = 16.0 Hz, 1H), 5.72 (s, 1H), 4.01 (d, J = 3.0 Hz, 2H), 2.67 (t, J = 7.0 Hz, 3H), 2.58 (d, J = 17.3 Hz, 1H), 2.01 (s, 3H), 1.80 (dd, J = 7.7, 4.3 Hz, 4H), 1.07 (d, J = 20.0 Hz, 6H). ^{13}C NMR (126 MHz, $CDCl_3$) δ 195.98, 170.62, 164.21, 151.85, 148.45, 141.99, 139.08, 129.42, 128.39, 128.35, 128.22, 125.84, 124.42, 117.70, 114.25, 112.04, 78.55, 68.10, 49.66, 41.13, 35.51, 28.67, 27.71, 24.28, 23.40, 21.45. HRMS (*m/z*) calcd. for $C_{28}H_{32}O_5$ $[M + H]^+$ 449.2323, found 449.2309.

*Data for **APA12***. White powder, yield 39%, m.p. 135 - 136 °C. ^{1}H NMR (500 MHz, $CDCl_3$) δ 8.02 (d, J = 8.6 Hz, 1H), 7.77 (d, J = 16.0 Hz, 1H), 7.02 (s, 1H), 6.89 (dd, J = 8.7, 2.5 Hz, 1H), 6.39 (d, J = 16.0 Hz, 1H), 5.74 (s, 1H), 4.00 (td, J = 6.6, 3.5 Hz, 2H), 2.69 (s, 1H), 2.59 (d, J = 16.8 Hz, 1H), 2.03 (s, 3H), 1.82 – 1.74 (m, 2H), 1.59 (m, 1H), 1.36 – 1.29 (m, 2H), 1.08 (d, J = 18.7 Hz, 6H), 0.91 (d, J = 6.7 Hz, 6H). ^{13}C NMR (126 MHz, $CDCl_3$) δ 195.96, 170.57, 164.30, 151.91, 148.42, 139.13, 129.43, 128.18, 124.37, 117.63, 114.26, 112.05, 78.55, 68.65, 49.67, 41.14, 35.04, 27.82, 27.00, 24.29, 23.40, 22.52, 21.46. HRMS (*m/z*) calcd. for $C_{24}H_{32}O_5$ $[M + H]^+$ 401.2323, found 401.2306.

*Data for **APA13***. White powder, yield 38%, m.p. 152 - 153 °C. ^{1}H NMR (500 MHz, $CDCl_3$) δ 8.02 (d, J = 8.7 Hz, 1H), 7.77 (d, J = 16.0 Hz, 1H), 7.07 – 6.98 (m, 1H), 6.89 (dd, J = 8.7, 2.5 Hz, 1H), 6.39 (d, J = 16.0 Hz, 1H), 5.74 (s, 1H), 4.11 – 3.99 (m, 2H), 2.68 (s, 1H), 2.59 (d, J = 17.1 Hz, 1H), 2.06 – 1.99 (m, 3H), 1.87 – 1.78 (m, 1H), 1.64 – 1.54 (m, 2H), 1.40 (m, 1H), 1.25 – 1.16 (m, 1H), 1.08 (d, J = 17.6 Hz, 6H), 0.94 – 0.87 (m, 6H). ^{13}C NMR (126 MHz, $CDCl_3$) δ 195.01, 169.67, 163.28, 150.87, 147.47, 138.18, 128.40, 127.16, 123.34, 116.66, 113.26, 111.10, 77.54, 65.70, 48.66, 40.13, 34.59, 30.27, 28.45, 23.27, 22.39, 20.43, 18.09, 10.21. HRMS (*m/z*) calcd. for $C_{24}H_{32}O_5$ $[M + H]^+$ 401.2323, found 401.2300.

*Data for **APA14***. White powder, yield 35%, m.p. 125 - 126 °C. ^{1}H NMR (500 MHz, $CDCl_3$) δ 8.01 (d, J = 8.7 Hz, 1H), 7.82 – 7.69 (m, 1H), 7.02 (s, 1H), 6.88 (dd, J = 8.7, 2.5 Hz, 1H), 6.38 (d, J = 16.0 Hz, 1H), 5.73 (s, 1H), 4.02 (d, J = 6.8 Hz, 2H), 2.76 – 2.52 (m, 2H), 2.02 (s, 3H), 1.78 (m, 1H), 1.75 – 1.68 (m, 1H), 1.60 (dt, J = 13.4, 6.8 Hz, 1H), 1.28 – 1.18 (m, 2H), 1.09 (s, 3H), 1.05 (s, 3H), 0.97 (d, J = 6.6 Hz, 3H), 0.88 (d, J = 1.9 Hz, 9H). ^{13}C NMR (126 MHz, $CDCl_3$) δ 195.07, 169.60, 163.27, 150.85, 147.48, 138.20, 128.40, 127.13, 123.32, 116.66, 113.29, 111.08, 77.53, 76.22, 65.66, 50.13, 48.65, 40.13, 37.25, 30.11, 28.92, 26.28, 25.11, 23.28, 22.40, 21.66, 20.43. HRMS (*m/z*) calcd. for $C_{27}H_{38}O_5$ $[M + H]^+$ 443.2792, found 443.2779.

*Data for **APA15***. White powder, yield 34%, m.p. 132 - 133 °C. ^{1}H NMR (500 MHz, $CDCl_3$) δ 8.02 (d, J = 8.7 Hz, 1H), 7.76 (d, J = 16.0 Hz, 1H), 7.02 (s, 1H), 6.89 (dd, J = 8.7, 2.5 Hz, 1H), 6.38 (d, J = 16.0 Hz, 1H), 5.73 (s, 1H), 4.04 (tt, J = 9.1, 4.2 Hz, 2H), 2.68 (s, 1H), 2.58 (d, J = 17.1 Hz, 1H), 2.05 – 2.00

(m, 3H), 1.87 – 1.77 (m, 1H), 1.70 – 1.62 (m, 1H), 1.54 (m, 2H), 1.35 – 1.22 (m, 4H), 1.17 – 1.12 (m, 2H), 1.09 (s, 3H), 1.06 (s, 3H), 0.92 (dd, *J* = 6.6, 1.6 Hz, 3H), 0.86 (d, *J* = 6.7 Hz, 6H). ^{13}C NMR (126 MHz, $CDCl_3$) δ 196.05, 170.70, 164.31, 151.87, 139.21, 129.44, 128.19, 124.36, 117.70, 114.25, 112.17, 78.55, 66.71, 60.44, 49.68, 41.16, 39.22, 37.28, 36.05, 29.77, 27.96, 24.63, 24.30, 23.42, 22.65, 21.45, 19.61. HRMS (*m/z*) calcd. for $C_{28}H_{40}O_5$ $[M + H]^+$ 457.2949, found 457.2930.

*Data for **APA16***. White powder, yield 36%, m.p. 135 - 1136 °C. ^{1}H NMR (500 MHz, $CDCl_3$) δ 8.00 (d, *J* = 8.7 Hz, 1H), 7.76 (d, *J* = 16.0 Hz, 1H), 7.02 (s, 1H), 6.88 (dd, *J* = 8.8, 2.5 Hz, 1H), 6.38 (d, *J* = 16.0 Hz, 1H), 5.72 (s, 1H), 5.11 – 5.04 (m, 1H), 4.03 (q, *J* = 9.1, 7.8 Hz, 2H), 2.67 (s, 1H), 2.57 (d, *J* = 17.1 Hz, 1H), 2.07 – 1.91 (m, 5H), 1.85 – 1.76 (m, 1H), 1.66 (s, 3H), 1.59 (m, 4H), 1.40 – 1.31 (m, 1H), 1.28 – 1.12 (m, 2H), 1.09 (s, 3H), 1.05 (s, 3H), 0.93 (dd, *J* = 6.6, 1.4 Hz, 3H). ^{13}C NMR (126 MHz, $CDCl_3$) δ 195.10, 169.82, 163.27, 150.86, 147.50, 138.20, 130.35, 128.40, 127.17, 123.54, 116.71, 113.21, 111.15, 77.52, 65.61, 48.64, 40.14, 36.07, 34.93, 28.39, 24.70, 24.39, 23.27, 22.40, 20.44, 18.49, 16.65. HRMS (*m/z*) calcd. for $C_{28}H_{38}O_5$ $[M + H]^+$ 455.2792, found 455.2772.

*Data for **APA17***. White powder, yield 35%, m.p. 153 - 154 °C. ^{1}H NMR (500 MHz, $CDCl_3$) δ 8.02 (d, *J* = 8.7 Hz, 1H), 7.76 (d, *J* = 16.0 Hz, 1H), 7.08 (s, 1H), 6.93 (dd, *J* = 8.7, 2.6 Hz, 1H), 6.39 (d, *J* = 16.1 Hz, 1H), 5.73 (s, 1H), 4.18 (td, *J* = 4.5, 2.5 Hz, 2H), 3.79 (t, *J* = 4.7 Hz, 2H), 3.58 (q, *J* = 7.0 Hz, 2H), 2.70 (s, 1H), 2.58 (d, *J* = 17.1 Hz, 1H), 2.03 (s, 3H), 1.22 (t, *J* = 7.0 Hz, 3H), 1.08 (d, *J* = 16.7 Hz, 6H). ^{13}C NMR (126 MHz, $CDCl_3$) δ 196.03, 170.73, 163.90, 151.83, 148.43, 139.09, 129.37, 128.30, 124.67, 117.76, 114.41, 112.43, 78.50, 68.65, 67.70, 66.93, 49.68, 41.14, 24.30, 23.42, 21.48, 15.10. HRMS (*m/z*) calcd. for $C_{22}H_{28}O_6$ $[M + H]^+$ 389.1959, found 389.1942.

*Data for **APA18***. White powder, yield 31%, m.p .157 - 158 °C. ^{1}H NMR (500 MHz, $CDCl_3$) δ 7.99 (d, *J* = 8.7 Hz, 1H), 7.73 (d, *J* = 16.0 Hz, 1H), 7.07 (s, 1H), 6.90 (dd, *J* = 8.7, 2.6 Hz, 1H), 6.38 (d, *J* = 16.0 Hz, 1H), 5.72 (s, 1H), 4.19 (q, *J* = 4.3 Hz, 2H), 3.84 (t, *J* = 4.7 Hz, 2H), 3.72 – 3.65 (m, 2H), 3.55 (dd, *J* = 5.8, 3.1 Hz, 2H), 3.35 (s, 3H), 2.69 (s, 1H), 2.57 (d, *J* = 17.1 Hz, 1H), 2.01 (s, 3H), 1.06 (d, *J* = 18.5 Hz, 6H). ^{13}C NMR (126 MHz, $CDCl_3$) δ 196.04, 170.54, 163.83, 151.70, 148.48, 139.05, 129.30, 128.41, 124.67, 117.81, 114.53, 112.35, 78.44, 77.25, 71.90, 70.61, 69.54, 67.63, 58.99, 49.67, 41.12, 24.29, 23.40, 21.47. HRMS (*m/z*) calcd. for $C_{23}H_{30}O_7$ $[M + Na]^+$ 441.1884, found 441.1874.

2. ^{1}H NMR, ^{13}C NMR and HRMS spectra of APA7~APA18

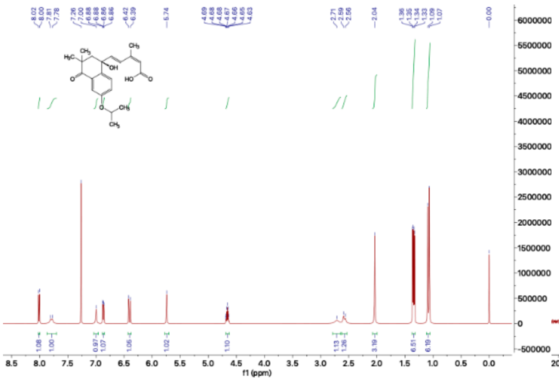

Figure S1. ^{1}HNMR of **APA7**

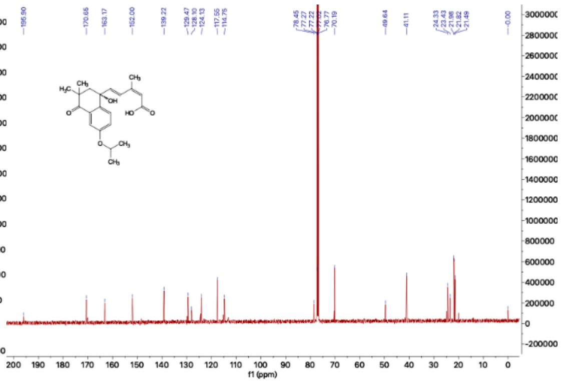

Figure S2. ^{13}CNMR of **APA7**

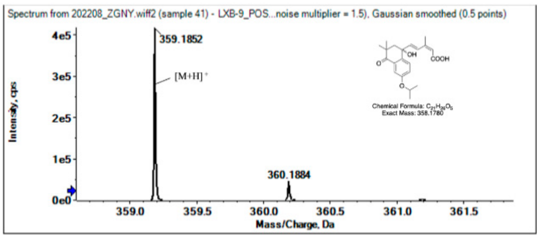

Figure S3. HRMS of **APA7**

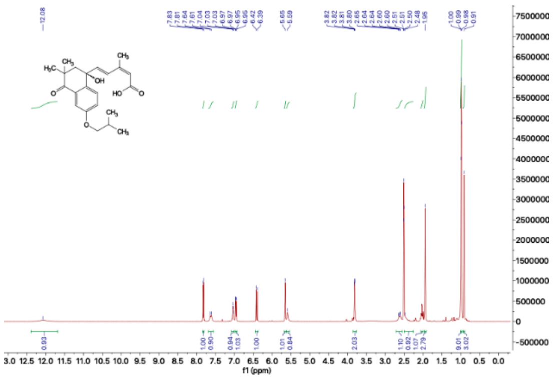

Figure S4. ^{1}HNMR of **APA8**

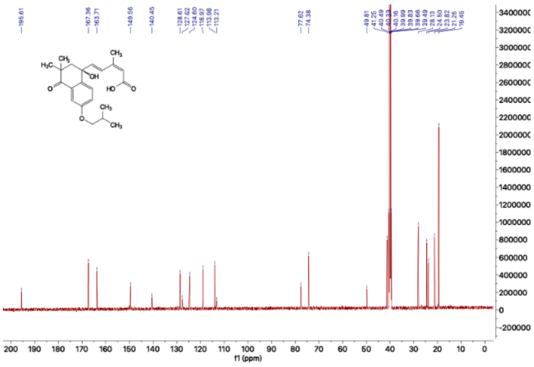

Figure S5. ^{13}CNMR of **APA8**

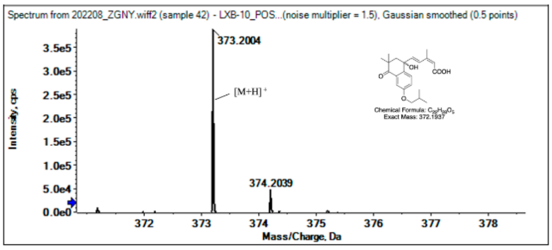

Figure S6. HRMS of **APA8**

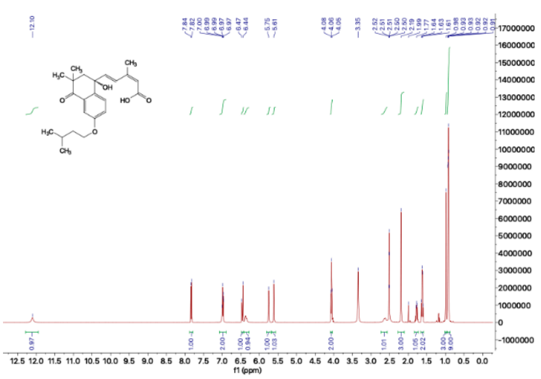

Figure S7. ^{1}HNMR of **APA9**

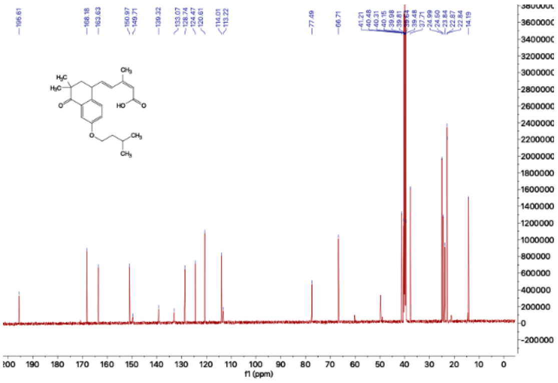

Figure S8. ^{13}CNMR of **APA9**

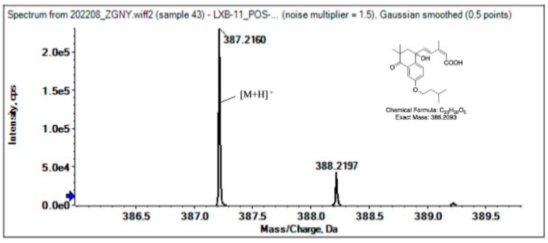

Figure S9. HRMS of **APA9**

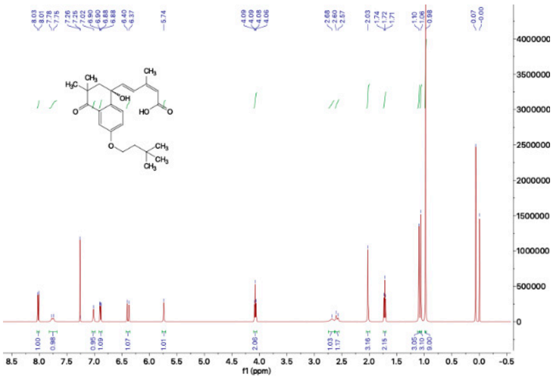

Figure S10. ^{1}HNMR of **APA10**

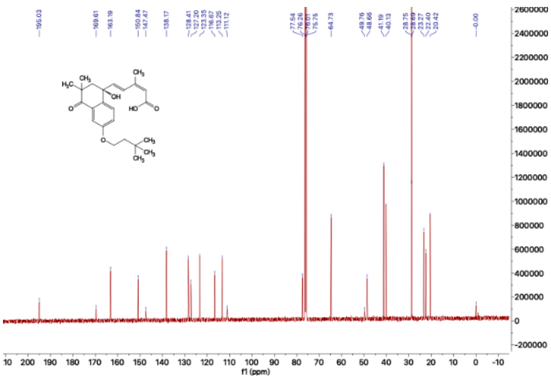

Figure S11. ^{13}CNMR of **APA10**

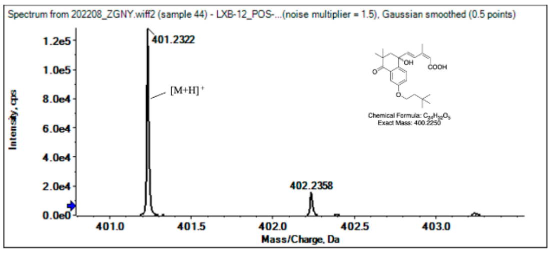

Figure S12. HRMS of **APA10**

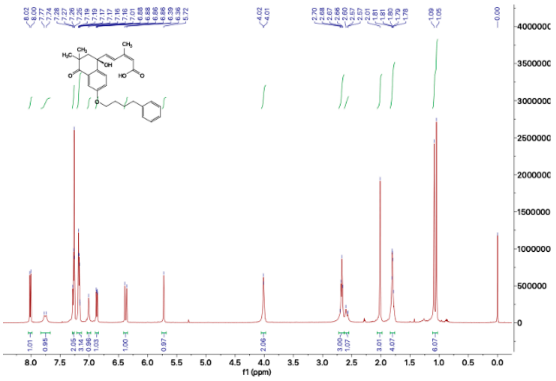

Figure S13. ^{1}HNMR of **APA11**

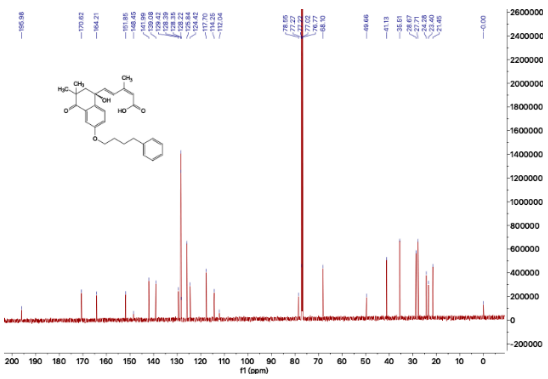

Figure S14. ^{13}CNMR of **APA11**

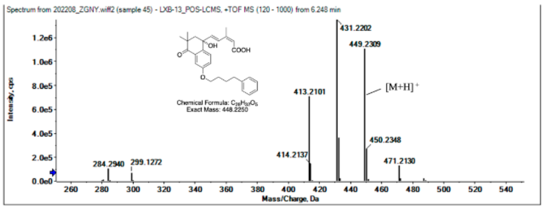

Figure S15. HRMS of **APA11**

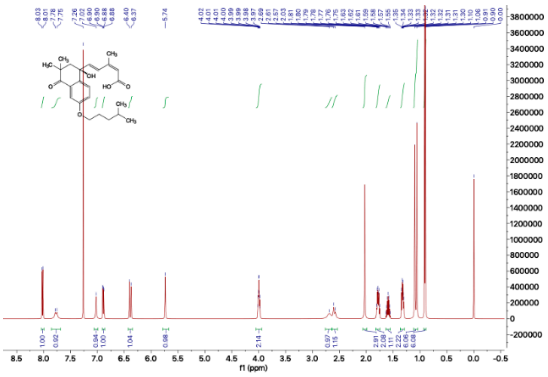

Figure S16. ^{1}HNMR of **APA12**

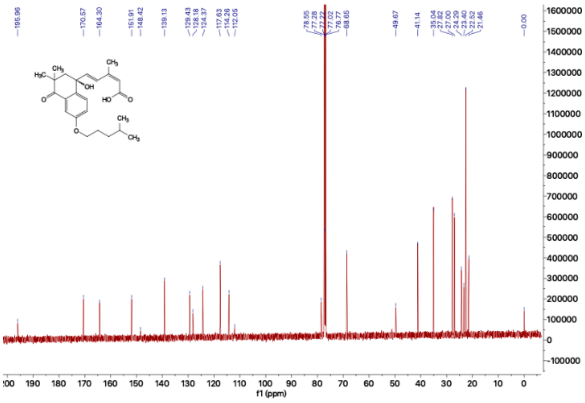

Figure S17. ^{13}CNMR of **APA12**

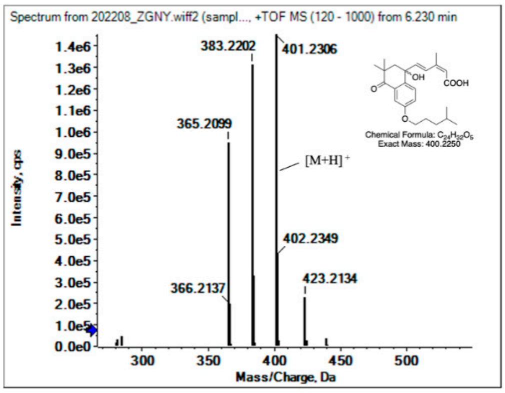

Figure S18. HRMS of **APA12**

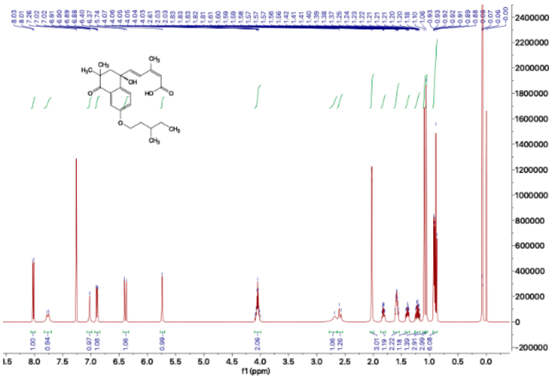

Figure S19. ^{1}HNMR of **APA13**

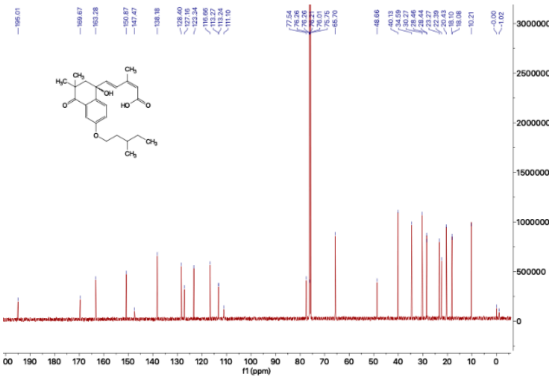

Figure S20. ^{13}CNMR of **APA13**

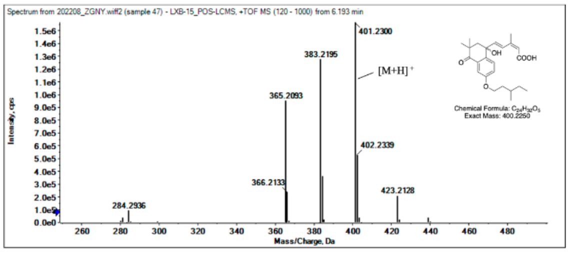

Figure S21. HRMS of **APA13**

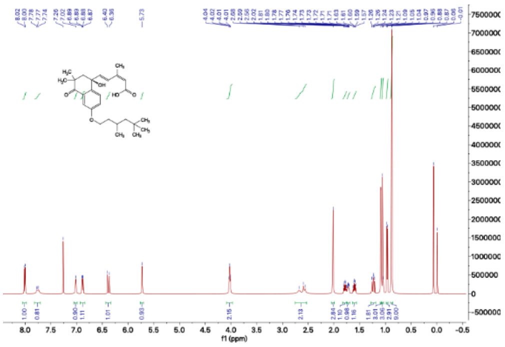

Figure S22. ^{1}HNMR of **APA14**

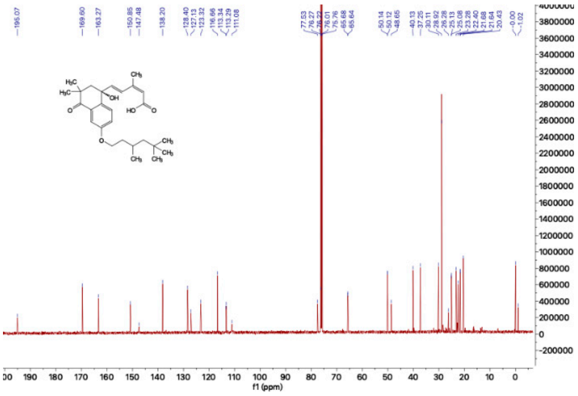

Figure S23. ^{13}CNMR of **APA14**

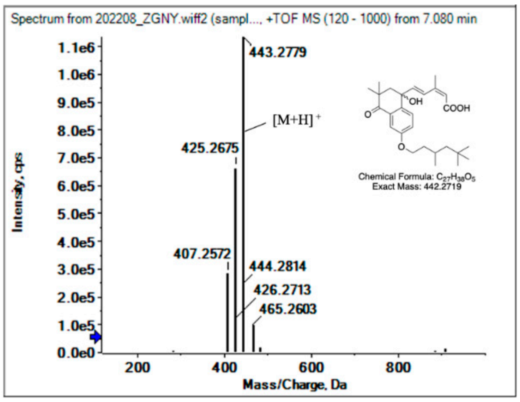

Figure S24. HRMS of **APA14**

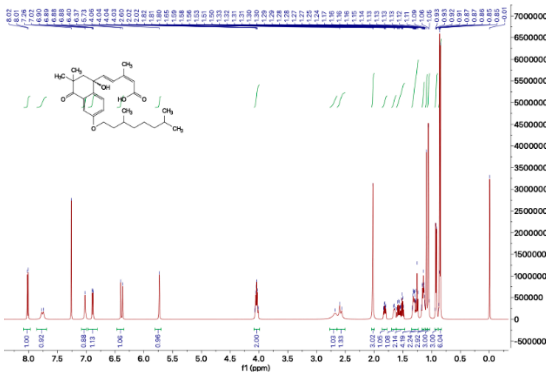

Figure S25. ^{1}HNMR of **APA15**

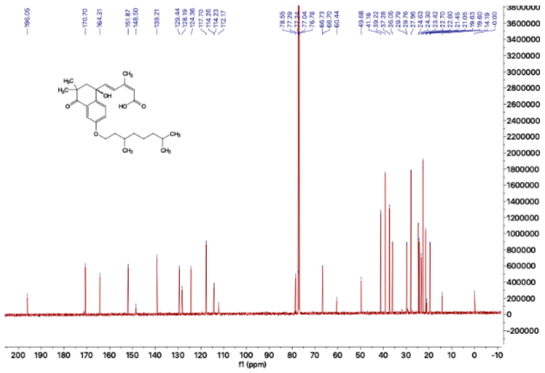

Figure S26. ^{13}CNMR of **APA15**

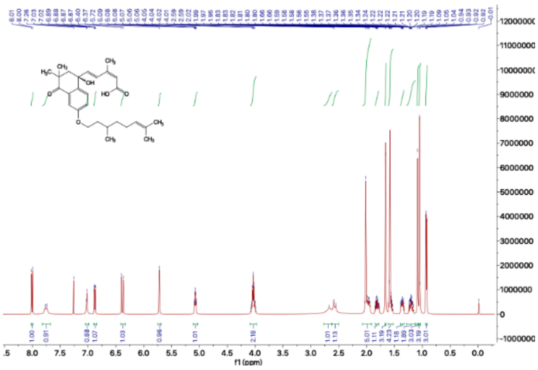

Figure S27. HRMS of **APA15**

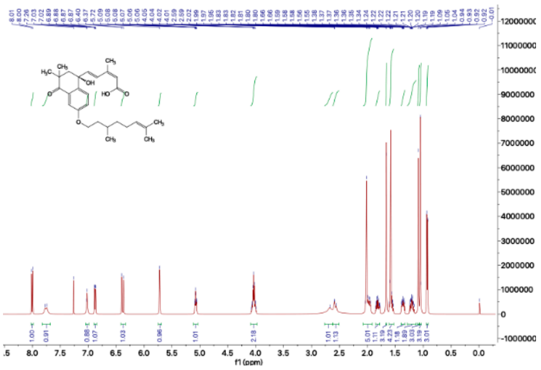

Figure S28. ^{1}HNMR of **APA16**

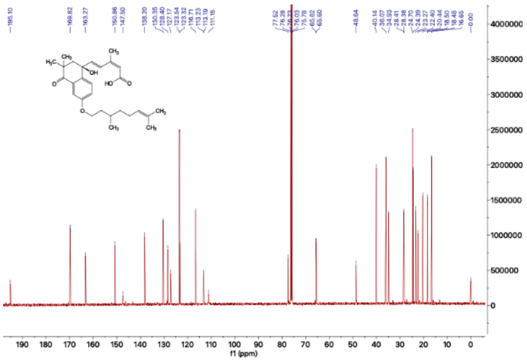

Figure S29. ^{13}CNMR of **APA16**

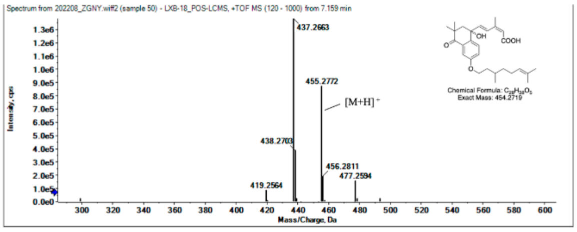

Figure S30. HRMS of **APA16**

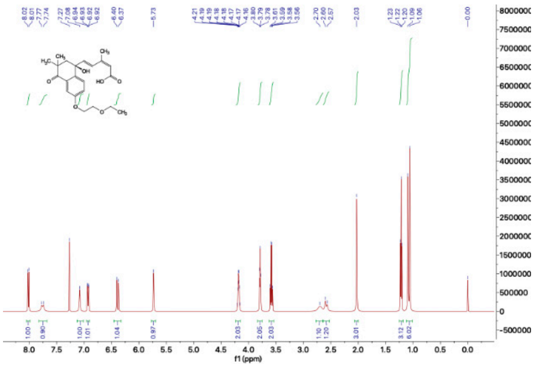

Figure S31. ^{1}HNMR of **APA17**

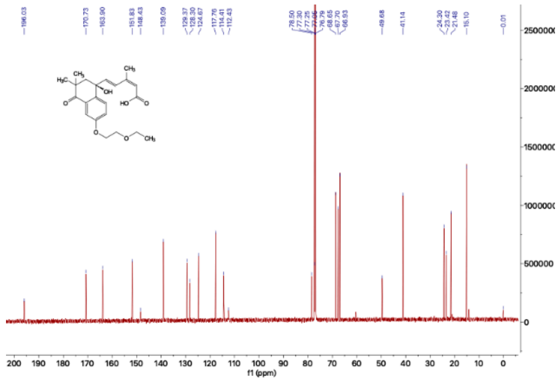

Figure S32. ^{13}CNMR of **APA17**

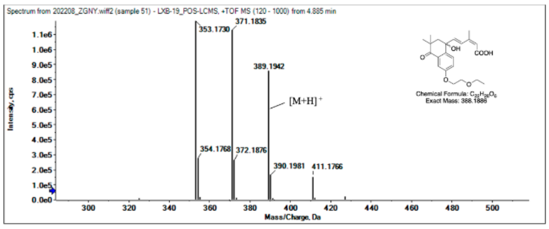

Figure S33. HRMS of **APA17**

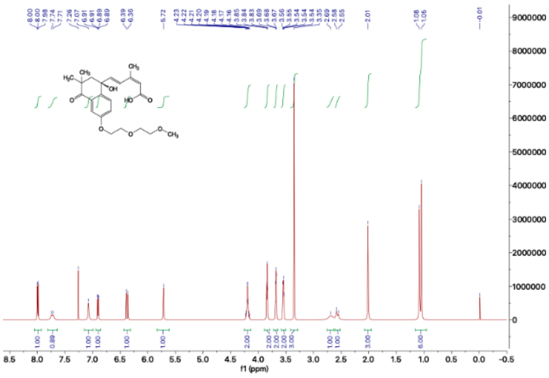

Figure S34. ^{1}HNMR of **APA18**

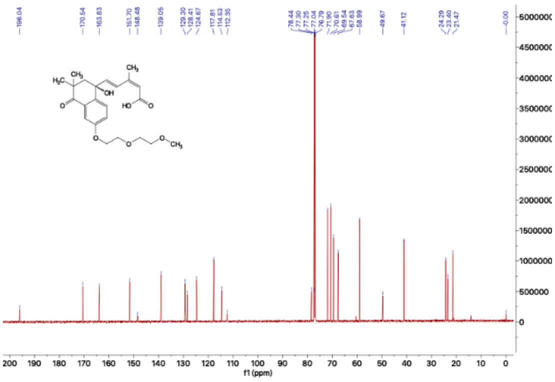

Figure S35. ^{13}CNMR of **APA18**

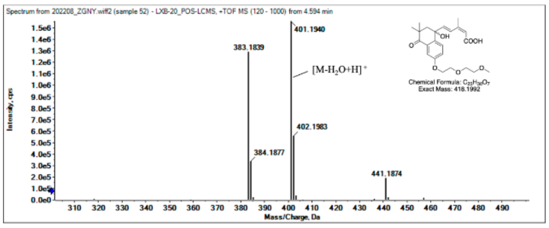

Figure S36. HRMS of **APA18**

4. MST analysis of the binding of APAn and control agents to ABA receptors

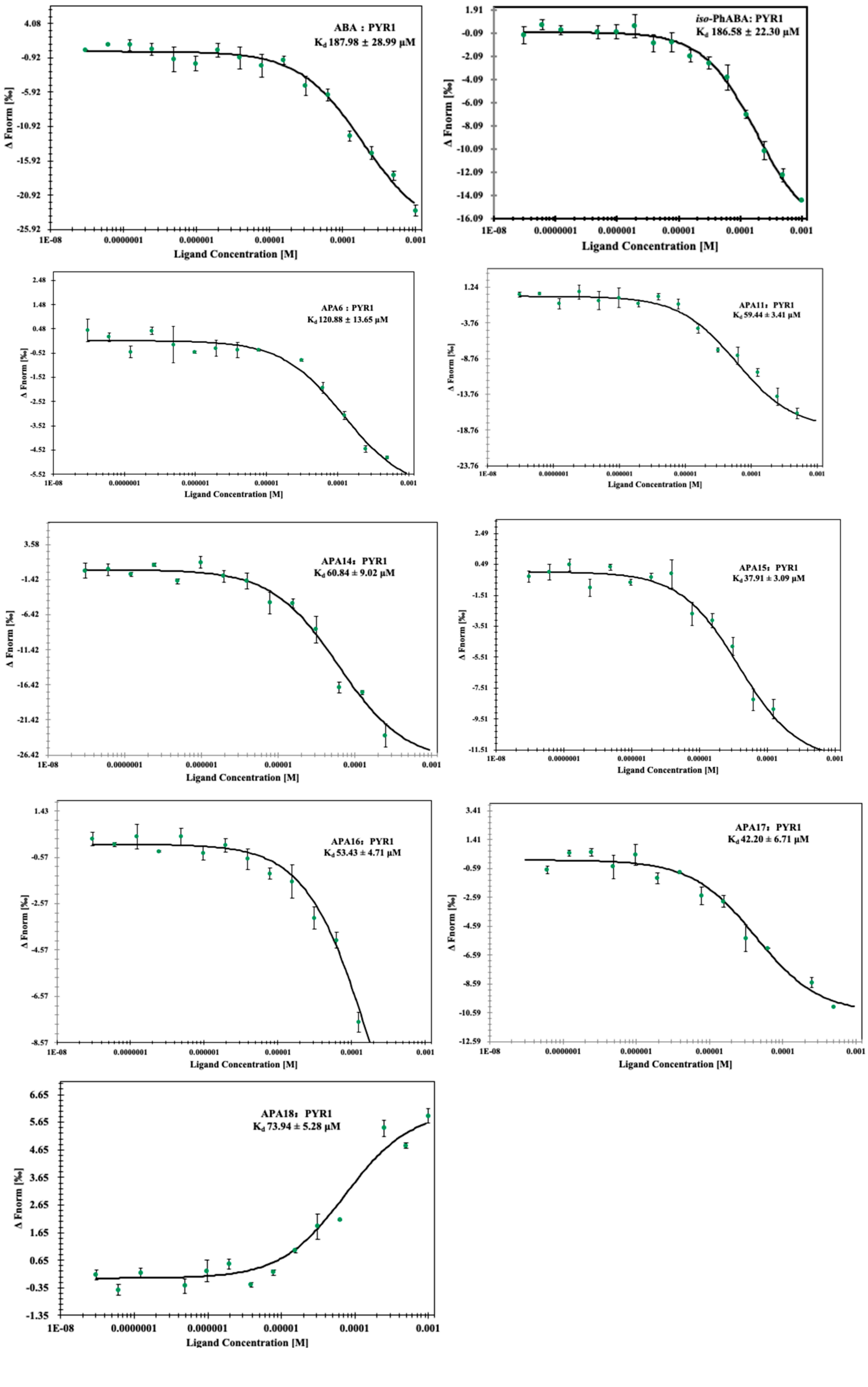

Figure S37. MST analysis of the binding of APAn and control agents to PYR1

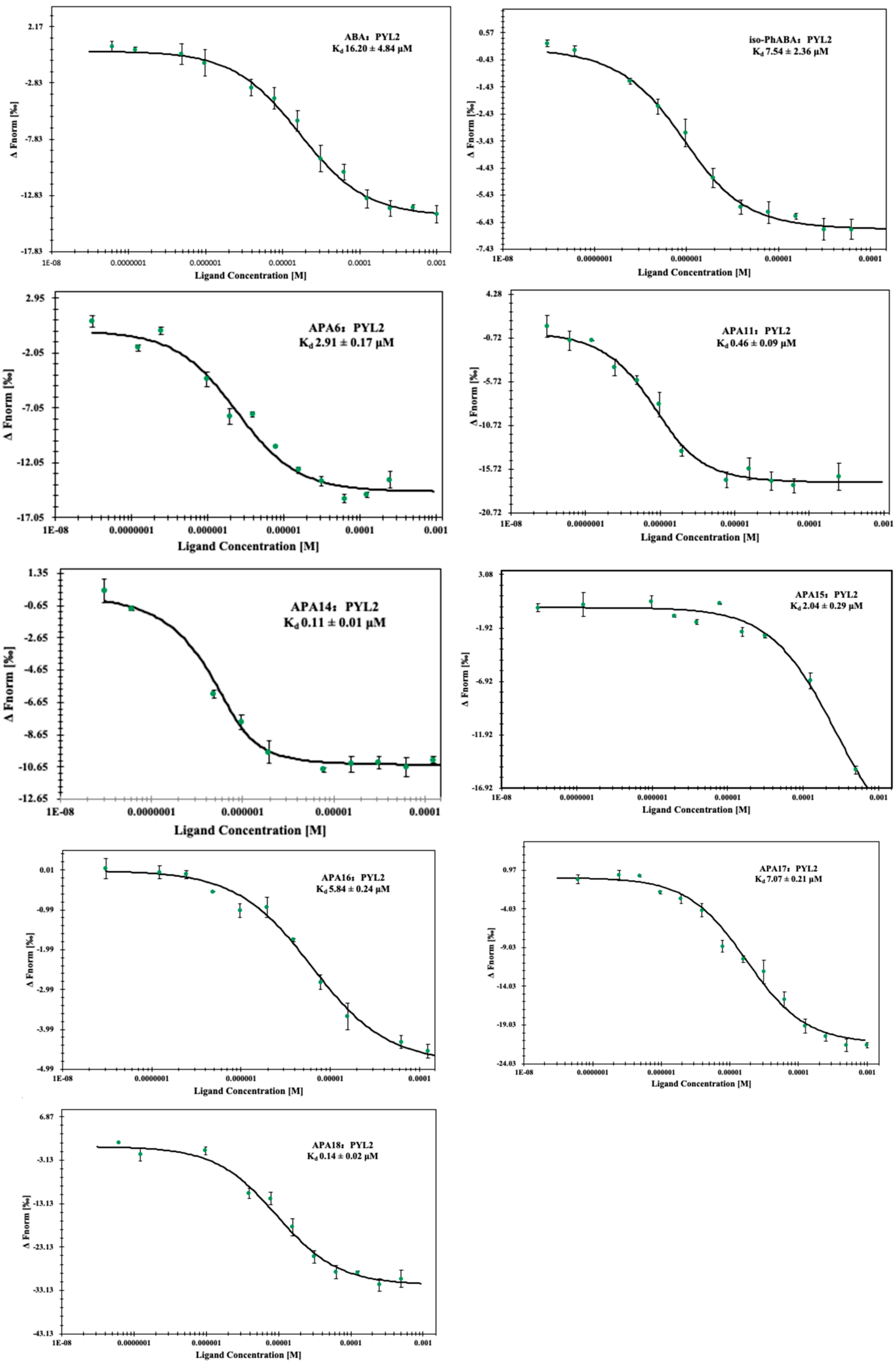

Figure S38. MST analysis of the binding of APAn and control agents to PYL2

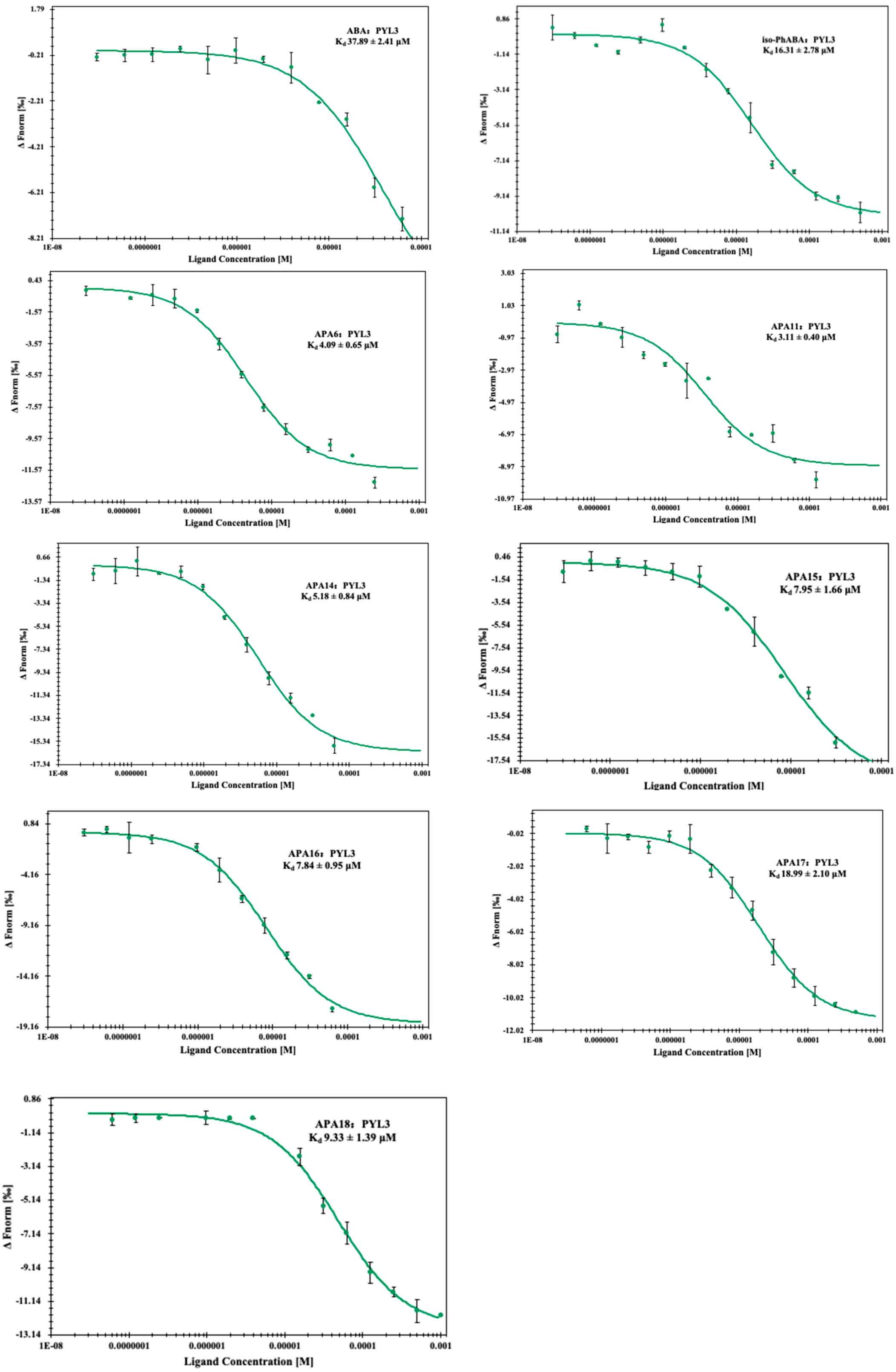

Figure S39. MST analysis of the binding of APAn and control agents to PYL3

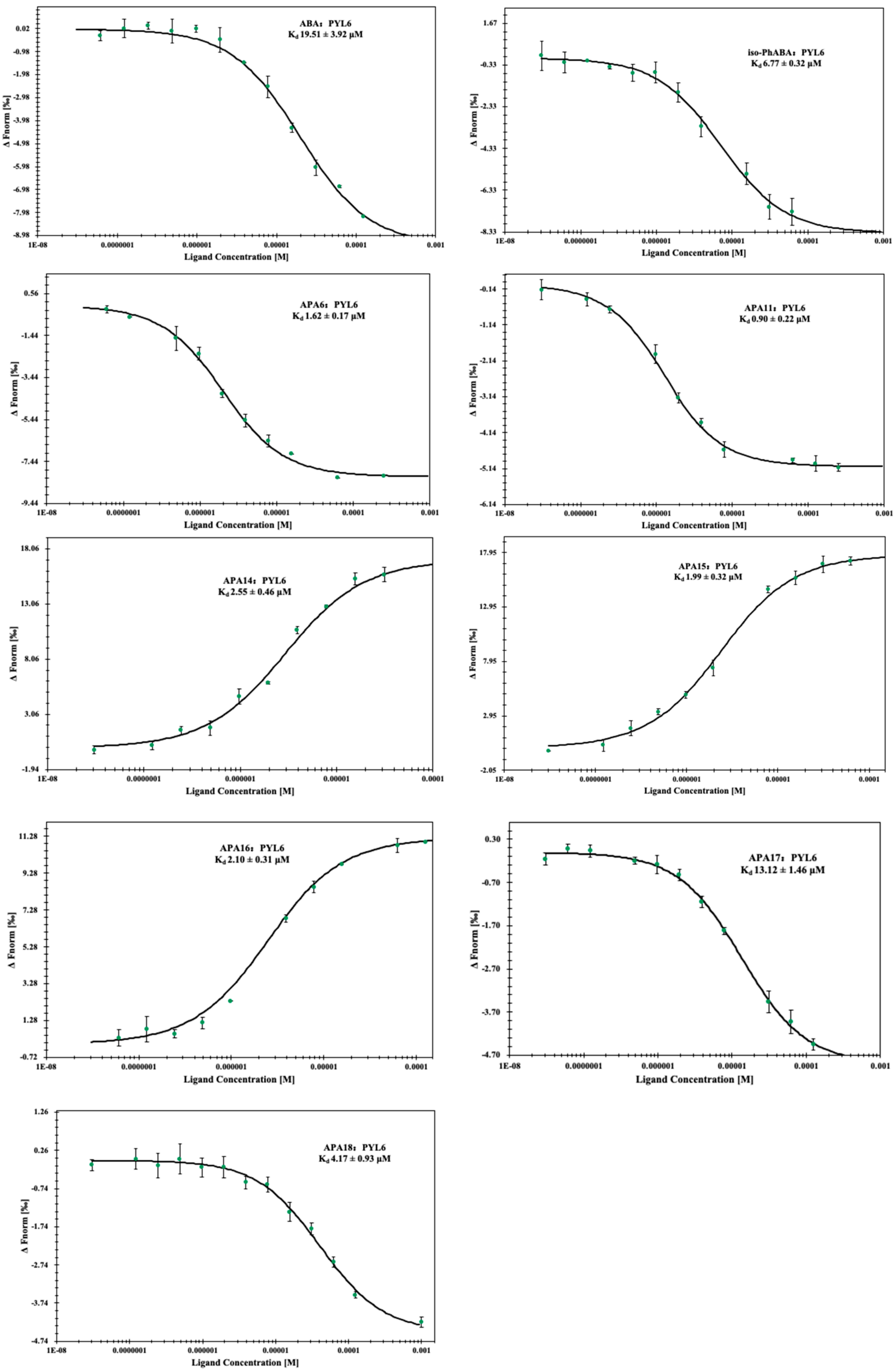

Figure S40. MST analysis of the binding of APAn and control agents to PYL6

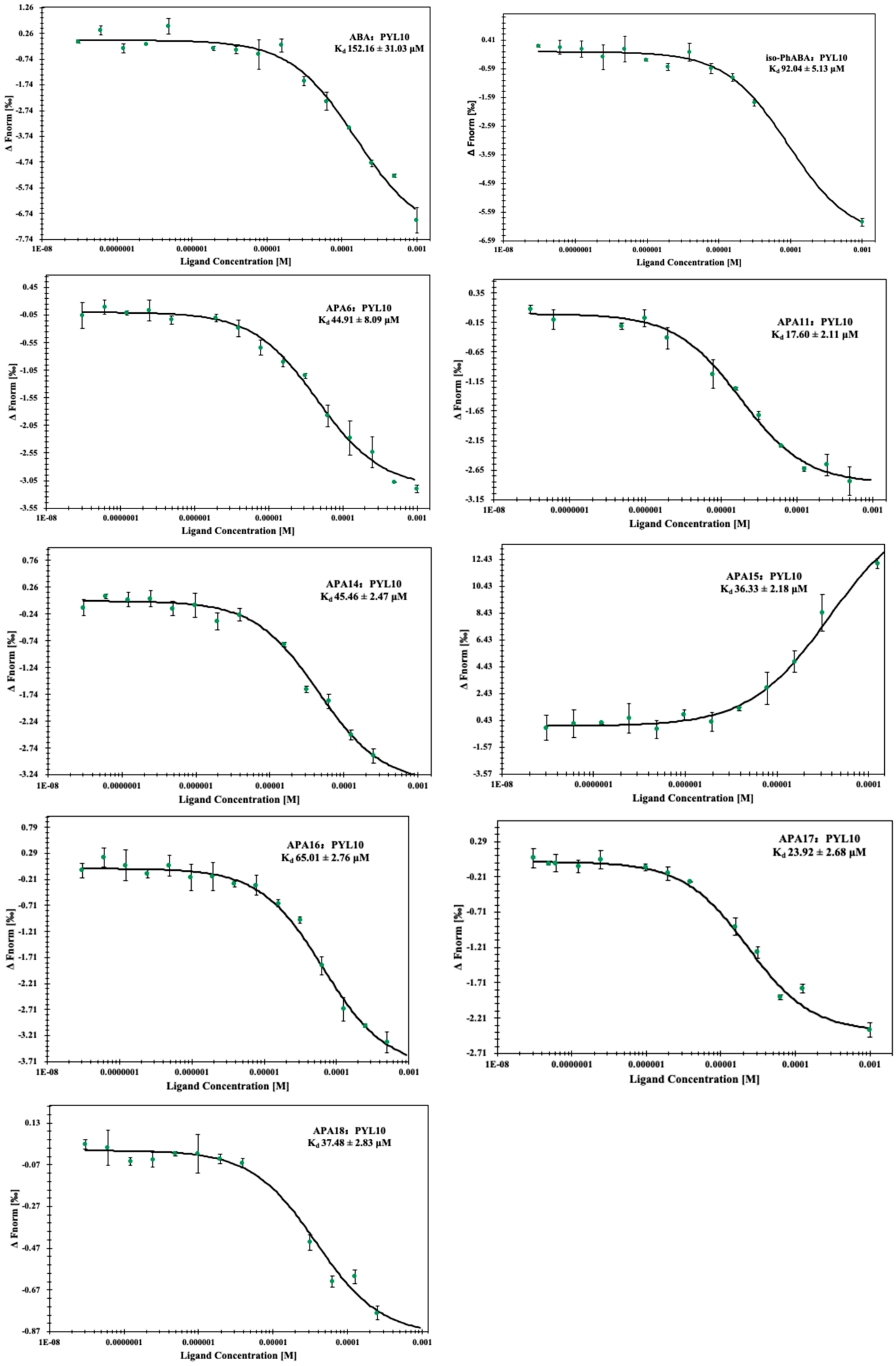

Figure S41. MST analysis of the binding of APAn and control agents to PYL10

4. Calculated values of the decomposition free energy

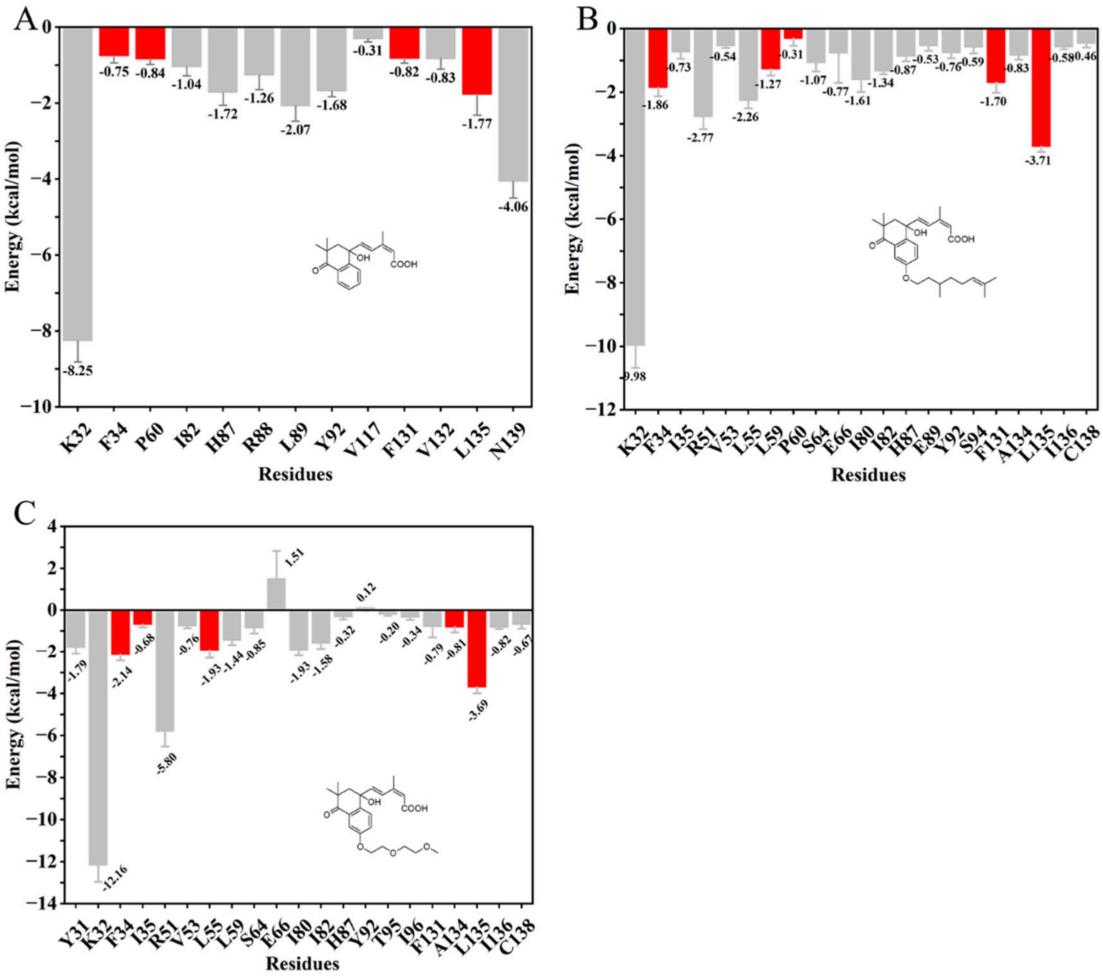

Figure S42. Calculated values of the decomposition free energy. (The red residues in the figure were hydrophobic residues around the 3'-tunnel.)

5. Seed germination promotion activity

Table S1. The effect of agent treatment on *A. thaliana* seed germination (48 h). Values represent means ± SD, and those marked with the same letter are statistically significant ($p < 0.05$).

Treatment 1[a]	Germination rate (%)	Treatment 2[b]	Germination rate (%)	Treatment 3[c]	Germination rate (%)
DMSO	91.33±2.31 A	DMSO	92.67±2.31 A	DMSO	93.33±3.06 A
ABA	13.33±1.15 G	ABA	31.33±3.06 F	ABA	10.67±3.06 G
iso-PhABA	8.67±3.06 G	*iso*-PhABA	22.67±1.15 GH	*iso*-PhABA	7.33±2.31 G
/	/	/	/	NaCl	48.67±6.11 E
APA1	32.00±3.46 F	ABA+APA1	11.33±3.06 I	APA1+NaCl	29.33±4.16 F
APA2	34.67±5.77 F	ABA+APA2	18.67±1.15 H	APA2+NaCl	33.33±5.03 F
APA3	56.00±2.00 E	ABA+APA3	25.33±3.06 G	APA3+NaCl	43.33±2.31 E
APA4	67.33±3.06 BCD	ABA+APA4	52.00±3.46 DE	APA4+NaCl	61.33±4.16 D
APA5	74.67±1.15 B	ABA+APA5	67.33±1.15 C	APA5+NaCl	71.33±2.31 C
APA6	85.33±3.06 A	ABA+APA6	78.67±3.06 B	APA6+NaCl	82.67±3.06 B
APA7	65.33±4.16 CD	ABA+APA7	48.00±2.00 E	APA7+NaCl	62.00±3.46 D
APA8	64.67±4.16 CD	ABA+APA8	50.67±4.16 DE	APA8+NaCl	62.00±5.29 D
APA9	64.67±9.45 CD	ABA+APA9	50.67±4.62 DE	APA9+NaCl	60.00±8.00 D
APA10	70.00±8.72 BCD	ABA+APA10	54.67±3.06 D	APA10+NaCl	61.33±3.06 D
APA11	84.67±4.16 A	ABA+APA11	75.33±6.11 B	APA11+NaCl	81.33±4.16 B
APA12	69.33±4.62 BCD	ABA+APA12	50.00±5.29 DE	APA12+NaCl	60.67±5.03 D
APA13	62.00±2.00 DE	ABA+APA13	46.67±4.16 E	APA13+NaCl	56.67±3.06 D
APA14	85.33±4.16 A	ABA+APA14	78.67±4.16 B	APA14+NaCl	86.00±2.00 B
APA15	85.33±3.06 A	ABA+APA15	74.67±1.15 B	APA15+NaCl	85.33±1.15 B
APA16	84.67±3.06 A	ABA+APA16	76.67±1.15 B	APA16+NaCl	84.00±4.00 B
APA17	83.33±3.06 A	ABA+APA17	75.33±3.06 B	APA17+NaCl	82.67±3.06 B
APA18	83.33±2.31 A	ABA+APA18	76.00±2.00 B	APA18+NaCl	84.00±3.46 B

[a] *A. thaliana* seeds were treated with 5 µM corresponding compound APAn alone. 5 µM ABA or 5 µM *iso*-PhABA treatment were used as control groups.

[b] *A. thaliana* seeds were treated with a mixture of 1 µM ABA and 5 µM APAn. 1 µM ABA or 1 µM *iso*-PhABA treatment were used as control groups.

[c] *A. thaliana* seeds were treated with the combination of 10 mM NaCl and 5 µM APAn. 5 µM ABA or 5 µM *iso*-PhABA treatment were used as control groups.

A

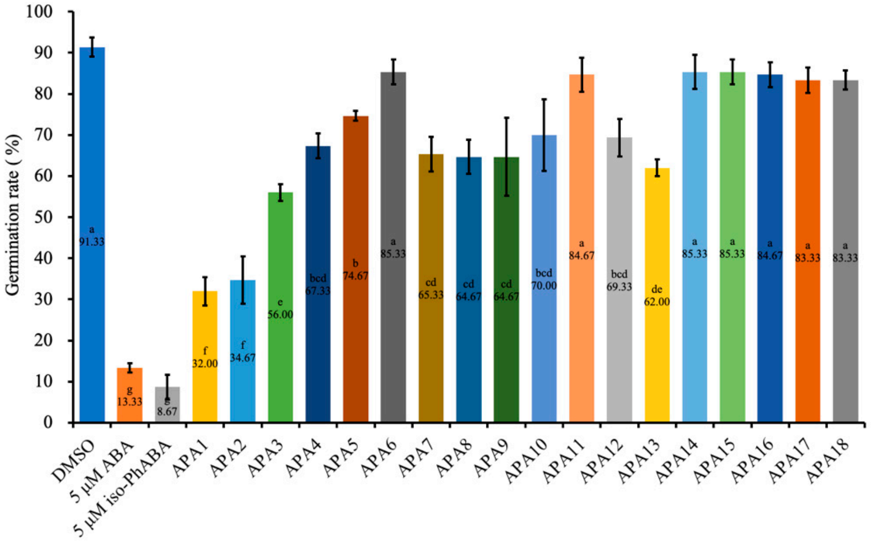

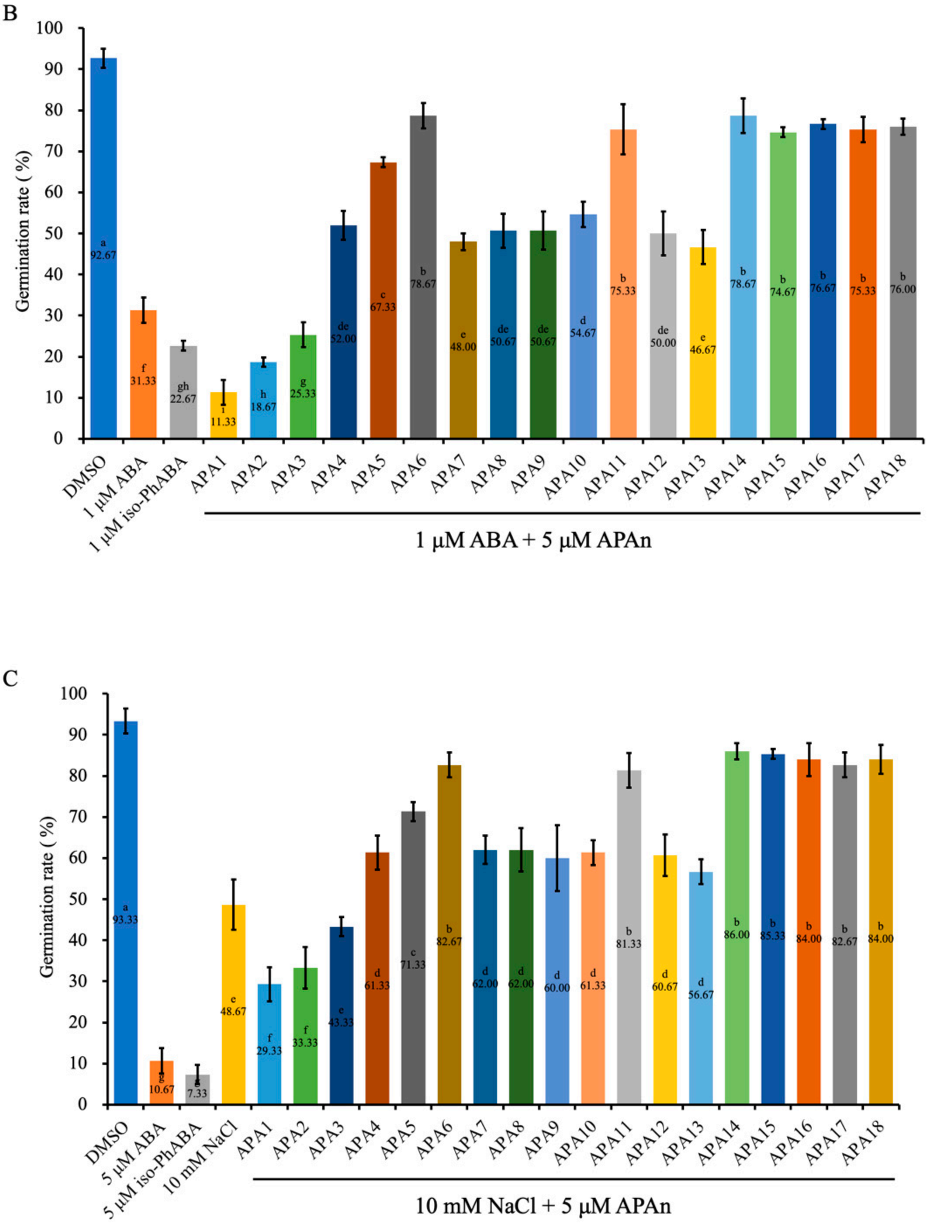

Figure S43. The effect of agent treatment on *A. thaliana* seed germination. Values represent means ± SD, and those marked with the same letter are statistically significant ($p < 0.05$).

Table S2. The effect of agent treatment on colza seed germination (48 h). Values represent means ± SD, and those marked with the same letter are statistically significant ($p < 0.05$).

**Treatment 1**[a]	**Germination rate (%)**	**Treatment 2**[b]	**Germination rate (%)**	**Treatment 3**[c]	**Germination rate (%)**
DMSO	91.77±2.75 ^{A}	DMSO	89.52±3.30 ^{A}	DMSO	91.43±5.71 ^{A}
ABA	18.70±3.92 ^{K}	ABA	49.52±3.30 ^{K}	ABA	2.86±2.86 ^{K}
iso-PhABA	13.60±3.29 ^{K}	*iso*-PhABA	21.90±4.36 ^{K}	*iso*-PhABA	0 ^{K}
/	/	/	/	NaCl	42.86±1.00 ^{H}
APA1	57.47±5.31 ^{J}	ABA+APA1	47.62±3.30 ^{J}	APA1+NaCl	25.71±5.71 ^{J}
APA2	66.37±3.04 ^{HI}	ABA+APA2	49.52±4.36 ^{HI}	APA2+NaCl	32.24±1.65 ^{IJ}
APA3	65.40±2.36 ^{I}	ABA+APA3	50.48±5.95 ^{I}	APA3+NaCl	34.29±7.56 ^{HIJ}
APA4	74.27±2.85 ^{EFG}	ABA+APA4	64.76±3.30 ^{EFG}	APA4+NaCl	37.14±7.56 ^{HI}
APA5	75.20±3.29 ^{EF}	ABA+APA5	72.38±4.36 ^{EF}	APA5+NaCl	62.86±4.95 ^{FG}
APA6	85.33±3.65 ^{ABC}	ABA+APA6	82.86±2.86 ^{ABC}	APA6+NaCl	77.14±2.86 ^{BCD}
APA7	67.63±1.67 ^{GHI}	ABA+APA7	56.19±4.36 ^{GHI}	APA7+NaCl	37.14±2.86 ^{HI}
APA8	68.90±4.70 ^{FGHI}	ABA+APA8	58.10±3.30 ^{FGHI}	APA8+NaCl	25.71±2.86 ^{J}
APA9	78.30±6.09 ^{CDE}	ABA+APA9	67.62±4.36 ^{CDE}	APA9+NaCl	65.71±8.57 ^{EFG}
APA10	78.10±5.98 ^{CDE}	ABA+APA10	68.57±2.86 ^{CDE}	APA10+NaCl	69.52±4.36 ^{DEF}
APA11	87.13±1.15 ^{AB}	ABA+APA11	80.95±3.30 ^{AB}	APA11+NaCl	77.14±4.95 ^{BCD}
APA12	72.67±1.48 ^{EFGH}	ABA+APA12	71.43±4.95 ^{EFGH}	APA12+NaCl	59.05±5.95 ^{G}
APA13	77.43±6.54 ^{DE}	ABA+APA13	65.71±4.95 ^{DE}	APA13+NaCl	69.52±5.95 ^{DEF}
APA14	86.20±4.56 ^{AB}	ABA+APA14	75.24±3.30 ^{AB}	APA14+NaCl	75.24±6.00 ^{CD}
APA15	83.93±1.79 ^{BCD}	ABA+APA15	75.24±4.36 ^{BCD}	APA15+NaCl	73.33±3.30 ^{CDE}
APA16	84.77±4.38 ^{ABCD}	ABA+APA16	76.19±4.36 ^{ABCD}	APA16+NaCl	80.95±4.36 ^{BC}
APA17	83.33±2.97 ^{BCD}	ABA+APA17	83.81±4.36 ^{BCD}	APA17+NaCl	85.71±7.56 ^{AB}
APA18	83.83±4.38 ^{BCD}	ABA+APA18	81.90±5.95 ^{BCD}	APA18+NaCl	82.86±4.95 ^{ABC}

[a] *Colza* seeds treated with 10 µM corresponding APAn compounds alone.

[b] *Colza* seeds treated both with 1 µM ABA and 10 µM corresponding APAn compounds, 1 µM ABA and 1 µM *iso*-PhABA treated as control groups.

[c] *Colza* seeds treated both with 25 mM NaCl and 25 µM corresponding APAn compounds, 25 µM ABA and 25 µM *iso*-PhABA treated as control groups.

A

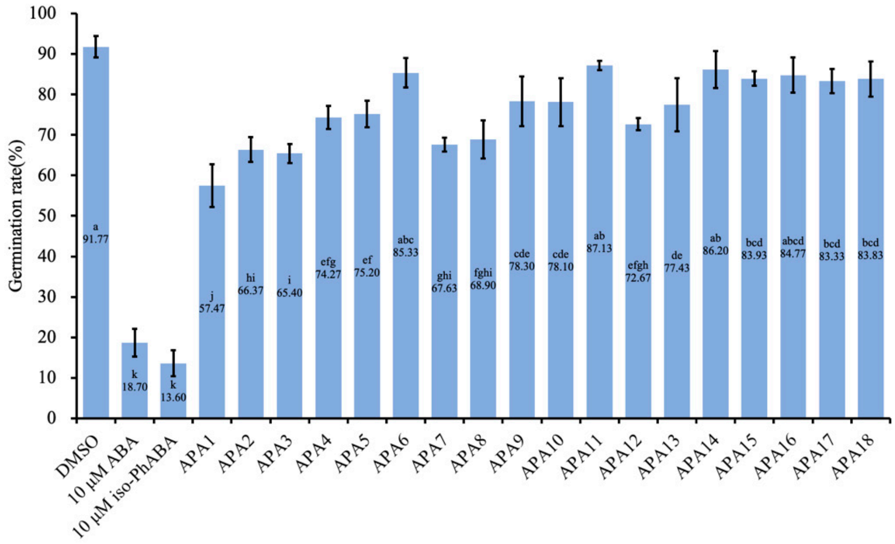

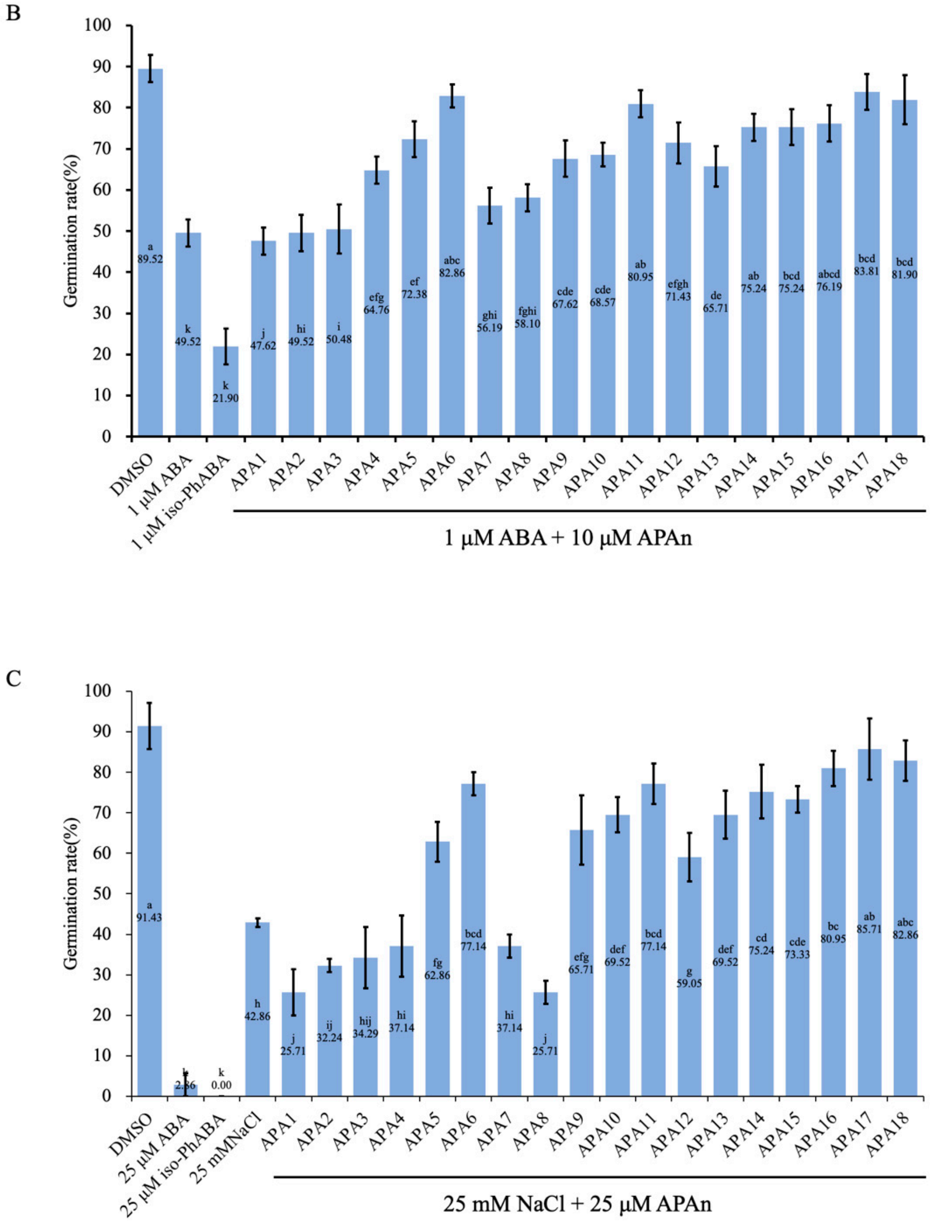

Figure S44. The effect of agent treatment on *colza* seed germination. Values represent means ± SD, and those marked with the same letter are statistically significant ($p < 0.05$).

Table S3. The germination parameters of rice seeds under different treatments. Values represent means ± SD, and those marked with the same letter are statistically significant ($p < 0.05$).

treatment		Germination	Germination	Germination	Cotyledons	Roots	Vitality
**compd.**	additive	potential(%)	rate(%)	index	length(cm)	length(cm)	index
DMSO	none	77.33±3.06 ^{A}	97.33±3.06 ^{AB}	15.34±0.44 ^{ABC}	6.19±0.67 ^{A}	8.08±0.57 ^{A}	94.93±10.13 ^{A}
10 μM ABA	none	36.00±4.00 ^{G}	97.33±2.31 ^{AB}	13.50±0.36 ^{E}	4.42±0.40 ^{F}	4.93±0.67 ^{G}	63.07±5.71 ^{DE}
10 μM *iso*-PhABA	none	18.00±2.00 ^{H}	98.00±1.46 ^{AB}	12.92±0.30 ^{F}	3.76±0.51 ^{G}	4.51±0.42 ^{G}	48.57±6.38 ^{E}
10 μM APA1	none	58.67±4.16 ^{F}	98.33±1.15 ^{A}	14.84±0.34 ^{ABCD}	5.25±0.47 ^{DE}	5.68±0.34 ^{F}	77.93±6.96 ^{BCD}
10 μM APA2	none	59.33±5.03 ^{F}	96.67±2.31 ^{AB}	14.46±0.48 ^{D}	5.51±0.27 ^{BCDE}	5.72±0.68 ^{F}	79.66±4.35 ^{BCD}
10 μM APA3	none	59.33±4.62 ^{F}	98.00±2.00 ^{AB}	14.71±0.46 ^{BCD}	5.13±0.25 ^{E}	6.09±0.94 ^{DEF}	75.43±4.04 ^{CDE}
10 μM APA4	none	67.33±3.06 ^{DE}	95.33±3.06 ^{AB}	14.67±0.34 ^{CD}	5.37±0.28 ^{CDE}	5.98±0.54 ^{EF}	78.80±4.22 ^{BCD}
10 μM APA5	none	68.67±4.16 ^{CDE}	96.67±1.15 ^{AB}	14.88±0.07 ^{ABCD}	5.39±0.53 ^{CDE}	6.64±0.63 ^{BCD}	80.20±7.68 ^{BCD}
10 μM APA6	none	73.33±2.31 ^{ABCD}	97.33±1.15 ^{AB}	15.17±0.26 ^{ABC}	5.81±0.46 ^{ABC}	6.99±0.52 ^{BC}	88.16±6.89 ^{ABC}
10 μM APA7	none	68.00±2.00 ^{CDE}	97.33 ±2.31 ^{AB}	14.98±0.31 ^{ABCD}	5.43±0.25 ^{BCDE}	6.36±0.59 ^{DE}	81.36±3.92 ^{BC}
10 μM APA8	none	68.67±6.43 ^{CDE}	97.33±1.15 ^{AB}	15.03±0.38 ^{ABCD}	5.52±0.46 ^{BCDE}	6.17±0.54 ^{DEF}	82.97±6.91 ^{ABC}
10 μM APA9	none	65.33±4.16 ^{EF}	96.67±1.15 ^{AB}	14.79±0.11 ^{ABCD}	5.58±0.59 ^{BCDE}	6.20±0.60 ^{DEF}	82.53±8.49 ^{ABC}
10 μM APA10	none	69.33±3.06 ^{BCDE}	95.33±1.15 ^{AB}	14.79±0.15 ^{ABCD}	5.18±0.20 ^{DE}	6.01±0.64 ^{EF}	76.61±2.98 ^{BCD}
10 μM APA11	none	73.33±3.06 ^{ABCD}	96.67±2.31 ^{AB}	15.12±0.19 ^{ABCD}	5.63±0.35 ^{BCD}	7.00±0.81 ^{BC}	85.14±5.18 ^{ABC}
10 μM APA12	none	74.67±4.62 ^{ABCD}	98.67±1.15 ^{A}	15.41±0.19 ^{A}	5.75±0.52 ^{ABC}	7.11±0.57 ^{BC}	88.61±7.81 ^{AB}
10 μM APA13	none	68.67±3.06 ^{CDE}	96.67±3.06 ^{AB}	14.93±0.50 ^{ABCD}	5.44±0.38 ^{BCDE}	6.52±0.51 ^{CDE}	81.20±5.93 ^{BC}
10 μM APA14	none	77.33±2.31 ^{A}	94.00±2.00 ^{B}	14.94±0.24 ^{ABCD}	5.75±0.49 ^{ABC}	6.98±0.71 ^{BC}	85.91±7.13 ^{ABC}
10 μM APA15	none	74.67±4.62 ^{ABCD}	98.67±1.15 ^{A}	15.41±0.37 ^{A}	5.73±0.51 ^{BC}	7.17±0.40 ^{B}	88.30±7.84 ^{ABC}
10 μM APA16	none	76.67±6.11 ^{AB}	95.33±3.06 ^{AB}	15.36±0.64 ^{AB}	5.62±0.39 ^{BCD}	7.07±0.56 ^{BC}	86.34±6.55 ^{ABC}
10 μM APA17	none	75.33±4.16 ^{ABC}	96.67±1.15 ^{A}	15.42±0.36 ^{A}	5.78±0.60 ^{ABC}	7.12±0.46 ^{BC}	89.13±9.09 ^{AB}
10 μM APA18	none	76.67±3.06 ^{AB}	96.67±1.15 ^{AB}	15.26±0.24 ^{ABC}	5.87±0.36 ^{AB}	7.10±0.67 ^{BC}	89.58±5.46 ^{AB}
DMSO	none	77.33±3.06 ^{A}	97.33±3.06 ^{AB}	15.34±0.44 ^{A}	6.19±0.67 ^{A}	8.08±0.57 ^{A}	94.93±10.13 ^{A}
1 μM ABA	none	51.33±2.31 ^{DE}	96.67±3.06 ^{AB}	14.27±0.37 ^{BCDEF}	5.03±0.23 ^{EF}	6.27±0.42 ^{EF}	67.91±3.30 ^{D}
1 μM *iso*-PhABA	none	26.67±8.33 ^{G}	97.33±2.31 ^{AB}	13.33±0.71 ^{H}	4.86±0.47 ^{F}	5.43±0.35 ^{H}	64.77±6.75 ^{E}
10 μM APA1	1 μM ABA	41.33±2.31 ^{F}	97.33±1.06 ^{AB}	13.86±0.43 ^{EFGH}	4.85±0.62 ^{F}	5.41±0.44 ^{H}	67.20±8.48 ^{DE}
10 μM APA2	1 μM ABA	45.33±1.15 ^{EF}	97.33±1.15 ^{AB}	14.02±0.21 ^{DEFGH}	5.41±0.19 ^{CDE}	5.59±0.28 ^{GH}	75.87±2.76 ^{BCDE}
10 μM APA3	1 μM ABA	48.67±5.03 ^{DEF}	96.00±2.00 ^{AB}	14.01±0.48 ^{DEFGH}	5.15±0.28 ^{DEF}	6.01±0.65 ^{FG}	72.15±4.32 ^{DE}
10 μM APA4	1 μM ABA	54.67±1.15 ^{CD}	97.33±2.31 ^{AB}	14.43±0.24 ^{BCDEF}	5.45±0.48 ^{CDE}	6.02±0.60 ^{FG}	78.63±6.76 ^{BCD}
10 μM APA5	1 μM ABA	54.67±4.16 ^{CD}	96.00±2.00 ^{AB}	14.24±0.40 ^{BCDEF}	5.63±0.52 ^{BCD}	6.76±0.59 ^{BCDE}	80.19±7.43 ^{BCD}
10 μM APA6	1 μM ABA	62.00±2.00 ^{BC}	96.67±2.31 ^{A}	14.88±0.27 ^{AB}	5.93±0.32 ^{ABC}	7.01±0.50 ^{BC}	88.26±4.79 ^{AB}
10 μM APA7	1 μM ABA	46.00±3.46 ^{EF}	94.67±3.06 ^{AB}	13.73±0.53 ^{FGH}	5.65±0.51 ^{BCD}	6.37±0.61 ^{DEF}	77.59±7.26 ^{BCD}
10 μM APA8	1 μM ABA	44.67±4.16 ^{EF}	95.33±3.06 ^{AB}	13.99±0.34 ^{DEFGH}	5.74±0.75 ^{ABC}	6.13±0.60 ^{FG}	80.34±10.2 ^{BCD}
10 μM APA9	1 μM ABA	50.67±3.06 ^{DE}	96.00±2.00 ^{AB}	14.09±0.16 ^{CDEFG}	5.18±0.39 ^{DEF}	6.10±0.81 ^{FG}	73.00±5.29 ^{CDE}
10 μM APA10	1 μM ABA	42.67±6.11 ^{F}	94.00±2.00 ^{B}	13.51±0.07 ^{GH}	5.01±0.70 ^{EF}	6.28±0.53 ^{EF}	67.69±9.09 ^{DE}
10 μM APA11	1 μM ABA	60.67±3.06 ^{BC}	95.33±3.06 ^{AB}	14.69±0.50 ^{ABCD}	5.91±0.32 ^{ABC}	6.97±0.51 ^{BC}	86.86±5.18 ^{ABC}
10 μM APA12	1 μM ABA	55.33±2.31 ^{CD}	95.33±3.06 ^{AB}	14.47±0.46 ^{BCDE}	5.64±0.63 ^{BCD}	6.92±0.78 ^{BCD}	81.63±9.00 ^{ABCD}
10 μM APA13	1 μM ABA	46.00±5.29 ^{EF}	97.33±2.31 ^{AB}	14.08±0.42 ^{CDEFG}	5.43±0.37 ^{CDE}	6.55±0.89 ^{CDEF}	76.47±5.31 ^{BCDE}
10 μM APA14	1 μM ABA	62.00±6.00 ^{BC}	97.33±1.15 ^{AB}	14.73±0.36 ^{ABCD}	6.02±0.63 ^{AB}	7.27±0.67 ^{B}	88.67±9.09 ^{AB}
10 μM APA15	1 μM ABA	60.00±5.29 ^{BC}	98.00±0.00 ^{AB}	14.72±0.25 ^{ABCD}	6.01±0.81 ^{AB}	7.25±0.46 ^{B}	88.45±11.55 ^{AB}
10 μM APA16	1 μM ABA	59.33±1.15 ^{BC}	98.00±0.00 ^{AB}	14.69±0.05 ^{ABCD}	6.11±0.69 ^{AB}	7.06±0.51 ^{BC}	89.76±9.74 ^{AB}
10 μM APA17	1 μM ABA	64.00±4.00 ^{B}	97.33±1.15 ^{AB}	14.83±0.22 ^{AB}	6.06±0.55 ^{AB}	7.16±0.68 ^{B}	89.89±8.01 ^{AB}
10 μM APA18	1 μM ABA	62.67±4.62 ^{BC}	97.33±1.15 ^{AB}	14.78±0.29 ^{ABC}	6.12±0.46 ^{AB}	7.08±0.45 ^{BC}	90.43±6.78 ^{AB}
DMSO	none	77.33±3.06 ^{A}	97.33±3.06 ^{A}	15.34±0.44 ^{A}	6.19±0.67 ^{A}	8.08±0.57 ^{A}	94.93±10.13 ^{A}
50 μM ABA	none	0 ^{I}	97.33±2.31 ^{A}	11.28±0.33 ^{G}	4.10±0.77 ^{I}	4.48±0.77 ^{J}	46.26±8.49 ^{H}
50 μM *iso*-PhABA	none	0 ^{I}	49.33±3.06 ^{B}	5.42±0.33 ^{H}	3.41±0.84 ^{J}	2.58±0.39 ^{K}	18.47±4.47 ^{I}
none	100 mM NaCl	46.67±1.15 ^{B}	96.00±0.00 ^{A}	13.94±0.05 ^{AB}	4.93±0.51 ^{GH}	5.71±0.85 ^{FGHI}	68.76±6.81 ^{FG}
50 μM APA1	100 mM NaCl	33.33±6.11 ^{EF}	97.33±2.31 ^{A}	13.46±0.35 ^{EF}	4.62±0.81 ^{H}	5.27±0.75 ^{I}	62.17±10.59 ^{G}
50 μM APA2	100 mM NaCl	42.00±2.00 ^{H}	98.67±1.15 ^{A}	14.05±0.25 ^{F}	5.22±0.71 ^{EFG}	5.38±0.74 ^{HI}	73.34±9.68 ^{FG}
50 μM APA3	100 mM NaCl	45.33±3.06 ^{FG}	97.33±1.15 ^{A}	14.02±0.28 ^{DEF}	5.35±0.60 ^{EFG}	5.90±0.63 ^{FGHI}	75.02±8.20 ^{EF}
50 μM APA4	100 mM NaCl	51.33±2.31 ^{EFG}	94.67±3.06 ^{A}	13.96±0.49 ^{DEF}	5.56±0.36 ^{DEF}	6.25±0.75 ^{FG}	77.58±5.32 ^{BCDEF}
50 μM APA5	100 mM NaCl	60.00±3.46 ^{DE}	97.33±2.31 ^{A}	14.62±0.25 ^{EF}	5.47±0.36 ^{EF}	6.51±0.77 ^{CDEF}	80.06±5.25 ^{BCDEF}
50 μM APA6	100 mM NaCl	64.00±4.00 ^{BC}	95.33±1.15 ^{A}	14.55±0.31 ^{BCD}	6.11±0.70 ^{ABC}	7.37±0.61 ^{AB}	88.90±9.89 ^{ABCD}
50 μM APA7	100 mM NaCl	48.00±2.00 ^{B}	98.00±2.00 ^{A}	14.20±0.30 ^{BCDE}	5.63±0.43 ^{BCDE}	6.40±0.78 ^{DEFG}	79.95±6.02 ^{BCDEF}
50 μM APA8	100 mM NaCl	40.00±5.29 ^{EF}	96.00±2.00 ^{A}	13.60±0.47 ^{CDE}	5.61±0.49 ^{BCDEF}	6.09±0.73 ^{FGH}	76.30±6.78 ^{DEF}
50 μM APA9	100 mM NaCl	46.67±3.06 ^{G}	96.67±2.31 ^{A}	14.01±0.31 ^{F}	5.52±0.42 ^{EF}	6.29±0.78 ^{EFG}	77.34±5.87 ^{CDEF}
50 μM APA10	100 mM NaCl	54.67±3.06 ^{EF}	96.67±2.31 ^{A}	14.54±0.28 ^{DEF}	5.08±0.55 ^{FGH}	5.63±0.55 ^{GHI}	73.74±7.87 ^{FG}
50 μM APA11	100 mM NaCl	61.33±1.15 ^{CD}	96.00±2.00 ^{A}	14.51±0.29 ^{BCDE}	6.12±0.39 ^{ABC}	7.13±0.92 ^{BCD}	88.76±5.67 ^{ABCD}
50 μM APA12	100 mM NaCl	66.00±4.00 ^{B}	96.00±2.00 ^{A}	14.67±0.21 ^{BCDE}	5.58±0.46 ^{CDEF}	7.05±1.11 ^{BCDE}	81.74±6.62 ^{BCDEF}

50 μM APA13	100 mM NaCl	50.00±6.00 B	98.67±1.15 A	14.37±0.29 BC	6.10±0.37 ABC	7.28±1.18 BC	87.62±5.27 ABCDE
50 μM APA14	100 mM NaCl	64.00±4.00 DE	97.33±1.15 A	14.82±0.06 BCDE	6.12±0.32 ABC	7.34±0.58 AB	90.68±4.57 ABC
50 μM APA15	100 mM NaCl	60.00±4.00 B	96.67±1.15 A	14.55±0.06 ABC	6.16±0.28 AB	7.42±0.65 AB	89.63±3.94 ABCD
50 μM APA16	100 mM NaCl	60.67±3.06 BC	96.00±4.00 A	14.51±0.44 BCDE	6.06±0.34 ABCD	7.46±1.03 AB	87.95±5.29 ABCDE
50 μM APA17	100 mM NaCl	61.33±1.15 B	98.00±2.00 A	14.72±0.30 BCDE	6.15±0.44 AB	7.19±0.84 BCD	90.53±6.41 ABC
50 μM APA18	100 mM NaCl	66.00±3.46 B	96.67±1.15 A	14.83±0.58 ABC	6.13±0.17 AB	7.18±0.97 BCD	90.95±3.82 AB
